# Cautionary tales on the use of proxies to estimate body size and form of extinct animals

**DOI:** 10.1002/ece3.70218

**Published:** 2024-09-02

**Authors:** Joel H. Gayford, Russell K. Engelman, Phillip C. Sternes, Wayne M. Itano, Mohamad Bazzi, Alberto Collareta, Rodolfo Salas‐Gismondi, Kenshu Shimada

**Affiliations:** ^1^ Department of Life Sciences, Silwood Park Campus Imperial College London London UK; ^2^ Department of Marine Biology and Aquaculture James Cook University Douglas Queensland Australia; ^3^ Shark Measurements London UK; ^4^ Department of Biology Case Western Reserve University Cleveland Ohio USA; ^5^ Department of Evolution, Ecology and Organismal Biology University of California Riverside California USA; ^6^ Museum of Natural History University of Colorado Boulder Colorado USA; ^7^ Department of Earth and Planetary Sciences Stanford University Stanford California USA; ^8^ Dipartimento di Scienze Della Terra Università di Pisa Pisa Italy; ^9^ Museo di Storia Naturale Università di Pisa Pisa Italy; ^10^ Laboratorios de Investigación y Desarrollo, Facultad de Ciencias y Filosofía/Centro de Investigación Para el Desarrollo Integral y Sostenible Universitad Peruana Cayetano Heredia Lima Lima Peru; ^11^ Departamento de Paleontología de Vertebrados Museo de Historia Natural‐Universidad Nacional Mayor de san Marcos Lima Peru; ^12^ Department of Environmental Science and Studies DePaul University Chicago Illinois USA; ^13^ Department of Biological Sciences DePaul University Chicago Illinois USA; ^14^ Sternberg Museum of Natural History Fort Hays State University Hays Kansas USA

**Keywords:** allometric scaling, body shape, evolution, fossil, morphology, palaeobiology

## Abstract

Body size is of fundamental importance to our understanding of extinct organisms. Physiology, ecology and life history are all strongly influenced by body size and shape, which ultimately determine how a species interacts with its environment. Reconstruction of body size and form in extinct animals provides insight into the dynamics underlying community composition and faunal turnover in past ecosystems and broad macroevolutionary trends. Many extinct animals are known only from incomplete remains, necessitating the use of anatomical proxies to reconstruct body size and form. Numerous limitations affecting the appropriateness of these proxies are often overlooked, leading to controversy and downstream inaccuracies in studies for which reconstructions represent key input data. In this perspective, we discuss four prominent case studies (*Dunkleosteus*, *Helicoprion*, Megalodon and *Perucetus*) in which proxy taxa have been used to estimate body size and shape from fragmentary remains. We synthesise the results of these and other studies to discuss nuances affecting the validity of taxon selection when reconstructing extinct organisms, as well as mitigation measures that can ensure the selection of the most appropriate proxy. We argue that these precautionary measures are necessary to maximise the robustness of reconstructions in extinct taxa for better evolutionary and ecological inferences.

## Graphical abstract

Reconstructing the body size and form of extinct animals is of vital importance to our understanding of macroevolution and palaeontology. This is often done using anatomical proxies where extinct species are known only from fragmentary remains. However, there are many limitations influencing the selection of proxy taxa that are frequently overlooked. Here, we discuss these limitations and mitigation measures that should be applied to overcome them.

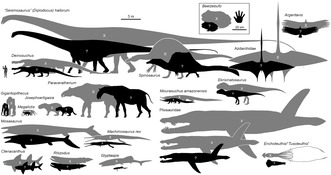

## 1 INTRODUCTION

As written by Bartholomew (1981), ‘it is only a slight overstatement to say that the most important attribute of an animal, both physically and ecologically, is its size’. This is because body size (measured either as length or as mass) and form (i.e., body shape) fundamentally define the range of ecological niches an animal can occupy (Blanckenhorn, 2000; Dalponti et al., 2018; Schmidt‐Nielsen, 1984). Reconstructing the body size and form of extinct animals can thus help us understand their palaeobiology and palaeoecology (O'Keefe et al., 2011; Sander et al., 2021; Sternes et al., 2024). This can include basic biological information like physiology, dietary, locomotor, spatial and reproductive biology (Ferrón et al., 2018; Finnegan & Droser, 2008; Grogan & Lund, 2011; Pyenson & Vermeij, 2016) or broader evolutionary and ecological patterns like predator–prey relationships, past ecosystem dynamics and mass extinction selectivity (Farlow & Planka, 2002; Finnegan & Droser, 2008; Grogan & Lund, 2011; Monarrez et al., 2021; Morgan et al., 1995; Payne & Heim, 2020; Pyenson & Vermeij, 2016; Sallan & Galimberti, 2015). However, estimating the body size and form in extinct species is often challenging. Many taxa of interest are known only from a handful of anatomically incomplete specimens, which may exhibit highly idiosyncratic body plans (Bianucci et al., 2023) or leave little direct evidence (pertaining from the fossil record) of how morphology should scale with body size.

**FIGURE 1:**
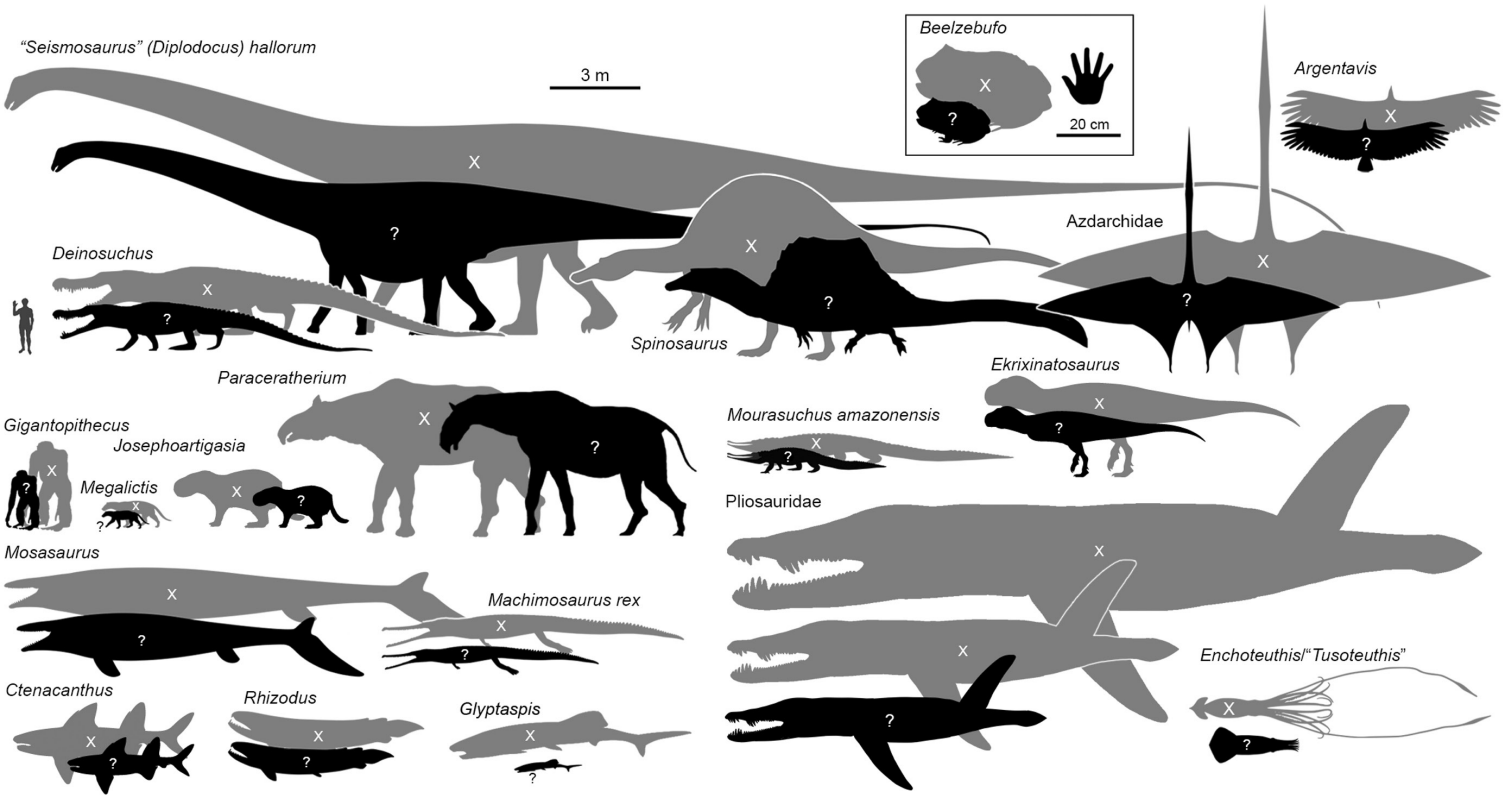
Examples of reconstructions of extinct megafauna, showing early estimates of body size/form now thought to be inaccurate (grey silhouettes), and more recent estimates, the validity of which remain uncertain (black silhouettes). Original work and sources of these reconstructions are listed in the Data S1, and we do not necessarily endorse any particular reconstruction over others. Credit for the original silhouettes used to produce this figure are as follows: Guillame Dera, CC0 1.0 (*Enchoteuthis* before), Tyler Greenfield with input from Dirk Fuchs, CC‐BY 3.0 (*Enchoteuthis* after), Scott Hartman, CC‐BY 3.0 (*Seismosaurus*), JF Studios, CC0 (*Pliosauridae*), T. K. Robinson CC‐BY 3.0 (*Mosasaurus*), Nobu Tamura (*Josephoartigasia* body), Gustavo Lecuona (*Josephoartigasia* head), both CC‐BY 3.0, Andrews (1985) (*Rhizodus*), Pimiento et al. (2024) (*Glyptapsis*), Nobu Tamura (modified by T. Michael Keesey; *Beelzebufo*), Granger et al. (1936) (*Paraceratherium* before), Larramendi (2015) (*Paraceratherium* after), Russell Engelman, modified from Hodnett et al., 2021, CC‐BY 4.0 (*Ctenacanthus*), Scott Hartman, CC‐BY 3.0 (*Machimosaurus*), Mark P. Witton and Darren Naish, CC‐BY 3.0 (*Quetzalcoatlus*), Dal Sasso et al. (2005) (*Spinosaurus* before), Tasman Dixson, CC0 (*Spinosaurus* after), Dan Niel, CC0 (*Argentavis*), T. Michael Keesey, CC0 (*Gigantopithecus*), Anton et al. (2004) (*Megalictis*). Jagged Fang Designs, CC0 (*Ekrixinatosaurus*), Ferran Sayol, CC0 (*Mourasuchus*, body) and Langston (1965, *Mourasuchus*, head).

The body size and shape of various iconic, large‐bodied extinct taxa have been estimated by combining fossil data with physical measurements taken from extant or extinct proxies (Table 1) presumed to be closely related to the taxon in question (Millien, 2008; Millien & Bovy, 2010) and/or to display significant ecological and morphological similarities (Ferrón et al., 2017). This ‘extant scaling’ approach typically relies on regression equations generated from modern species or well‐preserved fossil taxa, creating allometric scaling relationships that are then applied to homologous (or superficially similar) features on the extinct study species. Many studies do not even use regression equations at all, instead selecting a single proxy taxon (either a single specimen or a representative reconstruction of a single taxon) and then scaling the proxy up or down to the size of the incomplete fossil material using the proportional size of an anatomical measurement assumed to scale isometrically (Lingham‐Soliar, 1995; Lomax et al., 2024; Molnar, 2004; Savage, 1973). Importantly, the term proxy refers to both taxon or taxa, and the specific anatomical or morphometric character that is typically assumed to be homologous between the proxy taxon and the taxon of interest. No method of reconstructing body form or size in extinct taxa is flawless (Nelson et al., 2023), and many recent studies have proven highly controversial, prompting the publication of rebuttals and revisions (Engelman, 2022b, 2023a; Grillo & Delcourt, 2017; Millien, 2008; Millien & Bovy, 2010; Motani & Pyenson, 2024; Romano & Manucci, 2021; Sternes et al., 2023, 2024). Differences in estimated body size across these studies are not minor (Figure 1), with revised size estimates often being half or less than their originally proposed value (e.g., Cidade et al., 2019; see Table S2). These situations feed into a broader problem regarding scepticism and mistrust towards scientists – the ‘death of expertise’ (Nichols, 2017). The frequency and magnitude with which size estimates for megafauna need to be revisited poses difficulty for scientists in maintaining public trust and confidence, potentially bleeding into public opinion on other matters of importance including climate change and conservation issues.

**TABLE 1:** Taxa of unusually large size (1) which have been the subject of attempts to estimate their body size and/or form from fragmentary material, (2) which have received a significant amount of research or popular attention due to their unusual size, or (3) for which previous estimates of size/shape have been controversial (denoted with an asterisk).

Higher taxa	Example taxa
Cephalopoda: Nautiloidea	Endoceras/Cameroceras*, *Rayonnoceras*
Cephalopoda: Belemnitida	*Megateuthis*
Cephalopoda: Coleoidea	*Enchoteuthis* (‘*Tusoteuthis*’)*
Arthropoda: Radiodonta	Anomalocaris*
Arthropoda: Eurypterida	Jaekelopterus*, *Pterygotus*
Arthropoda: Myriapoda	*Arthropleura*
Arthropoda: Insecta	Meganeura*, Meganeuropsis*
Placodermi: Arthrodira	Dunkleosteus*, Titanichthys*, Glyptaspis*
Chondrichthyes: Eugeneodontiformes	*Helicoprion*, *Edestus*, Parahelicoprion*
Chondrichthyes: Orodontiformes	Orodus*
Chondrichthyes: Ctenacanthiformes	Ctenacanthus*, *Saivodus*, the ‘Texas Supershark’
Chondrichthyes: Lamniformes	Megalodon (*Otodus megalodon*)*
Osteichthyes: Pachycormiformes	Leedsichthys*
Osteichthyes: Salmoniformes	*Oncorhynchus rastrosus*
Sarcopterygii	Rhizodus*, *Hyneria*, Mawsonia*
Temnospondyli	Prionosuchus*, *Eryops*, Mastodonsauridae, the ‘Precious of Lesotho’
Anura	Beelzebufo*
Squamata: terrestrial taxa	megalania (*Varanus priscus*)*, *Barbaturex*
Squamata: Serpentes	*Titanoboa*, *Vasuki*
Squamata: Mosasauridae	Mosasaurus*, *Tylosaurus*
Testudines	*Stupendemys*, *Caninemys*, *Peltocephalus maturin*, Meolaniidae, giant tortoises (Megalochelys atlas*)
Crocodyliformes: Thallatosuchia	Machimosaurus*, Metriorhynchidae*
Crocodyliformes: Notosuchia	*Barinasuchus*, Kaprosuchus*
Crocodyliformes: stem Neosuchia	Aegisuchus*, Sarcosuchus*
Crocodyliformes: Crocodylia	Deinosuchus*, Purussaurus*, Mourasuchus*
Ichthyopterygia	*Cymbospondylus*, Shonisaurus*, ‘*Shastasaurus*’ sikanniensis*, Ichthyotitan*, the ‘Aust Colossus’
Sauropterygia	Liopleurodon*, Kronosaurus* Pliosaurus*
Pterosauria	*Pteranodon*, Quetzalcoatlus*, Arambourgiana* Hatzegopteryx*
Dinosauria: flightless Avialae	Dinornithidae*, Aepyornithidae, Dromornithidae*, Gastornithidae*, Phorusrhacidae
Dinosauria: volant Avialae	Pelagornithidae*, Argentavis*
Dinosauria: Sphenisciformes	Anthropornis*, Pachydyptes*, *Kairuku*
Dinosauria: non‐avian Theropoda	Many, e.g., *Tyrannosaurus*, Spinosaurus*, several Abelisauridae (Abelisaurus*, Ekrixinatosaurus*)
Dinosauria: Sauropoda	Many, e.g., Dreadnoughtus*, ‘*Seismosaurus*’*, Futalognkosaurus*, Bruhathkayosaurus*, Maraapunisaurus*
Dinosauria: Ornithischia	Many, e.g., *Triceratops*, *Stegosaurus*
Synapsida: Dicynodontia	Lisowicia*
Mammalia: Dasyuromorphia	Thylacinus cynocephalus*, T. potens*
Mammalia: Diprotodontia	*Diprotodon*, Thylacoleo*, Procoptodon*
Mammalia: Proboscidea	Palaeoloxodon*, *Mammuthus*
Mammalia: Rhinoceratoidea	Paraceratherium*, Elasmotherium*
Mammalia: Cetacea	Perucetus*, Livyatan*
Mammalia: Hyaenodonta	Megistotherium*, Hyainailouros*, *Simbakubwa*
Mammalia: Carnivora	*Arctotherium*, *Arctodus*, *Smilodon*, Megalictis*
Mammalia: Rodentia	Josephoartigasia*, Phoberomys*, Telicomys*, *Casteroides*
Mammalia: Primates	Gigantopithecus*

*Note*: Key references for each taxon are listed in Table S1, where further details regarding body size estimates in these taxa are also provided. Asterisks (*) denote taxa in which estimates of body size/form have proven controversial.

Despite this, palaeobiologists generally agree that some information from extant or extinct proxies is necessary to estimate body form and/or size in extinct animals. Even multivariate or volumetric models, which some authors regard as more accurate than simple linear regressions (Bates et al., 2015; Brassey, 2016; Romano & Manucci, 2021), still rely on data and underlying assumptions from modern taxa such as soft‐tissue distribution and density (Bates et al., 2009; Bianucci et al., 2023; Campione & Evans, 2020; Motani & Pyenson, 2024). Volumetric models also require silhouettes or skeletal reconstructions as input data (Brassey, 2016; Henderson, 2010; Motani, 2001) – outside of rare cases in which the entire skeleton is known – relying on pre‐existing estimates of body size (e.g., total length) and form. Optimisation of body size and form estimates thus depends on the selection of appropriate proxies and mathematical models while acknowledging the intrinsic limitations of estimations based on data from extant species.

**FIGURE 2:**
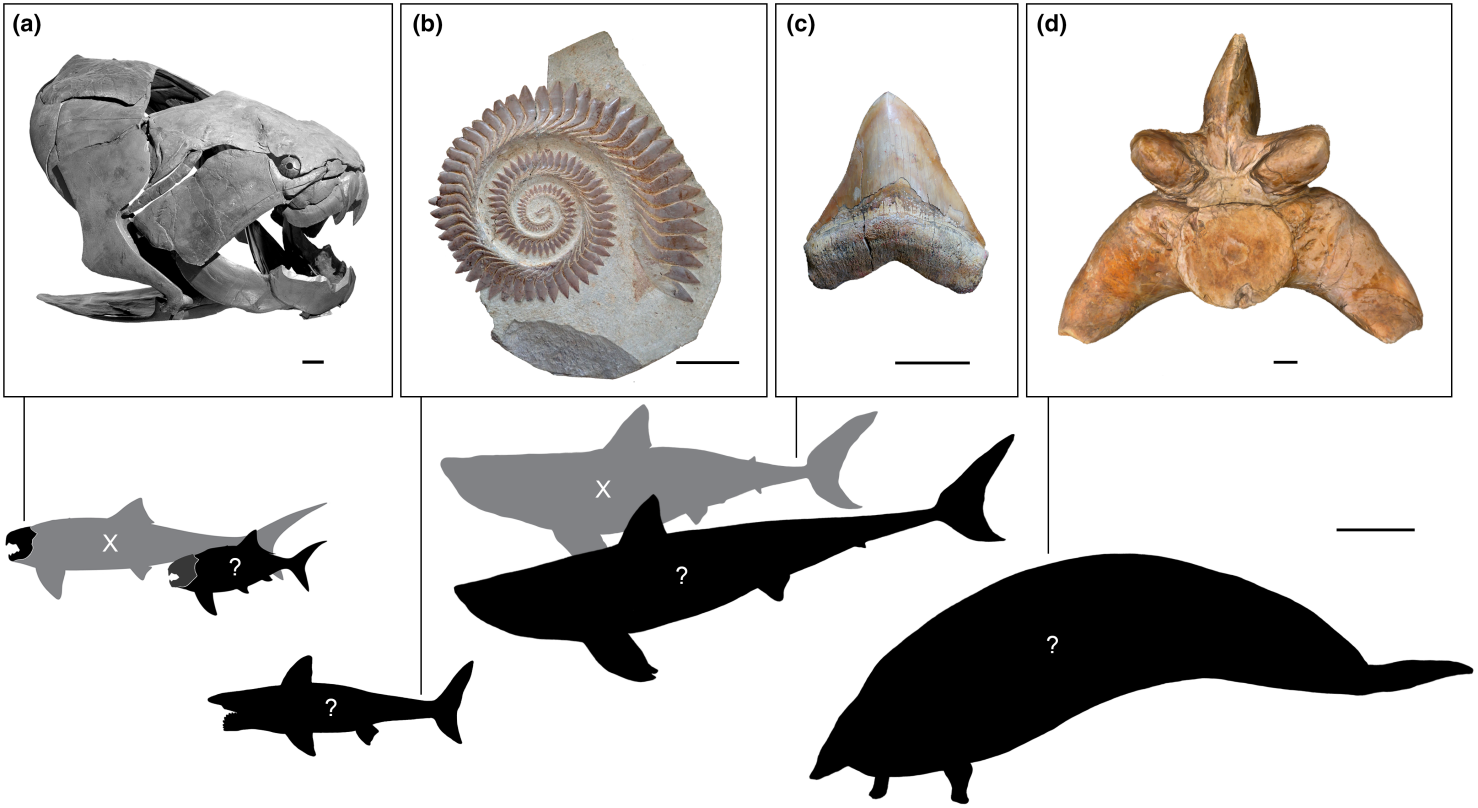
Fossil (or cast) material for each of the four case studies (skull and trunk armour from a cast of *Dunkleosteus terrelli*, (a); tooth whorl from *Helicoprion* spp, (b); tooth from *Otodus megalodon*, (c); and vertebra from *Perucetus colossus*, (d); respectively), along with silhouettes representing reconstructions of body size and form, where grey silhouettes are past reconstructions now thought to be inaccurate and black silhouettes are recent reconstructions, the validity of which remains uncertain. Scale bars = 5 cm for fossil/cast material and 2 m for silhouettes. Image credits for fossil/cast material are as follows: Archival Photograph GEO82014 of cast CMNH 5768, Field Museum of Natural History, Chicago, Illinois, USA (a), *Helicoprion bessonowi* Karpinsky, 1899, holotype specimen, F.N. Chernyshev Central Scientific Research Geological Prospecting Museum, St. Petersburg, Russia, 1/1865 (b), *Otodus megalodon* tooth MUSM 2093, Sacaco, Museo de Historia Natural (UNMSM) Peru (c), *Perucetus colossus* holotype MUSM 3248, Museo de Historia Natural (UNMSM) Peru. Silhouette images were modified from image used with permission by Yang Song (grey, a) created by Russell Engelman (black, a), used with permission from William Snyder under a CC BY‐SA 4.0 licence (b), or modified from Sternes et al. (2024) (c) and Bianucci et al. (2023) (d).

In this review, we consider four prominent case studies of body size estimation in extinct organisms, focusing on marine megafauna (Figure 2). These case studies were chosen as they represent a range of body sizes, ecomorphological niches and geological time intervals. Moreover, the quality and extent of the fossil record of each of these taxa differ markedly, as do the approaches used to estimate their body size and form. *Otodus megalodon* is largely known only from isolated teeth and an incomplete vertebral column, and despite the existence of modern relatives (lamniform sharks), there is debate surrounding the validity of these relatives as proxies (Sternes et al., 2023, 2024). *Perucetus colossus* also has modern relatives (whales) but is known only from vertebrae, ribs and the incomplete pelvis of a single individual (Bianucci et al., 2023). By contrast, *Dunkleosteus terrelli* is a Palaeozoic fish that is almost exclusively known from dermal armour that covered the head and anterior trunk and has no modern relatives (Engelman, 2023b, 2024), and *Helicoprion* spp., another Palaeozoic fish, is even more challenging to reconstruct; not only does it have no modern relatives whatsoever but is known only from isolated tooth whorls of contentious position and function (Karpinsky, 1899). Thus, each of these taxa represents a unique ‘enigma’ in the palaeontological literature, where body size has been estimated using necessarily different approaches based on the available data. It is important to note while all of these case studies are marine megafauna, the concepts and principles we discuss throughout the perspective are applicable to all extinct animals (Figure 1; Table 1; Data S1). We explore how and on what data these size estimates were produced and the resulting controversy in the literature. Subsequently, we use these case studies to synthesise broad thematic limitations in palaeobiology and the use of extant and extinct proxy taxa to estimate body size and form in extinct animals, including mathematical, phylogenetic and social issues.

## 2 CASE STUDIES

### 2.1 *Dunkleosteus terrelli* (Placodermi: Arthrodira)

*Dunkleosteus terrelli* is a large late Devonian arthrodire placoderm, known for its bony armour and guillotine‐like jaws. However, outside of its bony head and trunk armour (Figure 2a), the rest of its body was composed of perichondrally ossified cartilage. This material rarely preserves, complicating estimates of body size and morphology (Carr, 2010; Ferrón et al., 2017). Additionally, *Dunkleosteus* has no close living relatives that can be used to interpret its anatomy. Complete remains are known for some smaller arthrodires (Jobbins et al., 2022; Miles & Westoll, 1968), but these taxa likely differed from *D. terrelli* in ecological niche and body shape (Ferrón et al., 2017).

*Dunkleosteus* has been traditionally cited as reaching 5–10 m in total length (TL). However, these are generally speculative estimates that omit key methodological details as to how they were produced, including what taxa or anatomical elements were used as proxies, what specimens of *Dunkleosteus* were examined and the measurements used to produce these estimates (see Engelman, 2023b; Ferrón et al., 2017 for details), making these estimates non‐reproducible and highly questionable. One exception was Ferrón et al. (2017), which calculated the TL of *D. terrelli* using the upper jaw perimeter of select large, nektonic extant sharks to estimate its caudal fin shape, as their method required body size as an independent predictor variable. This study assumed mouth and body size likely correlate in fishes because predator size is correlated with prey size, which in turn correlated with gape, meaning mouth size and body size may be indirectly linked (Ferrón et al., 2017). However, this study did not test if sharks and arthrodires showed comparable mouth proportions or cross‐examine the accuracy of the resulting body length estimates (Engelman, 2023b; Ferrón et al., 2017). Testing this method on complete arthrodires finds arthrodires have proportionally larger mouths than sharks, producing overestimates of TL 2–2.5 times the actual value (Engelman, 2023a, 2023b). Traditionally cited lengths for *Dunkleosteus* also require a hyper‐elongate trunk, with a head of only ~8% TL (see Figure 1), and head–body proportions that are more extreme than even eels. Not only are these proportions implausible, but they are also unlike complete arthrodires or non‐anguilliform fishes (in which head length is generally ~17–30% TL; Engelman, 2023b).

Because of the inability of mouth dimensions to reliably estimate the size of arthrodires, Engelman (2023a) attempted to estimate the size of *Dunkleosteus* based on the combined length of the neurocranial and branchial regions of the head (i.e., head length minus preorbital length; Engelman, 2023a), reasoning that the scaling of these areas with body size was likely strongly constrained due to their function. This study (Engelman, 2023a) selected a variable based on homologous landmarks between *Dunkleosteus* and a wide range of extant proxies. It also tested the initial assumption of correlation to ensure that scaling patterns were consistent across taxa. Furthermore, it used complete arthrodires as case studies to ensure that the method accurately predicted body size in this group, suggesting that this method should produce reliable sizes for *Dunkleosteus* in the absence of complete specimens. This resulted in lengths of only 3.4–4.1 m for large *D. terrelli* individuals, with even the upper ends of the margin of error barely overlapping with the smallest lengths in previous studies (Engelman, 2023a). Independent tests by scaling from other arthrodires produced similar results, and the reconstructed body shape for *Dunkleosteus* better agrees with the comparative anatomy of other arthrodires (e.g., in fin location/size relative to TL and trunk armour dimensions; Engelman, 2023a, 2024). Arthrodires, actinopterygians and chondrichthyans showed significant, clade‐specific differences in overall body form, with complete arthrodires exhibiting a much stockier body plan compared to chondrichthyans (Engelman, 2023a).

### 2.2 *Helicoprion* spp.: (Chondrichthyes: Eugeneodontidiformes)

*Helicoprion*, found globally in Permian deposits, is arguably the most widely recognised Palaeozoic chondrichthyan. Its notoriety stems from the unique nature of its spiraliform tooth whorls (Figure 2b), which constitute practically the only known remains of the taxon. The lack of any reasonable extant or extinct proxies to the tooth whorls of *Helicoprion* has presented significant difficulties in inferring its body size and form. For some time after the description of *Helicoprion* by Karpinsky (1899), there was no agreement as to the position of the tooth whorl on the body of the animal or even its nature (e.g., dermal spine or oral teeth), making inference of body size or form impossible. Although Karpinsky (1899) considered other interpretations, he favoured a location of the tooth whorl mostly external to the mouth and extended from the upper jaw. However, based on examination of IMNH (Idaho Museum of Natural History) 37899, a unique specimen of *H. davisii* from the Permian Phosphoria Formation of Idaho, USA, with remains of cranial cartilage preserved, Bendix‐Almgreen (1966) concluded the tooth whorl was located at the symphysis of the lower jaw, internal to the buccal cavity, and that no similar tooth whorl was in the upper jaw.

Subsequently, Lebedev (2009) made the first quantitative reconstruction of the body size and shape of *Helicoprion*, using (1) extant odontocetes (toothed whales) and (2) extinct eugeneodonts known from nearly complete remains (*Caseodus*, *Romerodus*, and *Fadenia*) as proxies. The first proxy was based on a presumed similarity in size, diet (fish and cephalopods) and habitat (pelagic) and the second on phylogenetic proximity. Lebedev's reconstruction featured an elongated lower jaw, with the tooth whorl placed near the distal end, and conventional fusiform fish body proportions. Based on the eugeneodont proxies, Lebedev inferred a head length of 2.5–4.0 times the tooth whorl diameter. An assumption of a ratio of body length to head length of 5.0 (conventional fish body proportions) yields a body length (TL) of 12.5–20 times the tooth‐whorl diameter. For a tooth whorl with a diameter of 0.56 m (the largest one listed in Tapanila & Pruitt, 2013), this corresponds to a range of 7.0–11.2 m TL. Later examination by Tapanila et al. (2013) of IMNH 37899 by X‐CT (X‐ray computed tomography) showed that *Helicoprion*'s lower jaw was shorter than Lebedev envisioned and that the ratio of the head length to the tooth‐whorl diameter was approximately 2.5 (Tapanila et al., 2020, figure 3), resulting in an inferred body length of approximately 7.0 m TL.

### 2.3 *Otodus megalodon* (Chondrichthyes: Lamniformes)

The megatooth shark, *Otodus megalodon*, is an iconic extinct elasmobranch represented primarily by gigantic teeth (Figure 2c) commonly found from the mid‐Miocene to the Early Pliocene nearly worldwide (Boessenecker et al., 2019; Cappetta, 2012; Cooper et al., 2020, 2022; Gottfried et al., 1996; Sternes et al., 2023). The biology of *O. megalodon* has remained difficult to decipher. Although some vertebral remains, placoid scales, and fragments of tessellated cartilage of *O. megalodon* have been reported (Bendix‐Almgreen, 1983; Leriche, 1926; Shimada et al., 2023), the limited fossil record has hampered attempts to estimate its true size and to reconstruct body form. When such attempts are made, the extant white shark (*Carcharodon carcharias*) has been assumed to be a logical ecological proxy due to its similar tooth form and trophic ecology to *O. megalodon* (Collareta et al., 2017; Gottfried et al., 1996).

The most common size estimation method for *O. megalodon* has been extrapolation from scaling relationships between dental measurements and total body lengths in *C. carcharias* (Perez et al., 2021; Randall, 1973; Shimada, 2002, 2019). These studies generally suggest a maximum length of 15–20 m for *O. megalodon*. Alternatively, the allometric relationship of vertebral diameters in extant *C. carcharias* (Gottfried et al., 1996) has been used to extrapolate body size based on incremental growth bands preserved in a set of vertebral specimens of *O. megalodon*, producing a length estimate at birth of 2 m (Shimada et al., 2021).

Likewise, the body form of *O. megalodon* has traditionally been modelled after extant *C. carcharias* (Bendix‐Almgreen, 1982; Cooper et al., 2020, 2022; Gottfried et al., 1996). This seemed logical especially in earlier studies (Applegate & Espinosa‐Arrubarrena, 1996; Gottfried et al., 1996), due to the similarly serrated dental morphology, the fossil shark was placed in the genus *Carcharodon* (Lamnidae) with the interpretation that ‘*C*.’ *megalodon* was the direct ancestor of extant *C. carcharias*. As of now, the fossil species is considered to belong to *Otodus* (Otodontidae) rather than *Carcharodon* (Shimada et al., 2017). Despite *C. carcharias* and *O. megalodon* likely not having any direct phylogenetic link within Lamniformes (Shimada, 2022; Shimada et al., 2017; Sternes et al., 2023, 2024), the use of *C. carcharias* as a proxy has continued based on the assumption that *O. megalodon* was an active, regionally endothermic (Ferrón, 2017) predator like the extant lamnids (Cooper et al., 2020, 2022).

Geochemical evidence has subsequently confirmed that *O. megalodon* was a ‘warm‐blooded’ shark (Griffiths et al., 2023) occupying a high trophic position (Kast et al., 2022; McCormack et al., 2022). However, the assumption that *O. megalodon* must have resembled extant *C. carcharias* has been called into question. Support for the relationship between body form and thermophysiology in sharks has weakened (Dolton, Jackson, et al., 2023; Dolton, Snelling, et al., 2023; Sternes et al., 2023), and the absence of keels on its scales suggests that *O. megalodon* was likely not a fast swimmer like extant lamnids. Additionally, the total combined length of a partial vertebral column of *O. megalodon* from the Miocene of Belgium, previously estimated to be from a 9.2‐m‐TL individual based on comparisons of vertebral diameters in extant *C. carcharias* (Gottfried et al., 1996), was measured to be 11.1 m (Cooper et al., 2022). This discrepancy – that is, the actual measured length of the incomplete vertebral column (not considering the head or caudal fin) being substantially longer than the TL estimate for this specimen based on the extant *C. carcharias* (9.2 m TL) – strongly indicates that *O. megalodon* was not merely a larger version of extant *C. carcharias* but rather a proportionately slender, more elongate shark (Sternes et al., 2024). The exact body morphology and maximum TL of *O. megalodon* remain uncertain, but the implications are significant. Not only may the use of extant *C. carcharias* not be appropriate for deciphering the body form and body size of *O. megalodon*, but the observation also strongly indicates that all previous TL estimates based on dental proportions of extant *C. carcharias*, which concluded a maximum TL of at least 15 m for the species (Shimada, 2019), may be underestimates.

### 2.4 *Perucetus colossus* (Mammalia: Artiodactyla)

*Perucetus colossus* is a recently described, large‐bodied archaeocete (stem cetacean) from the late middle Eocene of Peru (Bianucci et al., 2023). This taxon is only represented by the holotype, consisting of 13 vertebrae (Figure 2d), 4 ribs, and the right innominate. *Perucetus* is considered a member of Basilosauridae, the derived group of fully aquatic archaeocetes that may represent the sister group of crown Cetacea. *Perucetus* exhibits extreme pachyostosis and osteosclerosis, traits generally associated with shallow diving in modern aquatic mammals (Buffrénil et al., 2010).

Despite belonging to a clade with modern representatives (crown Cetacea), there are no close extant analogues for the large, serpentiform body plan of basilosaurids. Thus, to reconstruct the body size of *Perucetus*, Bianucci et al. (2023) utilised multiple proxies including sirenians and neocetes. Bianucci et al. (2023) first scaled up and dilated a digital 3D model of the skeleton of the basilosaurid *Cynthiacetus peruvianus* such that the volume of its bones equalled that of the corresponding skeletal elements of *P. colossus* (producing multiple models to account for the uncertain position of the latter's vertebrae and variable vertebral count among basilosaurids), yielding estimates of skeletal length and volume ranging between ~17–20 m and 2.9–4.1 m^3^, respectively. Body mass values were then calculated for the *P. colossus* holotype based on the range of skeletal fraction (SF) observed in extant marine mammals, yielding minimum and maximum estimates of 85 t (assuming the mean SF of large *Trichechus manatus* and the minimum skeletal volume) and 340 t (assuming the SF of *Mesoplodon europaeus* and the maximum skeletal volume), respectively (Bianucci et al., 2023).

Subsequently, Motani and Pyenson (2024) raised several criticisms of Bianucci et al. (2023)'s analysis. For example, Motani and Pyenson (2024) suggested that it would be impossible to fit 180–340 t of biomass (corresponding to the upper portion of Bianucci et al. (2023)'s range of body mass estimates) into the volume of a 20 m whale, with their volumetric methods instead producing estimates of 60–114 t using volumetric methods. They also questioned Bianucci et al. (2023)'s use of a simple SF ratio as opposed to an allometric regression equation, arguing that across marine and terrestrial mammals (including both neocetes and sirenians), SF appears to scale with positive allometry rather than isometry. Using regression equations, Motani and Pyenson (2024) produced estimates of 135–193 t assuming a neocete‐like SF and 40.0–54.6 t using a sirenian‐like SF (Motani & Pyenson, 2024). Nevertheless, this revised body mass estimate is not free of limitations either, namely, reliance on a model created from a 2D palaeoartistic reconstruction that was not depicted in direct lateral view. This method is by no means intrinsically flawed but is heavily dependent on approximation of three‐dimensional body proportions. Alternatively, a silhouette could have been produced from the modified *Cynthiacetus* model presented by Bianucci et al. (2023), which could have yielded a different result.

## 3 WHY ESTIMATING THE BODY SIZE AND FORM OF EXTINCT ANIMALS MATTERS

The body size and form estimates presented in each of the case studies discussed here have significant consequences for our understanding of vertebrate macroevolution, beyond the palaeoecology of the individual taxa themselves. In the case of *Dunkleosteus*, the Devonian was long thought to be a period of explosive body size expansion in vertebrates, in part, because of the abrupt appearance of large placoderms (including *Dunkleosteus*; Dahl et al., 2010; Sallan & Galimberti, 2015). However, the same methodology that downsized this iconic taxon (Engelman, 2023a) also produces lengths <5 m for other large Devonian placoderms like *Gorgonichthys* and *Titanichthys*. Other Devonian and early Carboniferous vertebrates like ctenacanths and sarcopterygians appear to have reached similar maximal sizes (Engelman, 2023a; Jeffery, 1998; Young et al., 2013), implying that vertebrates likely did not reach the size of modern marine megafauna (i.e., white sharks, basking sharks, whale sharks and cetaceans) until well into the Carboniferous (Engelman, 2023a), much later than traditionally thought. This is of great importance for our understanding of gigantism and body size evolution in vertebrates, as well as the structure and function of Palaeozoic ecosystems.

Similar insights into macroevolutionary trends can be deciphered from *Perucetus*, which indicates at least two distinct periods of cetacean gigantism (Bianucci et al., 2023). Despite the controversy over its weight, both Bianucci et al. (2023) and Motani and Pyenson (2024) agree that *Perucetus* was very large, comparable in size to modern physeterids and rorquals and at least an order of magnitude heavier than the next largest Palaeogene whale (*Basilosaurus*; see Motani & Pyenson, 2024: p. 18). Crucially, Eocene basilosaurids evolved giant body sizes in coastal settings with high seafloor productivity and global cooling rather than in the pelagic, open‐marine realm like their modern mysticete relatives (Bianucci et al., 2019). Similarly, along with the rise of a potential ecological competitor (white shark, *Carcharodon carcharias*: Boessenecker et al., 2019; McCormack et al., 2022), late Neogene global cooling and restructuring of ocean circulation coincides with the demise of *O. megalodon*, which may have been exacerbated by its large size (Condamine et al., 2019).

Body size estimates of extinct taxa also contribute to our understanding of trophic dynamics within past ecosystems, as body size and form are critical in determining the range of predator and prey species with which a species can interact. During the Permo‐Carboniferous, most large marine vertebrates were chondrichthyans (Schnetz et al., 2024), many of which (e.g., Petalodontiformes) cannot easily be compared to modern chondrichthyans in terms of their dental anatomy or body shape (Ginter et al., 2010). This included various whorl‐toothed eugeneodonts, including *Helicoprion*, *Edestus giganteus* (Newberry, 1889) and *Karpinskiprion ivanovi* (Lebedev et al., 2022), which were among the largest organisms in their respective ecosystems. Without reliable estimates of body size in *Helicoprion* and related eugeneodonts, it is difficult to reconstruct the structure of Permo‐Carboniferous marine food webs or determine if patterns of body size evolution correlate with other events such as the Carboniferous Rainforest Collapse (McGhee, 2018; Schnetz et al., 2024) or gigantism proposed for marine invertebrates (McGhee, 2018).

Several studies identifying likely spurious size estimates have also discussed their immediate downstream consequences on our understanding of evolutionary history (Engelman, 2023a, 2023b; Fortelius & Kappelman, 1993; Grilo & Delcourt, 2017; Romano & Manucci, 2021; Rovinsky et al., 2020). In particular, because many of these taxa exist at the extremes of the variation seen in nature, spurious size estimates have the potential to bias discussions about biomechanical and physiological limits of animal size (Witton & Habib, 2010) or patterns of body size evolution in evolutionary history (Engelman, 2023a, 2023b; Grillo & Delcourt, 2017; Romano & Manucci, 2021). One example of this is estimating the body mass of large Late Cretaceous azdarchid pterosaurs such as *Pterodon longiceps* and *Quetzalcoatlus northropi*. These are some of the largest known flying organisms and thus provide key information to discussions of possible biomechanical limits in powered flight. As noted by Witton and Habib (2010: p. 2) ‘[a]ccurately modelling the size of giant forms is essential to appreciating their flight ability as even relatively small over‐predictions of wingspans may translate to considerable over‐estimates of mass and subsequently inaccurate appreciation of flight performance’. Several biomechanical studies concluded these taxa were incapable of powered flight, at least in part on the basis of estimated body mass (Henderson, 2010; Sato et al., 2008). However, subsequent research has shown that these values are likely overestimates and/or the result of incorrect modelling of body form in a volumetric model (Witton, 2008; Witton & Habib, 2010). Alternatively, work into the launch mechanics of *Q. northropi* assumed flight capability under a bipedal, bird‐like launching model (Chatterjee & Templin, 2004), resulting in an upper mass estimate of 75 kg, that would require the animal to be nearly 80% air by volume (Witton, 2008). Had extreme size estimates for these taxa been upheld by subsequent studies, it would have enormous consequences for our understanding of flight biomechanics and macroevolutionary transitions in flight capability in all volant taxa.

The issue in such cases is not that size estimates sometimes have to be revised. This is generally a natural consequence of working with fragmentary taxa often much larger than potential complete anatomical proxies (see below). The issue is that variation in size estimates between studies is massive, with estimates in one study frequently half (or twice) those presented in others (see Data S1), and these differences seem to be driven by methodological problems and data practices rather than true unknowns in estimating body size and/or form. This results in a potentially major and pervasive source of error in palaeoecological and evolutionary studies given that many rely to some extent on body size/form estimates of extinct taxa. Furthermore, because the body size of extinct organisms is a quality often immediately visible to the public, extremely large swings in size estimates reduce public confidence in the ability of palaeontologists to speak authoritatively about extinct life. A potential risk or consequence is that this phenomenon may lead to public disillusionment with palaeontology and an erroneous or unfounded belief that palaeontologists deliberately exaggerate the size of their subjects for prestige in response to high‐profile studies revising size estimates.

## 4 LIMITATIONS

### 4.1 Modelling and extrapolation

The most fundamental requirement for predicting body size and/or form of an extinct species is that the proxy accurately predicts body size in the first place (Bates et al., 2009). This might seem trivial, but different physical features exhibit diverse scaling relationships across taxa, and the selection of specific anatomical units to model body size requires careful examination and abundant data (e.g., Field et al., 2013). Nelson et al. (2023) found that limb bone cross‐sectional dimensions (diameters and circumferences), long considered the strongest predictors of body size among terrestrial vertebrates (Anderson et al., 1985; Campione & Evans, 2012; Ruff, 1990), had significant, non‐random bias independent of phylogeny but seemingly correlated with body robustness – a factor that is difficult to control for mathematically without circular logic. Unfortunately, existing studies often do not explore possible sources of allometric bias or uncertainty when selecting size proxies, but assume a close and consistent relationship with body size between the proxy and focal taxa a priori (Cooper et al., 2020, 2022; Ferrón et al., 2017; Gottfried et al., 1996). This issue is exemplified by initial body size estimates for *Dunkleosteus*, which were based on scaling relationships of the upper jaw in extant sharks (Ferrón et al., 2017) despite a lack of evidence for similar jaw proportions between arthrodires and sharks (Engelman, 2023b). While post hoc analysis of arthrodire cranial scaling improved size estimates for *Dunkleosteus* (Engelman, 2023a, 2023b), it is extremely challenging if not impossible to verify the validity of proposed scaling trends in other taxa with few or no living (or well‐preserved fossil) relatives, such as *Helicoprion*. Whenever possible, proposed scaling relationships should be tested empirically using close relatives of the focal taxon to ensure model reliability (Engelman, 2023b) or cross‐testing size/form estimates using multiple proxies and/or scaling methods (Bianucci et al., 2023; Motani & Pyenson, 2024). Where this is not possible, we urge scepticism of resulting body size and form estimates.

Another common issue is extrapolation error. When using regression equations to estimate body size, imprecision typically increases as the size of anatomical elements from the focal taxon extends beyond that of the training data (Bates et al., 2009; Engelman, 2023a; Schmidt‐Nielsen, 1984). This is a particular problem if taxa of interest to palaeontologists are significantly larger or smaller than their nearest extant proxies or occupy size ranges where only a limited number of similar‐sized modern proxies exist (i.e., megafauna). This can be mitigated if taxa in the training data span a wide range of body sizes (Campione, 2017) but such wide samples are often not available. Perhaps, the best example of the effects of extrapolation error on body mass estimates can be seen in the extinct rodent *Josephoartigasia* (Engelman, 2023a; Millien, 2008), although it also applies to other taxa, including *Perucetus* (Bianucci et al., 2023; Motani & Pyenson, 2024).

Many size estimates run into problems with the ever‐present spectre of positive or negative allometry (Schmidt‐Nielsen, 1984), either assuming their anatomical proxies scale isometrically with body size or calculating their estimates via simple scaling ratios with a proxy taxon (which implicitly assumes isometry). However, isometry is typically the exception, not the rule, among scaling relationships (Raup & Stanley, 1978: p. 61). Regression models tend to be more robust to this kind of bias due to their variable slope, whereas simple scaling ratios can be biased by even slight deviations from isometry or if the size proxy chosen does not show a strong correlation with body size (Grillo & Delcourt, 2017). As with extrapolation error, sampling a wide array of body sizes in the training dataset is one of the best ways to detect positive or negative allometry. Body size estimates should always be made based on regression equations or volumetric models whenever possible. Estimating body size via simple scaling ratios from one or a few proxy specimens or taxa should be considered as a last resort if appropriate regression models are not available, and the resulting size and/or form estimates considered very tentative until more robust estimates of size can be produced.

Other issues arise from the fact that most variables in allometric scaling relationships are logarithmically distributed and thus log‐transformed before analyses. It is often assumed that relationships between variables are completely linearised by log transformation (Engelman, 2022b; Schmidt‐Nielsen, 1984). However, this is not always the case, and further inspection has shown some biological relationships previously thought to be log‐linear may, in fact, scale log‐curvilinearly (Bertram & Biewener, 1990; Engelman, 2022a; Knell, 2009; Venditti et al., 2024) with their curvature implying treating these variables log‐linearly may overestimate body size. This is problematic as the biological significance of log‐curvilinear relationships is not well understood, nor do their mathematical constants have a ready explanation (Knell, 2009; Manger et al., 1999) in contrast to log‐linear models which follow a power law (Schmidt‐Nielsen, 1984). Until we have an improved understanding of non‐linear allometry, the validity of linear approximations to these allometric relationships cannot be known. For this reason, we suggest future studies at least consider the possibility of non‐linear allometry in their datasets and report suspected log‐curvilinear relationships if found.

Log‐transformation creates other issues in model evaluation and prediction. Most regression models measure the strength of correlations between variables using the coefficient of determination (*r* ^2^), but for log‐transformed models (especially ones intended for prediction), *r* ^2^ is actually a poor measure of relationship strength. Coefficients of determination tend to unilaterally increase as data spread increases, and log‐transformation exacerbates this problem because it compresses the scatter of data points around the regression line (Smith, 1984; Van Valkenburgh, 1990), inflating *r* ^2^ values. This means that even log‐scaled models with *r* ^2^ greater than 0.9 can have poor prediction accuracy in practice (Smith, 1984; Van Valkenburgh, 1990). For this reason, percent error (%PE) and percent standard error of the estimate (%SEE) are often preferred as measures of predictive accuracy because they directly measure the accuracy of the predicted values (Campione & Evans, 2020; Engelman, 2022a, 2023a; Van Valkenburgh, 1990).

Log‐transformed regression equations also tend to produce unreasonably large prediction intervals, often on the scale of orders of magnitude. This is because antilog transformation turns the normally distributed residuals of a log‐scaled regression equation into non‐normally distributed (leptokurtotic) residuals on an arithmetic scale (Bates et al., 2009; Bertram & Biewener, 1990; Engelman, 2023a) with extremely long ‘tails’ to the resulting distribution. This in turn results in large error bars and substantial uncertainty in body size estimates, often beyond what is morphologically plausible. Phylogenetic comparative least squares and volumetric estimation methods provide possible mitigation measures, although in both cases reduced error range comes at a trade‐off with prediction accuracy (Campione, 2017; Campione & Evans, 2012). While this uncertainty is unavoidable, it should be accounted for when making biological inferences about palaeoecology. All mathematical models have assumptions and limitations, the validity of which should ideally be carefully considered when selecting proxies from which to estimate body size and/or form in extinct taxa.

### 4.2 Incomplete specimens

In most cases, the body size and/or form of extinct animals must be estimated from extant proxies due to the lack of complete fossil specimens. At a basic level, it is impossible to confidently and accurately predict the body size or form of an extinct organism without complete specimens as researchers have no way of knowing the size or shape of missing elements without relying on inference from proxy taxa. This issue affects each of the four case studies discussed here, none of which are known from complete specimens (Bendix‐Almgreen, 1966; Bianucci et al., 2023; Engelman, 2022b; Sternes et al., 2024; Figure 2). Indeed, the fragmentary nature of remains used to reconstruct the body size and form of many extinct taxa can substantially increase error. Fragmentary remains are often first used to estimate the size of some larger or complete morphological structure (e.g., skull), which is in turn used to approximate total length. These cascading assumptions result in the propagation of error at each stage of reconstruction (Molnar & Vasconcellos, 2016), further complicating downstream ecological, evolutionary and biomechanical interpretations.

A lack of anatomically complete specimens also increases the likelihood of existing remains being misinterpreted as belonging to different species and/or parts of the body. Errors in body size and form estimation resulting from anatomical misinterpretation or misdiagnosis can be seen in *Helicoprion*, where the consensus position of the tooth whorl on the body has changed on multiple occasions (Bendix‐Almgreen, 1966; Karpinsky, 1899). Similarly, extreme modifications in the available vertebrae of *Perucetus* relative to other basilosaurids prevent a precise identification of their position within the vertebral column, which adds considerable uncertainty to body size estimations. For *Dunkleosteus*, lengths of 5–10 m were at least partly based on a priori assumptions of this taxon exhibiting a greatly shortened trunk armour compared to other arthrodires, which were never validated and indeed subsequent observations showed it was likely incorrect (Engelman, 2024). Unfortunately, the limitations associated with incomplete fossil specimens are difficult to overcome without new palaeontological evidence. Yet, this issue highlights that body size and/or form estimates are intrinsically uncertain if lacking adequate fossil material. Wherever possible, studies should take this into consideration and acknowledge the potential for new palaeontological interpretations of the fossil specimens upon which estimations are based.

### 4.3 Intraspecific variation

Another important consideration when selecting proxies for estimating body size and form in extinct taxa is intraspecific variation. Ontogeny and sexual dimorphism exert substantial influence on both body size and shape (Hone et al., 2016; Mallon, 2017; Motani et al., 2018; Paiva et al., 2022; Sanchez‐Villagra, 2010), and this needs to be taken into account when reconstructing extinct species. Some studies use regression equations derived from allometric patterns within a single species to produce their size estimates (e.g., *Crocodylus porosus* and *Gavialis gangeticus* in Sereno et al., 2001; *Physeter macrocephalus* in Lambert et al., 2010; and *Carcharodon carcharias* in Gottfried et al., 1996), and this raises concerns about conflating intraspecific patterns of allometry across the growth curve of a single species with true patterns of interspecific allometry in mature individuals (Paiva et al., 2022). Several studies on extinct megafauna have noted that even very large individuals ‘still appear to be growing at the time of death’ based on sutural fusion and bone microstructure (Buchy et al., 2003; Evans et al., 2014; Hone et al., 2016; Lomax et al., 2024), yet in many cases still appear to be sexually mature (Erickson et al., 2007; Lee & Werning, 2008). This has sometimes led to speculation that these organisms could reach still larger sizes unsampled by the fossil record, but an alternate needs to be considered that this pattern is a result of paedomorphosis or peramorphic hypermorphosis – i.e., delaying sutural closure and prolonging features allowing rapid growth well into adulthood, with growth slowing but not ceasing upon sexual maturity (Lee & Werning, 2008) and cessation only occurring with senescence – which has been proposed as the mechanism by which these animals achieved such spectacular sizes in the first place (Lee & Werning, 2008; Lomax et al., 2018). A good example of this are *Pliosauridae*, which rarely exhibit neurocentral fusion even as adults (Araújo & Smith, 2023; Knutsen et al., 2012; McHenry, 2009) – a feature otherwise commonly used as an indicator of osteological maturity in reptiles. This suggests that some of these supposedly somatically immature individuals could be close to typical adult size and that traditional markers of somatic maturity may be less informative for megafauna (Hone et al., 2016).

Where shape estimates come from a small number of remains (e.g., *Helicoprion*, *O. megalodon* and *Perucetus*), life stage and sex cannot be included as confounding variables in regression and volumetric models. It is practically impossible to know the full size range of an extinct species (Mallon & Hone, 2024; Sanchez‐Villagra, 2010), and we rarely have an adequate understanding of sexual dimorphism in these taxa. Thus, the few fossil samples that do exist are simply treated as standard for their species or population. Even in the rare cases that isolated fossil remains can be distinguished as either adult or juvenile, phenomena such as pathologic gigantism (e.g., Carboniferous cephalopods, Manger et al., 1999) and insular dwarfism (e.g., *Palaeoloxodon* and *Europasaurus*, Herridge & Lister, 2012; Sander et al., 2006) make this assumption questionable. Similarly, it must be questioned whether treating the maximum sizes reached by presumably exceptional individuals as representative of the species in palaeoecological studies is as informative as using the more modest average adult size (Mallon & Hone, 2024: p. 8). For example, Mallon and Hone (2024) speculated that a hypothetical 15‐m and 15‐t ‘world record’ *Tyrannosaurus rex* would be so slow and require so much food that it would have to rely on scavenging or shift prey focus to sympatric titanosaurs – neither of which would be realistic behaviour for the species. Issues of ontogeny and intraspecific variation will continue to be an issue regardless of the proxy taxon used but should accounted for when considering which individuals of this proxy are to be used for size and form reconstructions, particularly where volumetric approaches are used. Ideally, studies should conduct routine sensitivity tests considering multiple models that account for variation in both ontogenetic stage and sex. Most importantly, researchers should not assume that size or form reconstructions made from a handful of incomplete remains are representative of the full range of morphology seen in an extant species, or that these remains represent the ‘average’ morphology.

### 4.4 Phylogenetic placement

When selecting proxies for estimating body size in extinct taxa, consideration of the phylogenetic placement of both proxy and extinct focal taxon is vital. Where proxies are selected on the basis of phylogenetic similarity, it is imperative that the phylogenetic placement of both taxa is well resolved, which is frequently not the case. Issues of phylogenetic uncertainty are systemic in palaeobiology (Marjanović & Laurin, 2007; Reeder et al., 2015). This is exemplified by *O. megalodon*, where *C. carcharias* has generally been considered the best modern proxy despite the uncertain placement of *O. megalodon* within lamniform sharks (Sternes et al., 2023, 2024). At best, this phylogenetic uncertainty raises doubts about the validity of specific proxy taxa and may distort the results of phylogenetic comparative analyses which are themselves used to estimate body size in some studies (Diniz‐Filho & Nabout, 2009; Paiva et al., 2022; Symonds & Elgar, 2013). In extreme cases, phylogenetic uncertainty could cloud our understanding of homology between the anatomical units that are used to predict body size and form, making it impossible to clarify the validity of proposed proxies. Of course, proxies are not always selected on the basis of phylogeny. Studies may alternatively seek to use taxa assumed to be convergent in ecological habits or body form (Engelman, 2023b; Ferrón et al., 2017). However, this too is problematic given that there is no guarantee that phylogenetically disparate groups should display similar scaling relationships between anatomical features, regardless of the perceived degree of morphological convergence. For this reason, we favour the consideration of phylogeny when selecting appropriate proxy taxa but stress that for such an approach to be valid requires well‐resolved phylogenetic placement of both the proxy and the study taxon. Ultimately, estimates of body size and form made in this way must always be treated with caution given that perceived phylogenetic relationships between these taxa are intrinsically hypothetical in nature and subject to revision upon the inclusion of new data.

### 4.5 Social pressures

While most spurious size/form estimates are likely driven by some combination of the factors outlined above (and a general unawareness of biostatistical best practices), social pressures and the nature of research academia also have the potential to influence reconstructions of body size/form in extinct animals. Studies reporting spectacular sizes for organisms are often widely read and publicised, which can significantly elevate the work of early‐career researchers and translate to significant opportunities for funding and public interest. Some extinct species have gained considerable media attention as a direct result of their unusual size relative to modern animals (Ferreira et al., 2024; Head et al., 2009, 2013; Molnar, 2004; Rinderknecht & Blanco, 2008; Wroe et al., 2004) and might otherwise have failed to appear in high impact journals or receive widespread public attention if they were smaller. At the same time, while journals are often eager to publish on studies suggesting spectacular sizes, more modest, revised estimates are less likely to be considered publishable as they are unlikely to garner broader interest. There also seems to be a tendency of human nature to overestimate the size of megafauna unless quantitatively measured. This is well‐demonstrated by several studies on extant megafauna noting that even experienced field biologists tend to overestimate the size of their subjects (Greer, 1974; Molnar, 2004; Randall, 1973; Wood, 1976; Woodward et al., 1995). Other palaeontologists have made similar observations. As noted by Grillo and Delcourt (2017: p. 83) in their study of abelisaurid theropods, ‘the fact that most published BL [body length; = total lengths] are overestimates reinforces a statement made by Therrien and Henderson (2007) that the lack of complete skeletal remains in large theropods gives free course to imagination, that allow researchers to present new specimens as ‘the largest’, ‘the heaviest’, or other kind of similar adjectives’. Similarly, Fuchs et al. (2020: p. 42) noted previous estimates of size and form in their study organism (*Enchoteuthis*) seemed to be based on the ‘hope’ (wording theirs) of a more spectacular animal rather than any fossil evidence. While it is unlikely that these factors are acting in all or even most cases of controversial size/form estimates, the current landscape of academia does potentially encourage overly generous size estimates. Consequently, in our attempt to cover all potential influences on body size/form estimates, we would be remiss to not mention these social pressures as a potential bias.

Several factors may also make researchers reluctant to publish modest size estimates of extinct taxa. Researchers may be reluctant to downsize spectacular charismatic megafauna for fear that it will reduce public interest in their research area or burn bridges in the academic community, which could have downstream consequences for collaborations, funding acquisition or even the outcome of peer review. They may also fear museums may restrict access to specimens or otherwise respond poorly to research downsizing their flagship taxon. Furthermore, one must be aware of backlash from the ever‐growing fan communities of prehistoric organisms (such as *Dunkleosteus*, *O. megalodon* and theropod dinosaurs) on the internet, who may feel strongly about the perceived appearance of their favourite organisms. None of these concerns are hypotheticals, and all have happened at one point or another to many palaeobiologists who study well‐known, iconic fossil taxa, including some of those mentioned in the present study. All studies should be judged on their scientific merit through debate and discourse, through which progressive improvements to our understanding of extinct animals can be gained.

## 5 CONCLUSIONS

All body size and shape estimates of extinct species rely to some extent on extinct or extant proxies. These estimates can provide important ecological and evolutionary information and will continue to do so in the future. However, several important limitations must be considered when using such an approach. The utility and validity of a given proxy depends not only on perceived morphological or phylogenetic relationships but also on the quality and quantity of the fossil record, palaeontologists' interpretation of their examined material, our understanding of ontogeny and sexual dimorphism, and the degree of phylogenetic uncertainty involved. At a more fundamental level, the chosen proxy species may influence the validity of the mathematical modelling approaches chosen. We argue that taking precautionary measures to address these factors is of paramount importance when determining which proxy taxa upon which size and form reconstructions of extinct taxa will be based and should be treated as necessary. Many of these uncertainties are unavoidable when dealing with fragmentary extinct taxa. However, wherever possible, studies should explicitly reference these limitations, improving the robustness of the ecological and evolutionary inferences that can be drawn.

## AUTHOR CONTRIBUTIONS

**Joel H. Gayford:** Conceptualization (lead); writing – original draft (lead); writing – review and editing (lead). **Russell K. Engelman:** Conceptualization (supporting); writing – original draft (supporting); writing – review and editing (supporting). **Phillip C. Sternes:** Conceptualization (supporting); writing – original draft (supporting); writing – review and editing (supporting). **Wayne M. Itano:** Writing – original draft (supporting); writing – review and editing (supporting). **Mohamad Bazzi:** Writing – original draft (supporting); writing – review and editing (supporting). **Alberto Collareta:** Writing – original draft (supporting); writing – review and editing (supporting). **Rodolfo Salas‐Gismondi:** Writing – original draft (supporting); writing – review and editing (supporting). **Kenshu Shimada:** Conceptualization (supporting); writing – original draft (supporting); writing – review and editing (supporting).

## FUNDING INFORMATION

The research of AC is supported by a grant from the Italian Ministero dell'Università e della Ricerca (PRIN Project 2022MAM9ZB).

## CONFLICT OF INTEREST STATEMENT

The authors declare that the research was conducted in the absence of any commercial or financial relationships that could be construed as a potential conflict of interest.

## Supporting information


Data S1.

## Data Availability

No datasets were generated or used in this study.
