# Supplementary material for: Cautionary tales on the use of proxies to estimate body size and form of extinct animals
Source: Ecol Evol. 2024 Sep 2;14(9):e70218. doi: 10.1002/ece3.70218 (PMC11368419; doi:10.1002/ece3.70218)
Supplement: Supplementary file 1 — Data S1. [file ECE3-14-e70218-s001.docx]

**Supplementary material for ‘Cautionary tales on the use of proxies to estimate body size and form of extinct animals’**

Joel H. Gayford^1,2,*^, Russell K. Engelman^3^, Phillip C. Sternes^2,4^, Wayne M. Itano^5^, Mohamad Bazzi^6^, Alberto Collareta^7,8^, Rodolfo Salas-Gismondi^9,10^, Kenshu Shimada^11,12^

¹ Department of Life Sciences, Silwood Park Campus, Imperial College London, London, United Kingdom

^2^ Shark Measurements, London, United Kingdom,

^3^ Department of Biology, Case Western Reserve University, Cleveland, USA

^4^ Department of Evolution, Ecology and Organismal Biology, University of California, Riverside, California, USA.

^5^ Museum of Natural History, University of Colorado, Boulder, CO 80305, USA

^6^ Department of Earth and Planetary Sciences, Stanford University, Stanford, CA 94305, USA.

^7^Dipartimento di Scienze della Terra, Università di Pisa, Via S. Maria 53, 56126 Pisa, PI, Italy

^8^ Museo di Storia Naturale, Università di Pisa, Via Roma 79, 56011 Calci, PI, Italy

^9^ Facultad de Ciencias y Filosofía/Centro de Investigación para el Desarrollo Integral y Sostenible, Laboratorios de Investigación y Desarrollo, Universitad Peruana Cayetano Heredia Lima, Lima, Perú

^10^ Departamento de Paleontología de Vertebrados, Museo de Historia Natural-Universidad Nacional Mayor de San Marcos, Lima, Perú

^11^ Department of Environmental Science and Studies, DePaul University, 1110 West Belden Avenue, Chicago, Illinois 60614, USA; Department of Biological Sciences, DePaul University, 2325 North Clifton Avenue, Chicago, Illinois 60614, USA;

^12^ Sternberg Museum of Natural History, Fort Hays State University, Hays, Kansas 67601, USA

Corresponding author email: [jhg19@ic.ac.uk](mailto:jhg19@ic.ac.uk)

**Table S1. Extinct taxa of unusually large size which 1) have been the subject of attempts to estimate their body size and/or form from fragmentary material, 2) have received a significant amount of research or popular attention due to their unusual size, or 3) for which previous estimates of size/shape have been controversial. This table is the same as Table 1 in the main document, but it also includes key reference sources and additional notes. * = Taxa for which body size/form estimates have been controversial, see numbered notes for more details.**

| **Higher Taxa** | **Example Taxa** | **Key References** |
| --- | --- | --- |
| Cephalopoda: Nautiloidea | *Endoceras/Cameroceras*^1,4^*, *Rayonnoceras*^2^*^,^** | Flower (1955); Klug et al. (2014); Manger et al. (1999); Pohle and Klug (2017); Teichert and Kummel (1960) |
| Cephalopoda: Belemnitida | *Megateuthis* | Klug et al. (2024) |
| Cephalopoda: Coleoidea | *Enchoteuthis* (“*Tusoteuthis*”)^3,^* | Fuchs et al. (2020); Larson (2010) |
| Arthropoda: Radiodonta | *Anomalocaris*^5,^* | Briggs (1972); Whittington and Briggs (1997) |
| Arthropoda: Eurypterida | *Jaekelopterus*^6,^**, Pterygotus* | Braddy (2023); Braddy et al. (2007); Kaiser and Klok (2008) |
| Arthropoda: Myriapoda | *Arthropleura*^7^ | Davies et al. (2022) |
| Arthropoda: Insecta | *Meganeura*^8,^*, *Meganeuropsis*^8,^* | Cannell (2018) |
| Placodermi: Arthrodira | *Dunkleosteus*^9,^***, *Titanichthys*^10,^**, Glyptaspis*^11,^* | Dean (1909); Engelman (2023a, 2023b, In Press); Ferrón et al. (2017) |
| Chondrichthyes: Eugeneodontiformes | *Helicoprion*^7^, *Edestus*, *Parahelicoprion*^12,^* | Lebedev (2009); Naugolnykh (2018); Tapanila and Pruitt (2013, 2019); Tapanila et al. (2013); Zangerl (1981) |
| Chondrichthyes: Orodontiformes | *Orodus*^13,^* | Zangerl (1981) |
| Chondrichthyes: Ctenacanthiformes | *Saivodus*^14^, *Ctenacanthus*^15,^*, the “Texas Supershark”^16,17^ | Engelman (2023a); Ginter (2010); Hodnett et al. (2022); Maisey et al. (2017) |
| Chondrichthyes: Rajiformes | *Onchopristis*^18,^* | Villalobos-Segura et al. (2021) |
| Chondrichthyes: Lamniformes | megalodon (*Otodus megalodon*)^7,^* | Cooper et al. (2022); Cooper et al. (2020); Sternes et al. (2023) |
| Osteichthyes: Pachycormiformes | *Leedsichthys*^19,^* | Liston et al. (2013) |
| Osteichthyes: Salmoniformes | *Oncorhynchus rastrosus*^20^ | Stearley and Smith (2016) |
| Sarcopterygii | *Rhizodus*^21,^*, *Hyneria*^22^*, Mawsonia*^23,^* | Jeffery (1998); Toriño et al. (2024); Young et al. (2013) |
| Extinct Temnospondyli | *Prionosuchus*^24,^*, *Eryops*, Mastodonsauridae, the “Precious of Lesotho”^15,25^ | Cox and Hutchinson (1991); Damiani and Steyer (2005); Hart et al. (2022) |
| Anura | *Beelzebufo*^26,^*** | Evans et al. (2014); Evans et al. (2008) |
| Squamata: terrestrial “lizards” | megalania (*Varanus priscus*)^27,^*, *Barbaturex*^28^ | Head et al. (2013); Molnar (2004) |
| Squamata: Serpentes | *Titanoboa, Vasuki* | Datta and Bajpai (2024); Head et al. (2009) |
| Squamata: Mosasauridae | *Mosasaurus*^29,^*, *Tylosaurus* | Cleary et al. (2018); Everhart et al. (2016); Fanti et al. (2014); Grigoriev (2014); Russell (1967) |
| Testudines | *Stupendemys*^30^*, Caninemys*, *Peltocephalus maturin*, Meolaniidae, giant tortoises (*Megalochelys atlas*^31,^*) | Bourke (2016); Brown (1931); Cadena et al. (2020); Ferreira et al. (2024); Vlachos and Rabi (2018) |
| Ichthyopterygia | *Cymbospondylus*, *Shonisaurus*^32,^***, “*Shastasaurus*” *sikanniensis*^33,^***, *Ichthyotitan*^16,34^*, the “Aust Colossus”^16^ | Camp (1980); Kosch (1990); Lomax et al. (2018); Lomax et al. (2024); Nicholls and Manabe (2004); Sander et al. (2021) |
| Sauropterygia | *Liopleurodon*^35,^**, Kronosaurus*^35,^*, *Pliosaurus*^35,^* | Benson et al. (2013); Knutsen et al. (2012); Martill et al. (2023); McHenry (2009) |
| Crocodyliformes: Thallatosuchia | *Machimosaurus*^36,^* | Fanti et al. (2016); Young et al. (2016) |
| Crocodyliformes: Notosuchia | *Barinasuchus*^37^*, Kaprosuchus*^38,^* | Molnar and Vasconcellos (2016); Sereno and Larsson (2009) |
| Crocodyliformes: stem Neosuchia | *Aegisuchus*^39,^*, *Sarcosuchus*^40,^* | Holliday and Gardner (2012); O’Brien et al. (2019); Sereno et al. (2001) |
| Crocodyliformes: Crocodylia | *Deinosuchus*^41,^*, *Purussaurus*^42,^*, *Mourasuchus*^42,^* | Aureliano et al. (2015); Cidade et al. (2019); Farlow et al. (2005); Moreno-Bernal (2007); Paiva et al. (2022); Schwimmer (2002) |
| Pterosauria | *Pteranodon*, *Quetzalcoatlus*^43,^*, *Arambourgiana*^43,^*, *Hatzegopteryx*^43,^* | Henderson (2010); Witton (2008, 2010); Witton and Habib (2010) |
| Dinosauria: flightless Avialae | Gastormithidae^44,^*, Dinornithidae^45,^*, Aepyornithidae, Dromornithidae^46,^*, Phorusrhacidae | Alvarenga and Höfling (2003); Angst and Buffetaut (2017); Buffetaut and Angst (2013); Chiappe and Bertelli (2006); Murray and Vickers-Rich (2004); Nguyen et al. (2010); Worthy and Holdaway (2002) |
| Dinosauria: volant Avialae | *Pelagornis*^47,^*, *Argentavis*^48,^* | Campbell and Tonni (1983); Chatterjee et al. (2007); Ksepka (2014); Mayr and Rubilar-Rogers (2010); Witton (2018) |
| Dinosauria: Sphenisciformes | *Anthropornis*^49,^*, *Pachydyptes*^49,^*, *Kairuku* | Ksepka et al. (2012) |
| Dinosauria: non-avian Theropoda | Many, e.g., *Tyrannosaurus*^50,^*, *Spinosaurus*^51,^*, several Abelisauridae (*Abelisaurus*^52,^*, *Ekrixinatosaurus*^52,^***) | Dal Sasso et al. (2005); Grillo and Delcourt (2017); Hartman (2013); Ibrahim et al. (2014); Mallon and Hone (2024); Newman (1970); Therrien and Henderson (2007) |
| Dinosauria: Sauropoda | Many, e.g., *Dreadnoughtus*^53,^*, “*Seismosaurus*”^54,^*, *Futalognkosaurus*^55,^*, *Bruhathkayosaurus*^4,15,56 ,^*, *Maraapunisaurus*^4,15,57 ,^* | Bates et al. (2015); Calvo et al. (2008); Calvo et al. (2007); Carpenter (2018); Herne and Lucas (2006); Lacovara et al. (2014) |
| Dinosauria: Ornithischia | Many, e.g., *Triceratops*, *Stegosaurus*^58^ | Brassey et al. (2015) |
| Synapsida: Dicynodontia | *Lisowicia*^59,^* | Romano and Manucci (2021); Sulej and Niedźwiedzki (2019) |
| Mammalia: Dasyuromorphia | *Thylacinus cynocephalus*^60,^*, *T. potens*^60,^* | Rovinsky et al. (2020) |
| Mammalia: Diprotodontia | *Thylacoleo*^61,^*, *Diprotodon*^62^, *Procoptodon*^63,^* | Helgen et al. (2006); Musser (2018); Wroe et al. (2004); Wroe et al. (2003); Wroe et al. (1999) |
| Mammalia: Proboscidea | *Palaeoloxodon*^4,64,^**, Mammuthus* | Larramendi (2015) |
| Mammalia: Rhinoceratoidea | *Paraceratherium*^65,^*, *Elasmotherium*^66^ | Fortelius and Kappelman (1993); Prothero (2013); Titov et al. (2021) |
| Mammalia: Cetacea | *Perucetus*^7,^***, *Livyatan*^67,^* | Bianucci et al. (2023); Lambert et al. (2010); Motani and Pyenson (2024) |
| Mammalia: Hyaenodonta | *Megistotherium*^68,^*, *Hyainailouros*^68,^**, Simbakubwa*^68^ | Borths and Stevens (2019); Savage (1973) |
| Mammalia: Carnivora | *Arctotherium*, *Arctodus*, *Smilodon, Megalictis*^69,^* | Christiansen (1999); Christiansen and Harris (2005); Figueirido et al. (2010); Hunt and Skolnick (1996); Manzuetti et al. (2020); Soibelzon and Schubert (2011); Valenciano Vaquero (2017) |
| Mammalia: Rodentia | *Josephoartigasia*^70,^***, *Phoberomys*^70,^*, *Telicomys*^70,^***, *Casteroides* | Engelman (2022); Hopkins (2008); Millien (2008); Millien and Bovy (2010); Reynolds (2002); Rinderknecht and Blanco (2008); Sánchez-Villagra et al. (2003) |
| Mammalia: Primates | *Gigantopithecus*^15,71,*^ | Zhang and Harrison (2017) |

**Table S2. Examples of changes in estimated size in taxa discussed here. For the sake of fairness, only cases where firm estimates for the original and revised size estimates were presented in the primary literature were considered. “Difference in Estimates” represent the difference between the original estimate and the revised value and are calculated as (revised estimate – original estimate)/original estimate to better show the direction of change (i.e. a value of -50% means the revised value is half the size of the original). When values are reported as ranges the midpoint was taken for easier comparison. Disparities in abelisauroid lengths can be found in Table S6.**

| Taxon | Variable | Original Estimate | Revised Estimate | Difference in Estimates | References |
| --- | --- | --- | --- | --- | --- |
| *Endoceras/Cameroceras* | total length | 9.14 m | ~ 5 m | -45.3 % | Flower (1955), Klug et al. (2014) |
| *Enchoteuthis* spp. | total length | 6–15 m | 3 m | -71.4% | Fuchs et al. (2020); Larson (2010) |
| *Anomalocaris canadensis* | total length | 1 m | 40 cm | -60.0% | Briggs (1972); Whittington and Briggs (1997) |
| *Acutiramus bohemicus* | total length | 2.5 m | 2.1 m | -19.0% | Braddy et al. (2007) |
| *Jaekelopterus rhenianae* | total length | 2.46 m | 2.59 m | +5.0% | Braddy et al. (2007), Braddy (2023) |
| *Dunkleosteus terrelli* | total length | 6–10 m | 3.4–4.1 m | -53.1% | Engelman (2023a, 2023b) |
| *Glyptaspis verrucosa* | total length | 8 m | 2.2–2.3 m | -71.9% | Sallan and Galimberti (2015), Pimiento et al. (2024), present study |
| *Ctenacanthus tumidus* | total length | 5–6 m | 4.20 m | -23.6% | Ginter (2010), Long (2024), Engelman (2023a) |
| *Leedsichthys problematicus* | total length | 27.6 m | ~16.5 m | -40.2% | Martill (1986), Martill (1988), Liston et al. (2013) |
| *Rhizodus hibberti* | total length | 6–7 m | 5.0–5.6 m | -18.5% | Andrews (1985), Jeffery (1998), Engelman (2023a) |
| *Mawsonia* spp. | total length | 5–6 m | 5.1 m? | -7.2% | Toriño et al. (2024) |
| *Beelzebufo ampinga* | snout-vent length | 42.5 cm | 23.2 cm | -45.4% | Evans et al. (2008), Evans et al. (2014) |
| *Mosasaurus hoffmanni* | total length | 15–17 m | 11–12 m | -28.1% | Fanti et al. (2014), Grigoriev (2014), Everhart et al. (2016) |
| *Machimosaurus rex* | total length | 10 m | 7.18 m | -28.2% | Fanti et al. (2016), Young et al. (2016) |
| *Machimosaurus hughii* | total length | 9 m | 6.9 m | -23.3% | Young et al. (2014), Young et al. (2016) |
| *Kaprosuchus saharicus* | total length | 6 m | 2.42–3.77 m | -48.4% | Sereno and Larsson (2009), present study |
| *Sarcosuchus imperator* | total length | 11–12 m | 7.64–8.97 m | -27.8% | Sereno et al. (2001), O’Brien et al. (2019) |
| *Deinosuchus riograndensis* | total length | 15.24 m | 10.6 m | -43.7% | Colbert and Bird (1954), Farlow et al. (2005) |
| *Mourasuchus amazonensis* | total length | 9.04–9.90 m | 5.27 m | -44.4% | Cidade et al. (2019), Paiva et al. (2022) |
| *Mourasuchus arendsi* | total length | 7.4–8.1 m | 4.25 m | -45.2% | Cidade et al. (2019), Paiva et al. (2022) |
| *Mourasuchus atopus* | total length | 6.07–6.52 m | 2.80 m | -55.6% | Cidade et al. (2019), Paiva et al. (2022) |
| *Mourasuchus pattersoni* | total length | 8.53–9.33 m | 4.83 m | -45.9% | Cidade et al. (2019), Paiva et al. (2022) |
| *Purussaurus brasiliensis* | total length | 12.5 m | 8.00 m | -36.0% | Aureliano et al. (2015), Paiva et al. (2022) |
| *Purussaurus mirandai* | total length | 7.11–8.01 m | 6.70 m | -11.3% | Scheyer et al. (2019), Paiva et al. (2022) |
| *Purussaurus neivensis* | total length | 8.01 m | 4.65 m | -41.9% | Moreno-Bernal (2007), Paiva et al. (2022) |
| *Quetzalcoatlus northropi* (and other giant pterosaurs) | wingspan | 11.5–15.5 m | 10.5 m | -22.2% | Lawson (1975), Frey and Martill (1996), Witton and Habib (2010) |
| *Dromornis stirtoni* | body mass | 627 kg (avg.),  829 kg (max) | 519.5 kg (avg.),  713.9 kg (max) | -17.1% (avg.),  -13.8% (max) | Murray and Vickers-Rich (2004); Nguyen et al. (2010) |
| *Argentavis magnificens* | wingspan | 7.0–7.6 m | 5.09–6.09 m | -30.6% | Campbell and Tonni (1980), Ksepka (2014) |
| *Dreadnoughtus schrani* | body mass | 59300 kg | 27741 kg | -53.2% | Lacovara et al. (2014), Bates et al. (2015) |
| *Maarapunisaurus fragillimus* | total length | ~ 60 m | 30.3 m | -49.5% | Carpenter (2018) |
| *“Seismosaurus”* (*Diplodocus*) *hallorum* | total length | 39–52 m | 30.5–35 m | -28.0% | Gillette (1994); Herne and Lucas (2006) |
| *Lisowicia bojani* | body mass | 9330 kg | 5880 | -37.0% | Sulej and Niedźwiedzki (2019), Romano and Manucci (2021) |
| *Thylacinus potens* | body mass | 41–52.3 kg | 22.2–27.9 kg | -46.3% | Rovinsky et al. (2020) |
| *Thylacoleo carnifex* | body mass | ~45 kg | 101–130 kg | +56.7% | Wroe et al. (1999) |
| *Perucetus colossus* | body mass | 180 tons | 60–114 tons | -51.7% | Bianucci et al. (2023), Motani and Pyenson (2024) |
| *Megalictis ferox* | body mass | ~70–120 kg | 30–54 kg | -55.8% | Hunt and Skolnick (1996); Matthew (1907); Valenciano Vaquero (2017) |
| *Phoberomys pattersoni* | body mass | 731 kg | 150–200 kg | -76.1% | Sánchez-Villagra et al. (2003), Hopkins (2008), Millien and Bovy (2010), Engelman (2022) |
| *Josephoartigasia monesi* | body mass | 1211 kg | ~500 kg | -58.7% | Rinderknecht and Blanco (2008), Millien (2008), Engelman (2022) |

**Table S3. List of possible sources of discrepancies between size estimates mentioned in various size estimate controversies.**

| **Taxon** | **Issues Present/Concerns Raised** |
| --- | --- |
| *Cameroceras/Endoceras* | anecdotal estimates, unreplicable estimates, possible largest material now lost |
| *Enchoteuthis* spp. | choice of proxy taxon, reconstruction issues |
| *Anomalocaris canadensis* | choice of proxy taxon, interpretation of elements |
| *Jaekelopterus rhenianae* | allometry versus isometry, taxon choice |
| *Meganeura moneyi*, *Meganeuropsis* spp. | allometry versus isometry, unit confusion (wingspan versus mass), extrapolation error |
| *Dunkleosteus terrelli* | anecdotal estimates, choice of proxy taxon, unreplicable results, disproportionately large mandibular arch (not crania) |
| *Glyptaspis verrucosa* | anecdotal estimates, unreplicable estimates |
| *Helicoprion* spp. | limited size-informative remains, choice of proxy taxon, choice of anatomical proxy, unclear scaling patterns |
| *Parahelicoprion mariosuarezi* | limited size-informative remains, choice of proxy taxon, reconstruction issues, pop cultural distortion, unclear scaling patterns |
| *Ctenacanthus tumidus* | anecdotal estimates, unreplicable estimates, choice of proxy taxon |
| “Texas Supershark” | limited size-informative remains, simple scaling issues |
| *Orodus* spp. | limited size-informative remains, unclear scaling patterns |
| *Onchopristis* spp. | pop cultural distortion |
| *Otodus megalodon* | limited size-informative remains, choice of proxy taxon, unreplicable estimates |
| *Leedsichthys problematicus* | choice of proxy taxon, choice of size proxy, pop cultural distortion |
| *Mawsonia* spp. | choice of proxy taxon, allometry versus isometry |
| *Hyneria lindae* | pop cultural distortion |
| *Prionosuchus plummeri* | pop cultural distortion?, choice of proxy taxon? |
| *Beelzebufo ampinga* | unclear; allometry versus isometry(?), disproportionately large crania(?) |
| *Varanus priscus* | choice of proxy taxon |
| *Mosasaurus hoffmanni* | unclear scaling patterns, simple scaling issues |
| *Megalochelys atlas* | reconstruction issues |
| *“Shastasaurus” sikanniensis* | choice of proxy taxon, lapsus? (quarry map starting at 4 m) |
| *Ichthyotitan severnensis* | limited-size informative material, unclear scaling patterns |
| Large Pliosauridae (*Liopleurodon*, *Kronosaurus*, *Pliosaurus*) | anecdotal estimates, unclear scaling relationships, reconstruction issues (*Kronosaurus* and “Cumnor mandible”), identification of material (Petersborough vertebra) |
| *Machimosaurus* spp. | allometry versus isometry, disproportionately large crania |
| *Kaprosuchus saharicus* | anecdotal estimates, unreplicable estimates |
| *Sarcosuchus imperator* | unreplicable estimates |
| *Deinosuchus* spp. (mostly *D. riograndensis*) | anecdotal estimates, reconstruction issues (see text) |
| *Mourasuchus amazonensis*, *M. arendsi*, *M. atopus*, and *M. pattersoni* | inclusion of juveniles in regression models, non-linear allometry(?) |
| *Purussaurus brasiliensis*, *P. mirandai*, *P. neivensis* | inclusion of juveniles in regression models, non-linear allometry(?) |
| *Quetzalcoatlus northropi*, *Arambourgiana philadelphiae*, *Hatzegopteryx thambena* | allometry versus isometry, choice of proxy taxon, extrapolation error |
| Dinornithidae | reconstruction issues |
| *Dromornis stirtoni* (and some other Dromornithidae) | lapsus (error in converting regression equation) |
| *Pelagornis* spp. | anecdotal estimates |
| *Argentavis magnificens* | limited size-informative material (but see text), reconstruction issues, choice of size proxy (using estimated body mass to calculate wingspan) |
| *Anthropornis* spp., *Pachydyptes* spp. | choice of proxy taxon, choice of size proxy |
| *Tyrannosaurus rex* | reconstruction issues (see text) |
| *Spinosaurus aegyptacus* | allometry versus isometry, reconstruction issues(?) |
| Large abelisaurids (*Abelisaurus*, *Ekrixinatosaurus*) | allometry versus isometry, simple scaling issues, choice of proxy taxon, choice of size proxy |
| *Dreadnoughtus schrani* | non-linear allometry(?) |
| *Maarapunisaurus fragillimus* | choice of proxy taxon, possible largest material now lost |
| *Bruhathkayosaurus matleyi* | possible largest material now lost |
| *Lisowicia bojani* | choice of anatomical proxy? |
| *Thylacinus cynocephalus* | anecdotal estimates |
| *Thylacoleo atrox* | anecdotal estimates |
| *Procoptodon goliah* | unit confusion (standing height versus maximum reach) |
| *Diprotodon optatum* | anecdotal estimates |
| *Palaeoloxodon namadicus* | possible largest material now lost |
| *Paraceratherium* spp. | reconstruction issues, simple scaling issues |
| *Perucetus colossus* | unreplicable estimates (volumetric versus regression), allometry versus isometry, choice of proxy taxon (sirenian versus cetacean), extrapolation error |
| *Livyatan melvillei* | choice of proxy taxon |
| *Megistotherium ostothlastes* | anecdotal estimates, choice of size proxy, unreplicable estimates, limited size-informative remains (cranio-dental remains expected to produce overestimates) |
| *Megalictis ferox* | unreplicable estimates, pop cultural distortion, unit confusion (a *skull* the size of a bear/jaguar, not necessarily a body) |
| *Phoberomys pattersoni, Josephoartigasia monesi, Telicomys* spp. | choice of size proxy, statistical methodology, non-linear allometry |
| *Gigantopithecus blacki* | limited size-informative material, choice of proxy taxon, choice of size proxy |

Anecdotal estimates — Estimated sizes are reported without providing necessary data or quantifications to replicate these values

Unreplicable estimates — Attempts to reproduce these (often anecdotal) estimates through other means fail

Choice of proxy taxon — The choice of proxy taxon used to estimate body size/form has been called into question or shown to be unreliable

Choice of size proxy — The reliability of the morphological proxy used to estimate body size/form in this taxon has been called into question or have a dubious relationship with body size

Allometry versus isometry — Studies estimate body size/form through simple scaling when relationship shows signs of being positively/negatively allometric

Limited size-informative material — Taxon is only known from limited, fragmentary material preserving a limited number of size-informative characters

Pop cultural distortion — Confusion over size/form does not come from primary literature, but from presentation in grey literature and popular culture

Simple scaling issues — Issues where the original size estimate was calculated by scaling from simple ratios, but the underlying assumptions of those ratios have been challenged

**Table S4. Estimates of total length for *Kaprosuchus saharicus* scaling from skull length (DSL) and head width (HW).**

| Reference | Variable | Value | Equation | Est. TL (cm) | Primary Dataset |
| --- | --- | --- | --- | --- | --- |
| O’Brien et al. (2019) | HW | 21.38 | $\ln\left( \text{TL} \right)=0.8024*\ln\left( \text{HW} \right)+3.05$ | 246.5 | Various Crocodylia |
| Paiva et al. (2022) | DSL | 50.7 | $log10 \left( \text{TL, in mm} \right)=0.875*log10 \left( \text{HW, in mm} \right)+1.209$ | 376.6 | Various Crocodylia |
| Paiva et al. (2022) | HW | 21.38 | $log10 \left( \text{TL, in mm} \right)=0.929*log10 \left( \text{HW, in mm} \right)+1.220$ | 242.4 | Various Crocodylia |
| Woodward et al. (1995) | DSL | 50.7 | $\ln\left( \text{TL, in cm} \right)=0.95811*\ln\left( \text{HW, in cm} \right)+2.132494$ | 362.8 | *Alligator mississippiensis*, male |
| Woodward et al. (1995) | DSL | 50.7 | $\ln\left( \text{TL, in cm} \right)=0.98016*\ln\left( \text{HW, in cm} \right)+2.070667$ | 371.9 | *Alligator mississippiensis*, female |
| Greer (1974) | DSL | 50.7 | $log10 \left( \text{TL, in in} \right)=0.929*log10 \left( \text{HW, in in} \right)+1.220$ | 368.6 | *Crocodylus porosus* |

**Table S5. Body size estimates of giant Miocene South American caimanines under a variety of methods (see text for details). All studies under “Study” used OLS except for Scheyer et al. (2019)**

| Taxon | Specimen | Length | Study | PGLS Lengths in Paiva et al. (2022) | OLS Lengths in Paiva et al. (2022) |
| --- | --- | --- | --- | --- | --- |
| *Mourasuchus amazonensis* | DGM 526-R | 9.04–9.90 | Cidade et al. (2019) | 5.27 (4.65–5.99) m | 6.25–7.68 m |
| *Mourasuchus arendsi* | CIIAP-1297 | 7.4–8.1 | Cidade et al. (2019) | 4.25 (3.76–4.79) m | 4.88–7.38 m |
| *Mourasuchus atopus* | UCMP 38012 | 6.07–6.52 | Cidade et al. (2019) | 2.80 (2.51–3.13) m | 3.02–5.10 m |
| *Mourasuchus pattersoni* | MCNC-PAL-110-72V | 8.53–9.33 | Cidade et al. (2019) | 4.83 (4.29–5.49) m | 5.64–7.35 m |
| *Purussaurus brasiliensis* | UFAC 1403 | 12.5 m,  9.49 m | Aureliano et al. (2015),  Moreno-Bernal (2007) | 8.00 (7.62–9.28) m | 9.26–10.01 m |
| *Purussaurus mirandai* | CIAAP-1369 | 7.11–8.01 | Scheyer et al. (2019) | 6.70 (6.39–7.65) m | 5.37–6.33 m |
| *Purussaurus neivensis* | UCMP 39704 | 8.01 m | Moreno-Bernal (2007) | 4.65 (4.46–5.23) m | 6.25–7.68 m |

**Table S6. Estimated total length for abelisauroid theropods from Grillo and Delcourt (2017) using allometric regression equations compared to those reported in the previously published literature. This table is essentially a summary of tables 1 and 7 and figure 7 in Grillo and Delcourt (2017)**

| **Taxon** | **Literature lengths** | **Length Sources (according to Grillo and Decourt 2017)** | **Estimated lengths** | **Difference in Estimates** |
| --- | --- | --- | --- | --- |
| **Total length much smaller than previously published literature (20% difference or more)** | | | | |
| *Abelisaurus comahuensis* | 10 m | Bonaparte and Novas (1985); Paul (2010) | 7.36 ± 0.74 m | -26.4% |
| *Austrocheirus isasii* | 7.0–8.0 m | Ezcurra et al. (2010) | 5.54 ± 0.34 m | -26.1% |
| *Ekrixinatosaurus novasi* | 7.0–8.0 m  10.0–11.0 m | Calvo et al. (2004)  Juárez Valieri et al. (2010) | 7.35 ± 0.81 m^[[1]](#footnote-1)^ | -2.0%,  -30.0% |
| *Majungasaurus crenatissimus* | 6.0–7.0 m^[[2]](#footnote-2)^ | Krause et al. (2007) | 5.58 ± 0.25 m (FMNH PR 2100),  5.39 ± 0.52 m (FMNH PR 2278),  4.74 ± 0.29 m (UA 8678) | -14.2%,  -17.1%  -27.1% |
|  | 6.4–8.8 m?^2^ (FMNH PR 2100) | Sampson and Witmer (2007) | 5.58 ± 0.25 m (FMNH PR 2100) | -26.6% |
| *Rajasaurus primus* | 7.6–9.0 m | Wilson et al. (2003) | 6.57 ± 0.37 m | -20.8% |
| *Rahiolisaurus guajaratensis* | 8.0 m (ISIR 550/554/557) | Novas et al. (2010) | 6.27 m | -21.6% |
| *Rugops primus* | 6.0 m | Paul (2010); Sereno et al. (2004) | 4.39 ± 0.39 m | -26.8% |
| **Total length roughly equal to estimates in previously published literature (within ± 10%)** | | | | |
| *Arcovenator scotae* | 5.0–6.0 m | Tortosa et al. (2014) | 4.83 ± 0.47 m | +12.2% |
| *Aucasaurus garridoi* | 5.5–6.0 m | Coria et al. (2002) | 6.10 ± 0.25 m | +6.1% |
| *Carnotaurus sastrei* | 8.0–9.0 m | Bonaparte et al. (1990); Méndez (2012) | 7.75 ± 0.35 m | -8.8% |
| *Dahalokely tokana* | 3.5 ± 0.9 m^[[3]](#footnote-3)^ | Farke and Sertich (2013) | 3.84 ± 0.23 m | +9.7% |
| *Eoabelisaurus mefi* | 6.0–6.5 m | Pol and Rauhut (2012) | 5.82 ± 0.56 m | -6.9% |
| *Ligabueino andesi* | 0.74 m | Bonaparte (1996) | 0.79 m | +6.8% |
| *Masiakasaurus knopfleri* | 2.1 m (FMNH PR 2841) | Carrano et al. (2001) | 1.98 ± 0.04 m (FMNH PR 2841) | -5.7% |
|  | 1.5 m (FMNH PR 2485) |  | 1.74 ± 0.12 m (FMNH PR 2485) | +16.0% |
|  | 1.8 m (UA 8680) |  | 2.10 ± 0.05 m (UA 8680) | +16.7% |
| *Skorpiovenator bustingorryi* | 6.0–7.0 m | Canale et al. (2009) | 6.22 ± 0.38 m | -4.3% |
| *Xenotarsosaurus bonapartei* | 5 m | Lamanna et al. (2002) | 5.43 ± 0.28 m | +8.6% |
| **Estimated total length much greater than previously published literature (10%+)** | | | | |
| *Genusaurus sisteronis* | 3.2 m | Accarie et al. (1995) | 3.56 m | +11.3% |
| *Pycnonemosaurus nevesi* | 7.0–8.0 m | Kellner and Campos (2002) | 8.93 ± 0.31 m | +19.1% |
| *Velocisaurus unicus* | 1.2 m | Bonaparte (1991) | 1.51± 0.05 m | +25.8% |

# Institutional Abbreviations

**AMNH** – American Museum of Natural History, New York, USA

**CMNH** – Cleveland Museum of Natural History, Cleveland, USA

**CIAAP** – Centro de Investigaciones Antropológicas, Arqueológicas y Paleontológicas, Universidad Nacional Experimental Francisco de Miranda, Coro, Venezuela

**DGM** – Museu de Ciencias da Terra, Departamento Nacional de Produção Mineral, Rio de Janeiro, Brazil

**FMNH** – Field Museum of Natural History, Chicago, USA

**FSAC-KK** – Palaeontological Collections of the Department of Geology of the Faculty of Sciences Aïn Chock, Hassan II University of Casablanca, Morocco

**ISIR** – Indian Statistical Institute, Kolkata, India

**MAAT** – Museo Alberto Arvelo Torrealba, Barinas, Venezuela

**MCZ** – Museum of Comparative Zoology, Cambridge, USA

**MCNC-PAL** – Museo de Ciéncias Naturales de Caracas, Caracas, Venezuela

**MLP** – Museo de La Plata, La Plata, Argentina

**MNHN** – Muséum National d’Histoire Naturelle, Paris, France

**MSNM** – Museo Civico di Storia Naturale di Milano, Milan, Italy

**NHMUK** –British Museum of Natural History, London, UK

**NMS** – National Museum of Scotland, Edinborough, UK

**OUM** – Oxford University Museum, Oxford, UK

**PETMG** – Peterborough Museum, Peterborough, Cambridgeshire, England

**QM** – Queensland Museum, South Brisbane, Australia

**PMO** – University of Oslo Natural History Museum, Oslo Norway

**SFM** – Shinshushinmachi Fossil Museum, Shinshushinmachi, Nagano Prefecture, Japan

**TMM** – Texas Memorial Museum, Austin, USA

**UA** – University d’Antananarivo, Antananarivo, Madagascar

**UANL-FCT** – Universidad Autonóma de Nuevo León, Linares, Mexico

**UCMP** – University of California Museum of Paleontology, Berkeley, USA

**UFAC** – Universidade Federal do Acre, Rio Branco, Brazil

# Additional Observations on Data Presented

The data presented here are not an exhaustive catalog of taxa where size/form estimates have been controversial. Instead, they represent a relatively surface-level survey of taxa for which this is the case based on readily available literature, a Google Scholar search, our own observations with taxa we are familiar with (read = biased towards non-teleost fishes and mammals, with poor coverage of dinosaurs in particular), and from conversations with colleagues across a wide array of subdisciplines in paleontology. A more thorough search would likely turn up many more cases as well as expand on the examples presented here. As a result, we cannot say we are familiar with every dispute over body size/form estimation; it is quite likely many taxa listed here as “non-controversial” (especially the non-mammalian tetrapods) have had historical controversies over their size/form we are unaware of or may have their current estimates called into question by future research. Indeed, we know of several additional cases of prehistoric organisms whose size has likely been overestimated that we did not list here due to space and/or the necessary data being unpublished and therefore not possible to fairly evaluate. The cases presented here are merely meant to show the extent and phylogenetic breadth of the problem at hand.

Sensationalized claims of exaggerated body size in the scientific literature are not unique to extinct animals, as they have also occurred in a number of living species (see discussions surrounding *Varanus komodoensis*, Auffenberg, 1981; *Python bivittatus*, Barker et al., 2012; *Crocodylus porosus*, Greer, 1974; *Eunectes murinus* and other large snakes, Murphy & Henderson, 1997; *Architeuthis dux* and *Mesonychoteuthis hamiltoni*, O'Shea & Bolstad, 2019; *Carcharodon carcharias*, Randall, 1973; *Thylacinus cynocephalus*, Rovinsky et al., 2020; *Dermochelys coriacea*, Wood, 1976; *Alligator mississippiensis*, Woodward et al., 1995). Like extinct animals, this phenomenon most frequently occurs in megafauna. Among extant organisms, size controversies tend to occur in pelagic and/or deep-sea organisms that only occasionally interact with humans (whales, large sharks, large cephalopods) or taxa with indeterminate growth and/or type III survivorship curves where the largest possible individuals are rarely seen (crocodilians, Komodo dragons). Thus, these extant cases are similar to fossil taxa in that our knowledge of their natural history (and normal size range) is fragmentary, opening the door to potential overestimation.

Although most claims of spectacular size in extinct organisms involve organisms significantly larger than their nearest extant analogues, organisms significantly smaller than their nearest living analogues also exist and may receive a significant amount of attention for similar reasons (Bloch et al., 1998; Brown et al., 2004; Xing et al., 2020). However, from our observations the body size and form of these taxa seems to be less controversial. Claims of spectacularly diminutive sizes tend to be more commonly seen in living organisms than fossil ones (Bolaños et al., 2024; Rüber et al., 2007), likely because organisms of extremely small size may not fossilize well.

Based on our survey of size estimate controversies in the primary literature and their downstream gray literature and pop cultural depictions, several patterns are clear:

1. Dubious or controversial size estimates are widespread in paleontology. Examples can be identified in almost every major group of vertebrates (placoderms, chondrichthyans, bony fishes, amphibians, squamates, marine reptiles, crocodylomorphs, dinosaurs [incl. birds], and synapsids), and several groups of invertebrates (cephalopods and arthropods).
2. Because of the highly specialized nature of paleontology and the sheer timespan involved, few research groups are associated with more than one dubious size estimate (though a few names appear more than once). Thus, this represents a broader, systematic problem in paleontology rather than the actions of a handful of researchers.
3. Controversies in body size/form seem to be most common in the very largest members of a given group. Similar revisions and controversies around the body size/form of smaller members of the same group are less common (or, perhaps more cynically, they may occur but are not be considered sufficiently noteworthy to warrant special attention compared to biologically extreme taxa). Some of the same issues with reproducibility and methodology seem to occur in less sensationalized taxa (Bochaton & Kemp, 2017), but it is unclear if it is as widespread as it is in charismatic megafauna.

There are other reasons this might be expected. Large taxa are expected to be rare and only represented by fragmentary material in most collections due to difficulty in collecting and curating large specimens (Flower, 1955); this is a problem for both extant and extinct megafauna (De Maddalena et al., 2003). Thus, large taxa are expected to be known from less complete material than their smaller relatives, requiring the need for estimation. Estimate uncertainty also typically increases the further one extrapolates from the scaling dataset (Millien, 2008; Schmidt-Nielsen, 1984), and thus the very largest taxa in a given clade would require the most extrapolation compared to taxa known from complete remains.

Another factor may be that many of these controversial estimates tend to assume their anatomical proxy of choice scales isometrically. Indeed, many overestimates seem to involve either scaling from anteroposterior skull or mandible length (Toriño et al., 2024; A. Cau pers. comm. February 2023; Young et al., 2016) or long bone circumferences (Bates et al., 2015; Brassey et al., 2015; Romano & Manucci, 2021). Some studies have suggested these features show positively allometry or non-linear allometric scaling (Engelman, 2022; Nelson et al., 2023; Paiva et al., 2022), which suggests these variables may have a tendency to slightly overestimate body size in very large taxa. Thus, model choice may play a significant role.

1. Body size estimates almost always go down over time. The general pattern is that initial studies set a high-water mark and later studies revise these estimates to more modest values (Table S2). Similarly, once revised, body size estimates rarely return to their former extremes. Body size estimates usually do not increase unless new material pertaining to larger individuals than previously known is discovered (Cadena et al., 2020; Mayr & Rubilar-Rogers, 2010; Persons et al., 2020). Some of the few examples we have seen where later analyses result in well-supported, higher estimates of body size are the Australian marsupials *Thylacoleo* and *Diprotodon* (Wroe et al., 2004; Wroe et al., 1999), *Jaekelopterus* (barely, at a ~5% increase in length; Braddy, 2023), *Pycnonemosaurus* (increase in length of 19%; Grillo & Delcourt, 2017), and megalodon, depending on how one interprets the conclusions of Sternes et al. (2024).

It is this near-uniform directionality (rather than initial size estimates showing random error towards either overestimation or underestimation) that suggests this pattern cannot simply be attributed to the uncertainties and inherent difficulties of working with very fragmentary extinct taxa, but is reflective of deeper patterns. Other authors have made similar observations (Grillo & Delcourt, 2017).

1. Massive fluctuations in estimated size for a given taxon are not uncommon. While most revisions to size estimates are relatively modest, in several cases (Table S3) studies revisiting size estimates using more in-depth methodologies can revise these values to nearly half of their original value. Note as well that because body mass is roughly proportional to length cubed, an increase or decrease in linear dimensions by about 25% translates to a doubling or halving of estimated body mass, respectively (cubic root of 2 is ~1.26).
2. Spurious estimate of body size/form actually matter. In several cases, studies discussing erroneous size/form estimates show how these incorrect values have significant downstream conclusions on our understanding of paleobiology, biomechanics, and evolutionary history (Bianucci et al., 2023; Chatterjee et al., 2007; Engelman, 2023a; Fortelius & Kappelman, 1993; Grillo & Delcourt, 2017; Romano & Manucci, 2021; Rovinsky et al., 2020; Witton & Habib, 2010). To go into more detail with these examples…

- Chatterjee et al. (2007) estimated the flight capability of the teratorn *Argentavis magnificens* using a likely overestimated body mass of 70 kg. Perhaps unsurprisingly, they concluded *Argentavis* “was probably too large…to be capable of continuous flapping flight or standing takeoff under its own muscle power”.
- Engelman (2023a) found that prior suggestions of an explosive increase in vertebrate size across the middle Paleozoic was driven by a number of spurious estimates for various megafaunal fishes (especially Arthrodira), with revised estimates suggesting a much more gradual path to the acquisition of large body size in vertebrates.
- Fortelius and Kappelman (1993) noted that the size of *Paraceratherium* mattered because as the largest known land mammal it helps define the possible upper size limits for mammals.
- Grillo and Delcourt (2017) found that once potentially spurious size estimated had been cleaned up, Abelisauroids showed a clear pattern of body size increase over evolutionary time in the late Cretaceous.
- Romano and Manucci (2021) and Sulej and Niedźwiedzki (2019)noted that the estimated size of *Lisowicia* was important because it set the limits for synapsid size prior to the mammalian radiation of the Cenozoic. Sulej and Niedźwiedzki (2019) had suggested from initial size estimates of *Lisowicia* this meant non-mammalian synapsids reached sizes comparable to large Cenozoic megafauna (elephants). However, Romano and Manucci (2021) noted with their revised size estimates of ~5880 kg, synapsids did not reach sizes similar to elephants (~9000 kg) until the Cenozoic.
- Rovinsky et al. (2020) noted that the old mass estimates for the thylacine (*Thylacinus cynocephalus*) called into question scaling relationships between prey size and predator size in carnivorous mammals (Carbone et al., 1999). Additionally, because this taxon is almost twice the size of the next largest recent dasyuromorphian, it has an outsized influence on regression equations designed to estimate body size in other taxa, resulting in other extinct thylacinids potentially having their mass overestimated.
- Witton and Habib (2010) noted that several studies on large pterosaurs concluded these animals were likely flightless due to improper estimates of size and form, either overestimating body mass (Henderson, 2010; Sato et al., 2009), or assuming unusually low masses a priori (Chatterjee & Templin, 2004; see also discussion in Paul, 1991; Witton, 2008). Either assumption would significantly affect how we view flight in animals.

1. A large number of studies calculate their estimates via simple isometric scaling, often scaling from a single scaling measurement in a single proxy taxon identified a priori (often an inferred close relative or an extant megafaunal analogue). Many of these studies do not report error values or the range of possible sizes (i.e., margins of error) that their methods would allow. This has gotten better in recent years with the widespread adoption of allometric regression equations and volumetric methods (Campione & Evans, 2020; Damuth & MacFadden, 1990), especially for tetrapods. Studies on fishes and invertebrates, however, still often rely on simple scaling from a single model taxon (Braddy et al., 2007; Jeffery, 1998; Larson, 2010; Young et al., 2013). In rare cases (mostly extremely old historical estimates) methodology for how sizes were calculated is not detailed at all.
2. Initial studies producing size estimates are often very cautious in their estimations (Cox & Hutchinson, 1991; Lambert et al., 2010; Molnar & Vasconcellos, 2016; Newberry, 1873, 1875), often couching their lengths and masses in terms of “this taxon can be shown to have been at least this large” or reporting possible ranges for their size estimates. It is often later citing studies (especially in the grey literature) that remove the nuance from these estimated values due to space limitations and thus report these estimates with much higher confidence than the original authors intended.
3. Initial speculative or overly sensationalized estimates often have a long life span (Black, 2013; Bourke, 2016; Flannery, 2008; Flower, 1955; Greenfield, 2023; Ksepka et al., 2012; Liston, 2003), often continuing to show up in pop culture long after specialists on these taxa recognize them as no longer valid. In popular depictions often the most extreme values, sometimes representing unusually large, “world record” individuals, are frequently cited (Black, 2013; Liston, 2003; Martill & Naish, 2000), which might distort readers’ understanding of the average size of the species.
4. Many size estimates live and die among avocational communities and the gray literature, without ever being considered in the primary literature. Indeed, there are entire online fan communities (carnivora.net, the seemingly now-defunct graphic double integration group on Discord) devoted almost entirely to producing size estimates of extinct taxa, and in some cases these groups are aware of scaling issues before experts even become aware of them. There are potentially several reasons for this. One is that size is a feature of an extinct organism that is readily apparent to most people, and thus people in general are highly interested in it. At the same time, because scaling fossil remains or generating skeletal reconstructions is something that is relatively easy to do without much specialized knowledge or training (the difficult being in knowing which methods are appropriate in what situations), estimating the size of extinct vertebrates is a way that avocational enthusiasts can get involved with studying their favorite extinct species without access to the original fossils or primary literature.

A good example of this is provided by the eugeneodont chondrichthyan *Parahelicoprion*. Lengths of 11–12 m for this taxon are frequently discussed among paleontology enthusiasts, and are treated as if they are common knowledge well-supported by the primary literature. But in fact no size estimate has ever been given for *Parahelicoprion* beyond a mention that this was one of several Paleozoic shark taxa that “attained great size” (Maisey et al., 2017), and in fact most eugeneodont specialists were unaware that general audiences were discussing these 11–12 m estimates until the writing of this article (W. Itano, pers. obs.). It seems as though this estimate was originally produced by an avocational researcher online and was subsequently spread throughout the community until its origin was forgotten and this estimate was mistakenly thought to have come from the primary literature. This is actually a common problem. Avocational researchers will often produce their own estimates for extinct taxa, and unaware audiences will take these “updated” estimates as overriding those in the primary literature (Engelman, pers. obs.). Some of these estimates from avocational researchers may be reasonable, especially if their reasoning is detailed, but because they are not peer-reviewed they are best treated with some skepticism.

1. While many colleagues will express skepticism of existing size estimates in informal conversation or on the Internet, few of these re-evaluations end up being published in the primary literature. This often results in outdated or oversized estimates persisting for years or even decades before being corrected, often only when a study needs accurate estimates of body size/form to work and so requires the most up-to-date values possible. Several possible reasons exist for this, including colleagues not wanting to be drawn into controversy by disagreeing with colleagues’ proposed size estimates unless absolutely certain, but also colleagues considering re-analyzing previously proposed size estimates to not be a productive use of their limited time and resources, unless it directly effects their own topics of interest. This has become less of an issue in recent years as there is now a greater willingness to publish studies primarily focusing on revising body size/form estimates (Bates et al., 2015; Engelman, 2022, 2023a, 2023b; Liston et al., 2013; Millien & Bovy, 2010; Motani & Pyenson, 2024; Romano & Manucci, 2021; Sternes et al., 2024; Young et al., 2016). However, such behavior was much rarer previously (Brown, 1931; Fortelius & Kappelman, 1993; Newman, 1970).
2. Estimates to tend to become more rigorous and better supported over time as more data is collected and our understanding of an extinct organism increases. The scientific process does seem to work; dubious size estimates do get caught and are revised. Nevertheless, this pattern of dubious initial size estimates cannot be attributed to laxer standards for size estimates in the late 19th and early-middle 20th century before quantitative and statistical methods became commonplace in paleontology, as they have occurred in the last ~30 years as well.

# Footnotes and Additional Comments on Size Estimates

1. The largest possible report of an endocerid orthoconid nautiloid was Flower (1955: p. 329), who mentioned “I am not wholly inclined to discredit a report of an endoceroid found in a quarry near Watertown New York, which was measured before it was broken up and found to attain a [conch] length of 30 feet” (9.14 m). However, this report has been treated with considerably skepticism (Pohle & Klug, 2017), not only because Flower (1955) appears to have only heard about the specimen second hand, but also because the specimen was never collected or photographed and was apparently destroyed in the field (Flower, 1955), and thus its existence and size cannot be independently verified. Nevertheless, this report seems to be the basis of depictions of 8–9 m long endocerid nautiloids in popular culture (Falvey et al., 2023; Greenfield, 2023; Marvin & James, 2004).

The largest reliably documented specimen of an early Paleozoic orthocone is a specimen of an endocerid nautiloid held at the Museum of Comparative Zoology (MCZ unnumbered; Pohle & Klug, 2017; Teichert & Kummel, 1960). This specimen has a preserved length of 3 m, but its total conch length is estimated at 5.8 m and soft tissues would have resulted in the animal extending further (Teichert & Kummel, 1960). The conch also appears to be much elongate and narrow than the wide-mouthed cone shown in most popular depictions of endocerid nautiloids, which would result in an overall less massive animal (Greenfield, 2023).

1. Manger et al. (1999) notes that individuals of the middle Carboniferous *Rayonnoceras solidiforme* can reach 2.8 m in conch length. However, they suggest this and several other examples of giant Carboniferous cephalopods should not be taken as typical body sizes for these species, and instead represent “pathological giants”. This is a condition that occurs in some extant mollusks (gastropods) where the animal suffers parasitic castration by trematodes and a result continues to grow throughout its entire life due to a failing to reach sexual maturity. Manger et al. (1999) argue this based on the fact that most of these giant specimens represent singular outliers relative to the “normal” size of the population (rather than a normal growth series) and seemingly show a lack of septal crowding indicating sexual maturity had been reached, contra the condition in Mesozoic cephalopods where conchs of multiple very large, sexually mature individuals are often known.

Klug et al. (2014) challenged this interpretation, noting it could alternatively be due to giant cephalopods exhibiting a type III survivorship curve where very few individuals actually reach sexual maturity. They also note that for Devonian cephalopods, at least, large individuals of these species show septal crowding indicating they were not pathological giants. Klug et al. (2014) notes that it is effectively impossible to “prove” these hypotheses one way or the other, due to the inability to test the sterility, hormone levels, or degree of parasite load on fossil specimens under normal circumstances.

1. *Enchoteuthis* (often referred to under the likely junior synonym *Tusoteuthis*) is a large coleoid cephalopod known primarily from the late Cretaceous of the Western Interior Seaway. Unfortunately, the only parts of this animal that preserve are its gladius (i.e., it's internal pen), making reconstruction of its size and anatomy very difficult. The largest specimen has a near-complete gladius measuring 1.87 m in length (Hoganson, 2014), with other specimens leading authors to propose total lengths of 6–15 m (Hoganson, 2010; Larson, 2010). Larson (2010) claimed a fragmentary specimen of *E. cobbani* had a gladius that if complete would have been 3 m long and pertained to an animal 15 m in total length, but Fuchs et al. (2020: p. 47) revised this estimated gladius length downward to 1.8 m based on comparisons with more complete specimens. This has led to *Enchoteuthis* being reconstructed as a Cretaceous equivalent of a giant squid (*Architeuthis*), with many paleoartistic reconstructions depicting this animal fighting marine reptiles akin to portrayals of *Physeter* and *Architeuthis* (Hoganson, 2010: fig. 1; Larson, 2010: fig. 1).

However, as noted by Fuchs et al. (2020: p. 42, parenthetical comments theirs) these estimates "are based on the 'hope' (i.e. speculation) of very long arms similar to recent *Architeuthis* (arm preservation in the Late Cretaceous of the Western Interior is unlikely but not impossible, in any case still unknown). If arm length were comparable to Jurassic species where arms are preserved, however, these would have been rather short, thus reducing the animal's length significantly." More recent work has highlighted that *Enchoteuthis* is not a decapodiform (true squid) but a muensterelloid octopodiform more closely related to octopodes and vampire squid (Fuchs et al., 2020). As mentioned above, most studies on *Enchoteuthis* have reconstructed incomplete gladii and body shape off of large extant squids like *Architeuthis*, which have elongate rachii, elongate bodies, and extremely long tentacles (Fuchs et al., 2020; Greenfield, 2020b; Hoganson, 2010; Larson, 2010). Reconstructing the body based on other muensterelloids (Fuchs et al., 2020; Greenfield, 2020b) results in a much shorter and stickier body plan where "the entire animal including arms would not have exceeded 3 m in length" (Fuchs et al., 2020: p. 44).

1. Purported gigantic material has since been destroyed or lost
2. When the great appendages of *Anomalocaris* were first recognized as the limbs of a large arthropod, rather than the abdomen of a phyllocarid, Briggs (1972: p. 659) suggested it pertained to an animal at least 1 m in length. They obtained this estimate by examining the appendage length to total length ratio in the myriapod *Arthropleura* and the eurypterids *Carcinosoma* and *Euthycarcinus*. However, when complete remains of *Anomalocaris* were discovered, the entire animal was found to only measure about ~40 cm in length (Whittington & Briggs, 1997). This error is fairly understandable, as nothing akin to the frontal appendages of *Anomalocaris* was otherwise known at the time. An isolated mouth was claimed to represent an anomalocaridid 2 m in total length (Chen et al., 1994: p. 1306–1307), but was subsequently re-evaluated as a lobopodian arthropod (Hou et al., 2006; Vinther et al., 2016).
3. Siluro–Devonian eurypterids such as *Jaekelopterus rhenianae* and *Acutiramus bohemicus* are considered the largest arthropods to have ever existed. However, most of the largest eurypterids are known from fragmentary or incomplete material, requiring scaling from smaller relatives known from complete remains like *Acutiramus macropthalmus* (2.0 m) and *Pterygotus anglicus* (~1.75 m). Braddy et al. (2007) described a giant claw from the eurypterid *J. rhenianae*, estimated at 2.46 m (2.33–2.59 m) long based on simple scaling from *A. macropthalmus* and *P. anglicus*. They also suggested previously reported lengths for *A. bohemicus* (2.5 m; Chlupáĉ, 1994) were overestimates, providing an estimate of 2.1 m based on scaling from a composite specimen of *A. macropthalmus*.

However, Kaiser and Klok (2008) expressed skepticism about these estimates, noting that Braddy et al. (2007) described several features suggesting the chelicerae of pterygotids scaled with positive allometry yet still assumed the chelicerae to scale isometrically. To our knowledge, no response was ever published. Braddy (2023) increased the estimated length of *J. rhenianae* to 2.59 m by simple scaling from *Pterygotus*, based on new findings suggesting *J. rhenianae* is more closely related to this taxon than *Acutiramus*. The question of whether chelicerae scaled according to isometry or positive allometry remained unaddressed. Indeed, if the chelicerae of eurypterids scaled with positive allometry then scaling from *Pterygotus* would be expected to produce larger sizes, since *Pterygotus* is generally smaller than *Acutiramus* and thus the biasing effects of allometry would be more extreme*.*

1. The giant Carboniferous (late Mississippian-early Pennsylvanian) myriapod *Arthropleura* has been estimated to measure 2.0–2.63 m in total length, based on isometrically scaling smaller (< 90 cm), more complete juvenile individuals to the size of trackways and partial exuviae (Davies et al., 2022). Estimated total length/maximum body width ratios for *Arthropleura* range from 3.47 to 4.78 (see discussion in Davies et al., 2022). Despite this, length estimates have been relatively consistent, though this may be due to the paucity of available data and nearly all authors using the same anatomical proxy.
2. Large Permo-Carboniferous griffinflies like *Meganeura* and *Meganeuropsis* are often described as reaching weights of 200–300 g, “the size of a dove” (Anonymous, 2020; Clapham & Karr, 2012), and having wingspans “the size of a seagull” (Benton & Harper, 2009; McGhee, 2018; Ward, 2006: p. 114). These values are sometimes conflated with authors treating these animals as comparable in overall size (i.e., implied mass) to a seagull (Benton & Harper, 2009; McGhee, 2018; Ward, 2006: p. 114), rather than merely having a similar wingspan. This is an issue because while griffinflies may have had wingspans comparable to a seagull, the narrow, cylindrical bodyplans of these taxa mean even the highest estimated weights for these animals (~200 g) are one-fourth or less the weight of a typical seagull (800–900 g; Harris, 1964). While the wingspans of griffinflies were undoubtedly large (estimated 71 cm in the largest specimens of Meganeuropsis; Grimaldi & Engel, 2005), their actual mass (and thus size) has been the subject of some controversy.

Body mass of griffinflies has typically been estimated via several methods, including estimating thoracic dimensions based on scaling relationships (usually wing dimensions) of extant dragonflies and then volumetrically modelling the thorax as a cylinder or estimating the maximum mass that could be carried by the flight muscles. These methods produce a very wide range of possible masses, scaling from extant dragonflies produces masses in the range of 90–210 g (Cannell, 2018; Ellers et al., 2024), whereas studies estimating from thoracic flight muscle mass tend to produce lower mass estimates of 17.8 g (May, 1982) or 34 g (Dorrington, 2016). The latter mass estimates are probably too low (Cannell, 2018), with the most recent studies proposing masses for large griffinflies of 100–140 g (Cannell, 2018; Ellers et al., 2024), still significantly lower than is often cited.

There are several possible reasons for these discrepancy in size estimates. Some of the problem is extinct griffinflies are significantly larger than any living dragonfly, which increases the risk of extrapolation error dramatically. The primary factor driving this uncertainty in the size of extinct griffinflies is that while large wing fossils are known for many taxa, few specimens (and none with wingspans larger than 40 cm) preserve the wings and body in association (Anonymous, 2020), making it difficult to determine the size of the wings relative to the body. Indeed, body shape and mass reconstructions in griffinflies are limited to two taxa (Cannell, 2018; Dorrington, 2016; Ellers et al., 2024; May, 1982): *Namurotypus sippeli* and *Meganeura moneyi* — which are some of the only taxa for which complete body form is known. When complete specimens of griffinflies are known, they invariably show proportionally larger wingspans relative to body length (Anonymous, 2020), though body volume-to-wingspan ratios in griffinflies apparently scale similar to dragonflies (Cannell, 2018). Indeed, wing size would expected to scale with positive allometry in griffinflies, because body mass is proportional to total wing surface area and thus scales according to the square-cube law (Anonymous, 2020). Further investigation of griffinfly proportions is needed to determine how their proportions scale relative to extant odonates and which, if any, of these scaling proposals is correct.

1. See main manuscript text.
2. *Titanichthys* is one of the largest (probably the largest) known placoderm taxon, being slightly larger than *Dunkleosteus terrelli*. Like *Dunkleosteus terrelli*, *Titanichthys* has been suggested to reach lengths of 7–9 m (Hansen, 1996; Parker & Haswell, 1967), seemingly based on comparison with *D. terrelli*. However, rigorous estimation of this taxon’s body size in the literature is rare. Dean (1909) produces a length of ~5 m based on scaling from the proportions of *Coccosteus cuspidatus*, but does not detail which elements were scaled to produce it (infragnathal length is the most likely option). Engelman (2023a) estimates body lengths of 3.34–4.15 m for *Titanichthys* using OOL, but cautions that these values are very preliminary and the size of *Titanichthys* needs to be investigated in more detail due to OOL producing overestimates in large planktonivores. Nevertheless, as with *Dunkleosteus* estimates > 5 m for this taxon are unlikely.

1. Pimiento et al. (2024) report *Glyptaspis verrucosa* to be the largest known arthrodire, with an estimated length of 8 m, for which these authors cite Sallan and Galimberti (2015). However, Sallan and Galimberti (2015) do not provide any information as to how they produced this length estimate (see p. 118 of the supplementary information in that study). The only information Sallan and Galimberti (2015) provide is that this estimate supposedly comes from Denison (1978), but Denison (1978) provides no size estimate in their discussion of *Glyptaspis* (Denison, 1978: p. 105). *Glyptaspis verrucosa* is a very rare arthrodire, only being known from four ventral shield plates (AMNH 92G–95G; Boylan & Murphy, 1978; Newberry, 1889). The total estimated length of the ventral shield of *G. verrucosa* based on these specimens is approximately 71 cm (Boylan & Murphy, 1978: fig. 4). This is significantly smaller than specimens of *Dunkleosteus terrelli*, which frequently have ventral shields measuring 90-100 cm in length (Engelman, In Press). In complete eubrachythoracid arthrodires, the ventral shield length is consistently around ~30–32% total length regardless of phylogenetic position (Engelman, In Press); applying this relationship to *G. verrucosa* produces a total length of 2.2–2.3 m.
2. The case of *Parahelicoprion* is very odd. Among the avocational community this taxon is often cited as the largest known Paleozoic vertebrate, with offered lengths of 12–13 m. However, there has been almost no discussion of the size of this animal in the primary literature (W. Itano, pers. obs.). The only mention of the size of is by Maisey et al. (2017), who lists it among the largest Paleozoic chondrichthyans but also notes there is no empirical way to directly estimate the size of members of its group (Edestoidea). This estimate of 12–13 m for *Parahelicoprion* seems to have originated within the lay community, and more specifically seem to have originated by treating the holotype of *Parahelicoprion mariosuarezi* (Merino-Rodo & Janvier, 1986) as a fragment of a much larger helical tooth whorl. However, there is no guarantee the tooth whorl was helical, and indeed given the proposed similarities between *Sarcoprion* and *Parahelicoprion* (Merino-Rodo & Janvier, 1986) a shorter, more *Sarcoprion* tooth whorl seems more likely. If a *Sarcoprion*-like arrangement were inferred, *Parahelicoprion* likely reached similar sizes to *Helicoprion* (~7 m?).
3. *Orodus* is known from a large number of species, with only two species (*O. greggi* and *O. micropterygius*) being known from body fossils. The holotype of *O. greggi* is incomplete but estimated to measure 4 m long (no reasoning given), whereas the holotype of *O. micropterygius* is about a meter long (Zangerl, 1981). These complete specimens show very small teeth, which if isometrically scaled to the size of the largest known known specimens would produce lengths of 12–15 m (Zangerl, 1981: p. 91). However, Zangerl (1981: p. 91) considers these lengths to “not seem reasonable”, as endoskeletal elements pertaining to chondrichthyans of this size are completely unknown from Carboniferous fossil localities preserving chondrichthyan cartilages and skeletons, such as the Black Mecca Shale of Indiana, USA. Allometry in tooth size across ontogeny may be a more likely explanation. This exacerbated by the possibility that “*Orodus*” sensu lato may be a wastebasket assemblage of unrelated durophagous sharks (Elliott et al., 2004), and thus there is no guarantee of uniform tooth scaling in members of this “genus”.
4. Undescribed material of *Saivodus striatus* from Mammoth Cave National Park has been suggested to belong to individuals 4–7 m long, based on spectacularly preserved cranial cartilages including an incomplete Meckel’s cartilage estimated to measure 60–61 cm long when complete (Hodnett et al., 2023; Hodnett et al., 2024; Hodnett et al., 2022). However, the methods used to produce these estimates have not been detailed, and a more comprehensive assessment of the size of *S. striatus* awaits the full description of the Mammoth Cave material.

1. Ginter (2010) suggested that the latest Devonian *Ctenacanthus tumidus* could reach sizes of 5 m, based on a specimen preserving jaw cartilages in the Cleveland Museum of Natural History (CMNH 5238). How he calculated this estimate was never detailed, though it is possible his estimate was based on Meckel’s cartilage length in neoselachians (Engelman, pers. obs.). Long (2024) posited similar sizes of 5–6 m for Devonian *Ctenacanthus* based on the same specimen, also seemingly based on Meckel’s cartilage length. Engelman (2023a: p. 42) re-examined the total length of CMNH 5238 by isometrically scaling from the Meckel’s cartilage length of this specimen (in which it is 68 cm long) and the complete ctenacanths *Dracopristis hoffmanorum* (Hodnett et al., 2021) and *Goodrichthyes eskdalensis* (Maisey et al., 2017: fig. 5), in which the jaw cartilages are ~16% total length. Scaling from either taxon produced length estimates of approximately 4.2 m. CMNH 5238 is the largest ctenacanth specimen known from the Cleveland Shale; the largest Devonian ctenacanth material at other localities appears to be similar in size to this specimen (M. Greif pers. comm. 2022).
2. Based on very fragmentary material often preserving a limited number of useable size-informative characters.
3. The “Texas Supershark” refers to two fragmentary braincases preserving only the occipital region (AMNH FF 20472 and AMNH FF 20577) collected from the Pennsylvanian Finis Shale of north Texas (Maisey et al., 2017). Maisey et al. (2017) estimated the total length of these individuals by estimating the total length of the braincase by scaling it against the braincase of the holotype of *Tamiobatis vetustus*, and then using the braincase-to-total-length ratio in *Goodrichthyes eksdalensis* and *Dracopristis hoffmanorum* (= “the Kinney ctenacanth in this study) to estimate total length. Based on this, they estimated AMNH FF 20577 to represent an individual 3.39–6.48 m in total length and AMNH FF 20472 represented an individual 4.82–6.82 m in total length. However, Maisey et al. (2017) calculated these estimates using a preliminary report of a 2.5 m total length m for *Dracopristis* (which would make the braincase 10% of total length), when the specimen itself is 2.06 m long (Hodnett et al., 2021), making the braincase about 11.9% total length. Similarly, the braincase of the holotype of *Goodrichthys* (NMS 1950.38.46) as delineated by the figures in Maisey et al. (2017: fig. 5) suggest the braincase is slightly larger than 10% of the specimen’s total length (about 12.5% the total length of the specimen). This suggests the size of the “Texas Supershark” specimens may require further analysis.
4. The body size of *Onchopristis* is an odd case. In this situation the oversized estimates of this species do not appear to have arisen as a result of the primary literature, but as the result of an estimate presented in *Planet Dinosaur* (2011). In this documentary and the companion book, *Onchopristis* was presented as reaching lengths of 8–10 m long, and further claimed to be larger than the largest extant sawfish (Greenfield, 2020a). Because of its presentation as a contemporary and prey item of *Spinosaurus*, this led *Onchopristis* to see a surge in popularity among the paleontology lay community and gray literature, with most sources repeating the 8–10 m estimate. More robust attempts at estimating the size of *Onchopristis* have produced a length of 4 m (Greenfield, 2020a; Villalobos-Segura et al., 2021). While an 8 m *Onchopristis* does not seem to have ever been regarded as reasonable in the formal literature, its depiction in *Planet Dinosaur* has led general audiences to regard this value as a well-supported estimate. Therefore, while *Onchopristis* is technically not an example of size controversies in the primary literature, we do note it as an example of how popular documentaries can distort the public's perception of the size of ancient life (see, e.g., *Liopleurodon*).
5. The late Jurassic pachycormid *Leedsichthys problematicus* is one of the largest bony fishes to have ever existed. Unfortunately, the sheer size of this taxon and the fact that most of its skeleton is unossified mean that its size has been very difficult to determine (Liston et al., 2013). Initial estimates by Woodward (1905, 1917) proposed lengths of 9–10 m based on scaling the tail fin span of the holotype with much smaller pachycormid fishes. Martill (1986) calculated several lengths spanning 13.5–27.6 m by isometrically scaling from a ~2 m individual of the pachycormid *Martillichthys renwickae* (which was unnamed at the time of the study; Liston et al., 2013). Martill (1986, 1988) regarded the largest of these estimates to be the most plausible, which led to a variety of 70–90 foot (22–27 m) depictions of *Leedsichthys* in popular media, many of which compared this species in size to a blue whale (*Balaenoptera musculus*) (Black, 2013; Liston, 2003).

Liston et al. (2013) returned to these size estimates following the discovery of more complete *Leedsichthys* material (PETMG F174, the “Ariston” specimen). They produced estimates ranging from 8.0–16.5 m for four individuals of *L. problematicus* by scaling from a number of pachycormiform taxa, primarily *Saurostomus esocinus* due to identifying it as a better anatomical proxy. They noted that the very largest estimates of 27.6 m for *L. problematicus* in Martill (1986) came from isometrically scaling the width of the gill chamber, yet this morphological proxy likely scaled positively with body size due to the square-cube law and planktonivorous fishes tend to have disproportionately large gill chambers.

1. In a survey of what are often very sketchy size estimates, we would like to highlight the length estimate of 2.29 m for *Oncorhynchus rastrosus* in Stearley and Smith (2016) as an exemplar of what should be done when estimating the size of extinct organisms. Stearley and Smith (2016) note that large vertebrae of *O. rastrosus* have a centrum length of 2.8 cm. Given that extant relatives (sockeye salmon, *O. nerka*) tend to have precaudal vertebral counts of 56–67 vertebrae, they use a midpoint vertebral count of 62 to produce an estimated precaudal vertebral column length of 173.6 cm. They then multiply this by 1.32, which results in a precaudal (standard) length of 2.29 m. The weight of this species is then estimated using established length-weight equations for salmonid fishes.

In this case, the exact steps used to produce the estimate are clearly laid out, the assumptions made are explicit, and the math used can be clearly followed and replicated. The only thing we might criticize is the basis for assuming the head is 32% of standard length is never explicitly given. New material of *O. rastrosus* with associated cranial and postcranial remains may help give a firmer answer on head-trunk proportions in this species (Claeson et al., 2024).

1. *Rhizodus hibberti* is known from mandibles nearly a meter long (e.g., NMS G 1847.48.5, mandible length 89 cm; Jeffery, 1998), which has led some authors to suggest lengths of 6–7 meters for this species (Andrews, 1985; Johanson & Ahlberg, 2001; Zhu et al., 2017). However, scaling from other sarcopterygians known from complete remains, including the tristichopterid *Eusthenopteron* and the rhizodonts *Strepsodus* and *Goolongongia*, produces length estimates ranging from 4.54–5.6 m (Engelman, 2023a; Jeffery, 1998), with estimates of 5.0–5.6 being produced by scaling from smaller, more complete rhizodonts.
2. *Hyneria* is often presented as a massive predatory sarcopterygian, reaching lengths of 5 m. This portrayal seems to stem directly from *Walking with Monsters*, where it is described as “weighing two tons, and five meters long” (Evans et al., 2005). While it is tempting to attribute this to the *Walking With* series’ tendency to present unusually large sizes for their depicted species (viz. *Liopleurodon*, *Leedsichthys*, *Ornithocheirus*; Liston, 2003), it seems more likely this was related to a misunderstanding related to the largest specimen of *Hyneria lindae*, which is a fragment of dentary and jaw symphysis. It is also possible this is due to confusing *H. lindae* with a rhizodont, which do reach 5 m in length and have been proposed to exhibit behavior more similar to *Walking with Monsters*’ depiction of *Hyneria* (Jeffery, 1998). Gigantic predatory sarcopterygians, including this fragment of a giant *H. lindae*, have been consistently calculated to measure closer to 2.7–3.6 m long based on scaling from smaller relatives (Engelman, 2023a; Gess & Ahlberg, 2023; Young et al., 2013).
3. *Mawsonia* has often been claimed to reach lengths of 5–6 m long, based on isometric scaling from extant *Latimeria* and other coelacanths (Toriño et al., 2024). However, mawsoniids show positive allometry in jaw length and have proportionally larger heads than other coelacanths, which may be due to differences in paleoecology (Toriño et al., 2024). Methods for estimating the body size of coelacanths based on gular plate length overestimate the size of complete mawsoniids by nearly 10% (1.55 estimated versus 1.43 actual), suggesting traditionally proposed lengths of 5–6 m may be overestimates.
4. The archegosaurid temnospondyl *Prionosuchus* has been proposed to be the largest known amphibian and the largest known Paleozoic tetrapod, with lengths of 9 m for this species frequently cited (Levy & Heald, 2015). This value seems to have been calculated by isometrically scaling the largest fragment of a *Prionosuchus* rostrum to smaller, more complete skull material (producing an estimated skull length 1600 cm long) and then assuming similar head-body proportions as the modern gharial (*Gavialis gangeticus*), the largest of which the original description says has a skull 83 cm long and a total length of 5.5 m (Cox & Hutchinson, 1991). It is rather unusual the authors chose this method of estimating the size of *Prionosuchus*, when they mention the specimen also preserves a fragments of the vertebrae, ribs, scapulae, cleithra, clavicles, ilium, ischium, and femur (Cox & Hutchinson, 1991). The caveat here is that there is no guarantee that head size scales isometrically in *Prionosuchus*, or that head-body proportions for a modern gharial are similar to that of *Prionosuchus*. Notably Cox and Hutchinson (1991) do not explicitly consider *Prionosuchus* to be 9 m long, explicitly stating it is unclear if the head-body proportions of the two are comparable, but merely state it was clear the largest individuals of *Prionosuchus* were larger than 5.5 m gharials. The 9 m estimate seems to be from taking their scaling at face value, though the authors who were the first to do this are unclear. Lengths of 9 m for the largest individuals of *Prionosuchus* are clearly overestimates but a more comprehensive analysis of this taxon’s body size awaits further analysis of undescribed material (J. Pardo and J. Cisneros, pers. comm. 2024).
5. The “Precious of Lesotho” is a colloquial name given to a large maxilla fragment (MNHN L1970) from the Late Triassic or Early Jurassic of South Africa (Damiani & Steyer, 2005). This fragment is about 22 cm along its greatest length and preserves 13 tooth loci, overall representing a very small portion of the skull (Damiani & Steyer, 2005: fig. 2b). MNHN L1970 was originally interpreted as belonging to a mastodontosaurid, but was later re-interpreted as belonging to a brachyopoid temnospondyl. This greatly increased the inferred size of the animal, suggesting a skull in excess of 1 m in length (Damiani & Steyer, 2005). Damiani and Steyer (2005) estimated the total length of MNHN L1970 by scaling the preserved maxilla fragment against the more complete skull and complete skeletal reconstruction of *Siderops kehli* (Warren & Hutchinson, 1983), producing an estimated skull width of 1.7 m and an estimated total length of 7 m (Damiani & Steyer, 2005: p. 246–247), leading the authors to conclude it was potentially the largest amphibian (sensu lato) to ever live (the length of *Prionosuchus*, for which similar claims have been made, was not discussed; Cox & Hutchinson, 1991).
6. Typical individuals of the large, latest Cretaceous (Maastrictian) ceratophryid frog *Beelzebufo ampinga* were originally reported to have snout-vent lengths (SVL) of 16–27 cm (Evans et al., 2008). However, a few specimens were interpreted as pertaining much larger inferred female individuals with an estimates SVL of 42.5 cm (Evans et al., 2008). This would potentially make *B. ampinga* one of the largest known anurans to ever exist, similar in size to the extant goliath frog (*Conrau goliath*), the largest living anuran. However, more complete specimens of *B. ampinga* show these values are likely overestimates (Evans et al., 2014: p. 49), with the largest individuals suggested to have SVLs of 23.2 cm, though the authors note the possibility for further growth. This is still relatively large for an anuran, about the size of a cane toad (*Rhinella marina*) and larger than any extant ceratophryid, but not as large as originally proposed.
7. Reported sizes of the giant Pleistocene monitor lizard megalania (*Varanus priscus*) have greatly varied in the literature, ranging from 9.1 m at the largest (Lydekker, 1888) to 3.45 m (with a range of 2.20–4.67 m) at the lowest (Wroe, 2002). Part of the issue is no known individual of megalania is even 50% complete (Molnar, 2004), leaving researchers to try and reconstruct Most of these estimates are between 4.5 and 7.5 m total length, except for Lydekker (1888). (Molnar, 2004: p. 110) suggests the reason Lydekker (1888)'s estimate was so anomalously large is they may have estimated the size of megalania based on a crocodile monitor (*Varanus salvadorii*), as Komodo dragons were not known at the time of their study. *V. salvadorii* has one of the longest tails relative to SVL among extant monitors, which would potentially result in an unusually high total length for megalania.

The two most extensive attempts to estimate the size of megalania have been by Hecht (1975) and Molnar (2004). Hecht (1975) estimated the size of megalania based on simple scaling from a Komodo dragon (specimen and size unknown). He obtained snout-vent length estimates ranging from 1.4–5.0 m for a number of individuals. However his largest length estimate, based on an ungual considered to belong to an individual with a snout-vent length of 4–5 m, was based on material that does not appear to pertain to megalania (Molnar, 2004). Molnar (2004) based his size estimates of megalania on simple scaling from a 1.24 m total length individual of a lace monitor (QM J16156, *Varanus varius*), and obtained roughly similar estimates (~5–10% difference; Molnar, 2004).

The largest currently known individual of megalania is represented by a dorsal vertebra (QM F2942) estimated to have come from an individual with a snout-vent length of 3.8 m (Hecht, 1975; Molnar, 2004). Assuming a tail that is around half total length, as is the case for most large, ground-dwelling varanids (Molnar, 2004), produces a length of 6.9 m. However, if assuming a tail as in some arboreal varanids like *V. varius*, megalania could have potentially reached lengths of 7.9 m. Molnar (2004: p. 117) considers cited lengths ≥ 8 m total length for megalania to be overestimates.

Weight estimates for megalania have also been subject to a great deal of uncertainty. Since estimates of weight depend on estimated length, we do not go into detail here but refer to the reader to Molnar (2004: p. 119–127) for a detailed summary for the history of weight estimates and potential statistical issues.

1. Head et al. (2013) estimated the size of *Barbaturex morrisoni* based on a regression equation of dental length against snout-vent length in extant agamids and chamaeleonids. This produced an SVL of 100 cm, potentially correlating to an animal 1.8 m in total length (Head, 2013). These choices seem reasonable, though we note the possibility for extrapolation error given the estimated SVL of *Barbaturex* is nearly five times the size of the largest individual in the extant dataset (a 20.4 cm SVL individual of *U. maliensis*).
2. *Mosasaurus hoffmanni*, the largest species of mosasaur, has sometimes been estimated as reaching 15–17 m in length (Everhart et al., 2016; Grigoriev, 2014; Lingham-Soliar, 1995; Russell, 1967). This estimate originally seems to derive from Russell (1967: p. 210), who scaled the size of this species assuming a head-body ratio of 1:10. Exactly why a 1:10 ratio was chosen was not detailed but may be from assuming similar proportions to tylosaurine mosasaurs, though of the three complete mosasaur taxa discussed by Russell (1967) two (*Clidastes* and *Tylosaurus*) had heads that were ~13% total length (head-body ratio of 1:7.75), only *Platycarpus* has a head-body ratio of 1:10. Fanti et al. (2014) suggested lengths of 15–17 m for *Mosasaurus* are probably overestimates, as the more closely related mosasaurine mosasaur *Prognathodon overtoni* shows a head-body ratio of 1:7 (Konishi et al., 2011). This would suggest lengths of 11–12 m for *M. hoffmanni*. Other authors have expressed skepticism of the larger 15–17 m estimates (Cleary et al., 2018).

Mosasaur research makes almost no use of regression equations when predicting body size (Christiansen & Bonde, 2002; Driscoll et al., 2019; Everhart et al., 2016; Fanti et al., 2014; Lingham-Soliar, 1995; Polcyn et al., 2014), instead almost exclusively relying on simple isometric scaling ratios derived from a priori anatomical and taxonomic proxies (often following the proportions of Russell, 1967: table 2, or assuming a 1:10 head-body proportion). This makes mosasaur size estimate particularly vulnerable to issues of positive/negative allometry and questions of intra/interspecific variation in body proportions (see Grillo & Delcourt, 2017 for an analogous discussion in abelisauroids). Indeed, while the assumption of a 1:10 head-body ratio seems common in mosasaur research, most mosasaurs seem to have head-body proportions closer to 1:7 or 1:8 than 1:10 (Christiansen & Bonde, 2002; Konishi et al., 2011). Depending on how widespread estimates assuming a 1:10 ratio have been made, the size of other mosasaur taxa may need to be reevaluated.

1. The large late Cenozoic side-necked turtle *Stupendemys geographicus* was originally described based on several specimens including a near-complete carapace measuring 2.18 m in length (MCZ 4376; Wood, 1976). Because body size in turtles is typically reported in terms of carapace length, it is extremely difficult to distort the size of a taxon if a complete carapace is known. Larger complete carapaces have since been described, including one (CIAAP-2002-01l) with a carapace length of 2.86 m (Cadena et al., 2020).
2. *Megalochelys atlas* (sometimes referred to as *Colossochelys atlas* or *Megalochelys sivalensis*) is an extremely large tortoise from the late Cenozoic of India. The original describers of this taxon (Falconer & Cautley, 1837, 1844) estimated the carapace of this taxon at 3.73 m in straight-line length (4.8 m curved carapace length) based on a composite specimen and repeatedly compared it in size to an Indian rhinoceros (*Rhinoceros unicornis*). Weight estimates of ~4000 kg are frequently cited in the gray and popular literature, which appear to be derived from a misinterpretation of Falconer and Cautley (1844) 's results passed on in "a game of academic telephone" (Bourke, 2016).

However, it appears Falconer and Cautley (1844) 's size estimate was an overestimate due to the use of a composite specimen. This was suggested by Lydekker (1889), who further suggested the actual length of the specimen was only about 1.8 m long. This was further supported by Brown (1931), who found a second carapace about the same size as the material described by Falconer and Cautley (1844), which measured only about 2.2 m in curved carapace length. Brown (1931) attempted to estimate the weight of *M. atlas* by measuring the volumetric displacement of a scale model, which produced an estimate of 955 kg. Scaling this specimen isometrically from a Galapagos tortoise produces similar results (929 kg; Bourke, 2016). Hirayama et al. (2015) claim to have found material of *Megalochelys* pertaining to an animal with an estimated carapace length of 2.7 m, but this material has only been mentioned in an unpublished preprint.

1. *Shonisaurus popularis* is a very large ichthyosaur known from the late Triassic of Nevada. Camp (1980) posthumously provided a monographic treatment of this taxon, including a reconstruction and estimate of body size. In reviewing the articulated but incomplete material from the site, they report “lengths in excess of 12 m are very probable, those of more than 13.7 m are possible, and it is unlikely that any of the ichthyosaurs exceeded 15.2 m in total length (Camp, 1980: p. 190). Camp (1980: p. 190) also notes that the size of *S. popularis* was been previously estimated as 12.2–18.3 m, but estimates exceeding 15 m were calculated based on the length of loose, disarticulated vertebral series in situ. Camp (1980) does not provide a citation for where these 15–18 m estimates came from, and his initial description of *S. popularis* makes no mention of estimates this large (Camp, 1976).

Camp (1980)’s reconstruction has a number of unusual proportions, including a very deep trunk and large head. It is possible the unusual nature of this reconstruction is due to Camp passing away before his research could be completed, as Camp made mention in both his work and his field notes that the reconstruction needed adjustment (Kosch, 1990). Kosch (1990) revisited the body form of *Shonisaurus* and found a number of discrepancies between the reconstruction of Camp (1980) and the original fossils and Camp (1980)’s field notes, including the skull being too large relative to the body, the tail being too long, the ribs being too vertical, and the dorsal series potentially being too short by 1–2 m. Surprisingly, these changes in body form seem to have little effect on overall size; Camp (1980) estimates the length of the figured individual at 14 m whereas Kosch (1990) estimates it at 13.6 m. Both Camp (1980) and Kosch (1990) state that isolated remains suggest *Shonisaurus* could reach lengths of 15 m. Camp (1980) based this on an isolated radiale 20% larger than the complete specimen of *Shonisaurus*, what Kosch (1990) based their statement on is unclear but it could have been the same material.

McGowan and Motani (1999) revisited *Shonisaurus* and reported an estimated size of 14.4 m for Specimen D of Camp (1980). It is not entirely clear if their estimate represents an increase or decrease relative to Camp (1980) and Kosch (1990) because this length estimate was calculated using different individuals. They write “[t]he length of the vertebral column, as preserved, is 8.7 m and we estimate that the complete column would have been about 12 m long” (McGowan & Motani, 1999: p. 46). How they estimated the missing length of the vertebral column was not detailed. McGowan and Motani (1999: p. 47) also note that some isolated material pertains to larger individuals than any of the partially articulated specimens, and reiterated that this material appears to pertain to individuals about ~15.5 m long.

1. *Shastasaurus sikanniensis* has been reported to be the largest known marine reptile, with a total length of 21 m (Nicholls & Manabe, 2004). This is largely based on a partially articulated skeleton collected from the late Triassic of British Columbia. However, several paleoartists have mentioned being unable to replicate this 21 m estimate when trying to reconstruct the skeleton of *Shastasaurus sikanniensis* using the figures in Nicholls and Manabe (2004) (Paul, 2022; 2024; R. Hawley pers. comm. 2022). Part of the issue is that while the skeleton is partially articulated and reported to be 21 m long “as measured in the ground” (Nicholls & Manabe, 2004: p. 839) the truth is more complicated. The skull, cervical series, proximal pectoral elements, and anterior trunk are articulated, but these elements are separated from the articulated caudals by a 4 m gap, suggested to be the result of erosion by the river (Nicholls & Manabe, 2004: p. 839). The specimen is similarly missing the rostrum anterior to the nares. No pelvic girdle elements were preserved in situ that might allow determination of the intergirdle distance and thus better constrain total length.

Paul (2024) suggests some of this discrepancy may be due to a lapsus in interpreting the quarry map. The quarry map starts at 4 m rather than 0 m (Nicholls & Manabe, 2004: fig. 2), meaning the 21 m estimate is only possible if assuming the missing rostral (pre-narial) portion of the skull measured over 4 m in length. This might not be out of the question if assuming a *Shonisaurus*-like skull (Camp, 1980), which would be reasonable at the time of its initial description because *S. sikanniensis* was originally referred to *Shonisaurus* (Nicholls & Manabe, 2004), but subsequent research has since suggested *S. sikanniensis* is more closely related to *Shastasaurus* (Sander et al., 2011), which has been noted to have a very short snout among ichthyosaurs (Sander et al., 2011). *Shastasaurus sikanniensis* probably is the largest well-preserved marine reptile, but more evidence is probably needed to better test the originally reported 21 m length.

1. *Ichthyotitan* *severnensis* is a large, latest Triassic ichthyosaur known from southwestern Europe. The holotype is only known from an isolated surangular (Lomax et al., 2024), though additional material (the "Aust Colossus") may pertain to the same or a closely related species (Lomax et al., 2018). Lomax et al. (2024) calculated the length of *Ichthyotitan* by simple scaling of the height of the surangular in *Besanosaurus leptorhynchus* at the level of the process for the *m. adductor mandibulae externus* and the distance between this process and the coronoid process in *Opthalmosaurus icenicus*. No attempt was made to control for allometry and the only reason these landmarks were used for scaling is they were some of the few preserved in material of *Ichthyotitan*. This produces length estimates of ~25 m for *I. severnensis*, with “very speculative” lengths of 30+ m for a possible surangular in the Aust material, comparable in size to a blue whale (Lomax et al., 2024). However, other researchers have expressed skepticism of these extreme sizes (Paul, 2024) though no formal challenge of Lomax et al. (2018, 2024)’s size estimates has yet been published in the primary literature. The holotype surangular of *Ichthyotitan* does appear unusually small when compared to analogous regions (the mandibular condyle and attachment sites for the jaw adductors) in the mandible of whales similar to the proposed size for *Ichthyotitan* (*Physeter*, Mysticeti), which would be very unusual for an organism touted to be "approaching the size of a blue whale". However, the authors lack the familiarity with ichthyosaur anatomy necessary to speak confidently about whether this estimate is reliable.

The broader issue is that Lomax et al. (2024) never demonstrated the distance between the coronoid and process for the *m. adductor mandibulae externus* scales consistently with body size in ichthyosaurs, much less that it scales in a simple isometric manner as implicitly assumed by using simple scaling methods. This measurement is expected to correlate with head size, and given head-body proportions in ichthyosaurs are already known to be highly variable (Kosch, 1990; Sander et al., 2011), the ability of this measurement to predict total length might be expected to be low. Similarly, given that both of these processes relate to jaw muscle attachments, their position and morphology might be more influenced by functional morphology than overall size, and thus prediction accuracy would be low. At this point we do not know how reliable these measurements correlate with body size.

1. Late Jurassic-Early Cretaceous pliosaurs, such as *Megalneusaurus*, *Liopleurodon*, *Kronosaurus* (but see Noè & Gómez-Pérez, 2022), and *Pliosaurus* were some of the largest marine predators in their ecosystems. Pliosaurs were known to have reached gigantic sizes for some time (Tarlo, 1959), however this great size was not really stated in quantitative terms until the reconstruction of the MCZ *Kronosaurus*, which was measured at 12.8 m (McHenry, 2009; Romer & Lewis, 1959). However, much of this specimen is reconstructed with plaster and at least eight additional plaster vertebrae for which there was no evidence added to the mount (McHenry, 2009). Recalculating the length of the MCZ specimen accounting for these factors produces a precaudal length of 7.2–7.5 m and a total length of 10.4–10.9 m (McHenry, 2009). Noè and Gómez-Pérez (2022) estimate the precaudal length of this specimen at 7.9 m based on summing the lengths of the skull and precaudal vertebrae, producing a relatively similar estimate to McHenry (2009: tab. 6-13).

Several other fragmentary pliosaurid remains have been considered to come from larger animals, but in general when revisited produce length estimates in the range of 10–11 m or below (see review in McHenry, 2009). One specimen, the "Monster of Aramberri" (UANL-FCT-R2), was originally reported at 15 m long (Buchy et al., 2003) but appears to have come from an animal ~10.7 m (7.2–12.4) m long (McHenry, 2009). Another specimen, the Petersborough vertebra, was estimated to belong to an individual ~12.6 m in length, but appears to be from a sauropod dinosaur (McHenry, 2009; McHenry, 2020). The "Cumnor mandible" (OUM J.10454) seems to be one of the larger known pliosaur specimens with an estimated length of 11.5–12.7 m (McHenry, 2009), but the specimen itself is heavily restored with wood and plaster and it is unclear how much of it is real (Forrest, 2009).

Similar issues are present in other pliosaurid genera. *Pliosaurus funkei*, originally reported based on the two individuals “Predator X” and “The Monster” (PMO 214.135 and PMO 214.136, which name refers to which specimen is not clear), was originally reported to be 15 m in length or more (Switek, 2012), but the actual description of this taxon suggests it was 10–13 m in length (Knutsen et al., 2012). Pliosaurs, by far, seem to be one of the worst offenders as a group when it comes to dubious size estimates.

Perhaps the most infamous case of oversized body estimates in pliosaurs regards the 25 m long depiction of *Liopleurodon ferox* presented in *Walking with Dinosaurs* (Chambers et al., 1999). From what can be determined (Martill et al., 2023; Martill & Naish, 2000; McHenry, 2004; Naish, 2021; Naish et al., 2001), this reconstruction seems to be based on a number of factors: 1) treating the Petersborough vertebra as pliosaurid, which was assumed to come from an individual 17–20 m in length (Martill & Naish, 2000), and 2) “one of their consultants pushed the extreme size as a scientifically supported, justifiable extrapolation (Naish et al., 2001) and they followed it” (Naish, 2021). Based on available evidence, this consultant appears to be D. Martill (Martill et al., 2023; McHenry, 2004). From available evidence, what it appears happened is the already speculative 17–20 m length was taken as the size of a typical large adult which was then extrapolated to even larger sizes to represent a hypothetical “world-record” individual (Martill & Naish, 2000).

More recently, Martill et al. (2023) reported a series of four partial pliosaurid cervical vertebrae they estimate as having come from an individual 9.8–14.4 m in length, further stating that “likely the true length was towards the higher end of this range”. This estimate was calculated by simple scaling from cervical vertebra length in four pliosaur taxa (*Liopleurodon*, *Peloneustes*, *Sachicasaurus*, and *Stenorhynchosaurus*); it is worth noting that the higher estimates of 14.4 m are only obtained if scaling from *Liopleurodon*, the other taxa produce lengths of 9.8–12.5 m. This estimate has been met with some skepticism, and a recent preprint suggests lengths of 10.7–11.8 m based on comparisons with other pliosaurs (Zhao, 2024).

1. The cretaceous teleosaurid crocodylomorph *Machimosaurus* (specifically the species *M. hugii* and *M. rex*) were originally estimated to measure 9 and 10 m in length, respectively, by isometrically scaling from basicranial length in smaller species of *Machimosaurus* (Fanti et al., 2016; Young et al., 2014). However, Young et al. (2016) showed that compared to extant crocodilians teleosaurids exhibited proportionally larger heads and more pronounced positive allometry in skull length, meaning these values were overestimates. Creating a new allometric regression equation based on teleosaurids, they estimated *M. hughii* to only reach a length of 6.9 m and *M. rex* to only reach 7.15 m.
2. *Barinasuchus arveloi* is a very large, (likely terrestrial) sebecosuchian crocodyliform from the Miocene of northern South America. This species is known from a very large rostral fragment (MAAT-0260) measuring 70 cm in length, or ~50 cm to the end of the dental series (Molnar & Vasconcellos, 2016). Molnar and Vasconcellos (2016) estimate the skull length of this individual to have been 95-110 cm based on scaling from *Baurusuchus*, and the total length of this individual at 6 m (scaling from *Stratiotosuchus maxhechtii*) or 7.5 m (scaling from head-body length in *Crocodylus porosus*. From this they estimate the mass of *B. arveloi* to have been ~1600–1700 kg based on length-weight equations in *Crocodylus porosus*. As noted by the authors, this would make *B. arveloi* the largest known Cenozoic land carnivore.

Molnar and Vasconcellos (2016) do discuss possible limitations and caveats of this estimate. They note “*Barinasuchus* is represented by only three specimens, all cranial, all incomplete, only two of which represent more than about 10% of the skull and those lack the orbital and postorbital regions. Thus the length and mass estimates are necessarily inexact, because an incomplete skull is used to estimate the skull length, from which the total length is then estimated, and from that the mass. Obviously there is the possibility of error accumulating from estimates based on other estimates that may not be entirely accurate. But our argument does not require complete accuracy, only an approximate figure. We seriously doubt that a creature with a snout over 600 mm long was a small animal, less than three metres in length.” (Molnar & Vasconcellos, 2016: p. 372). They also note that the use of *Stratiotosuchus* and *Baurusuchus* is not ideal, given they belong to separate families from *Barinasuchus* (Baurusuchidae versus Sebecidae) and even the monophyly of Sebecosuchia has been debated (Larsson & Sues, 2007; Pol et al., 2012), but such methods were necessary as *Stratiosuchus* is the only sebecosuchian known from complete remains at the time of their study and there is limited postcrania known for Sebecidae (Pol et al., 2012).

1. Sereno and Larsson (2009: p. 64) originally reported the total length of the *Kaprosuchus saharicus* at ~ 6 m. Exactly how this estimate was produced was not detailed. However, the holotype and only known skull of this taxon has a skull length of 50.7 cm and a width across the quadrate condyles of 21.4 cm, which would imply an unusually small head that is only 8.5% total length, which has led to suggestions the size is overestimated (C. Hays, pers. comm. 2023). Testing this suggestion by attempting to replicate the length of *K. saharicus* in Sereno and Larsson (2009) using several different crocodilian-based OLS equations that use both skull length (dorsal cranial length) and head width produce much smaller lengths of 2.42-3.77 m for this taxon (Table S4). These are rather back-of-the-envelope methods (i.e., using OLS versus PGLS), but they support suggestions that lengths of 6 m for *Kaprosuchus* are overestimates.
2. *Aegisuchus witmeri* is an aegyptosuchid crocodyliform known only from an isolated braincase from the middle Cretaceous of Morocco. Holliday and Gardner (2012) estimated the size of this taxon by using the width across the exoccipitals (~23 cm) to obtain an estimated skull length of 2.08–2.86 m, which when applied to regression equations in *Crocodylus porosus* and *Gavialis gangeticus* produced an estimated total length of 15–22 m. However, these estimates were subsequently criticized (Morales-Betancourt, 2014), noting that the provided regression equations failed to replicate the reported skull lengths. Revisiting the original sources of the equations produced skull lengths of 62–90 cm for *A. witmeri*. This resulted in relative snout length proportions similar to *G. gavialis*, leading Morales-Betancourt (2014) to suggest this taxon (and the resulting skull length estimate of 62 cm) is the best approximation of the condition in *A. witmeri*. Applying the *Gavalis*-based regression equation from Sereno et al. (2001) results in a total length of ~3.9 m for *A. witmeri* (Morales-Betancourt, 2014). Holliday and Gardner (2012) themselves suggest lengths of 15 m for *Aegisuchus* are “almost certainly overestimates”, they merely intended to highlight the large size of the *Aegisuchus* holotype braincase.

1. Sereno et al. (2001) estimated the early Cretaceous pholidosaurid crocodyliform *Sarcosuchus imperator* to measure approximately 11–12 m, and then subsequently used this value to estimate a weight of ~8000 kg based on a total length/body mass regression equation in *Crocodylus porosus*. However, other authors have been unable to replicate these values. Farlow et al. (2005) estimated a total length of 72.5 m and a mass of 2411 kg for *S. imperator* based on the femoral circumference of an animal 75% the size of the holotype, which would scale up to a length of 9.11 m and a weight of 3215 kg for the larger individual (O’Brien et al., 2019: p. 10). O’Brien et al. (2019) calculated a total length of either 7.63 m (7.16–8.14 m) or 8.97 (8.49–9.47 m) using head width, depending on the phylogenetic position of *S. imperator*. This, in turn, produced mass estimates of 1925 kg (1493–2503 kg) or 2416 kg (1976–2980 kg), respectively. This was relatively close to the mass estimate produced by Farlow et al. (2005), but significantly smaller than Sereno et al. (2001) in both mass and length. It is unclear why the length estimates of these studies and Sereno et al. (2001) differ so much, though O’Brien et al. (2019) suggested the difference in weights could be due to the extant sample Sereno et al. (2001) used to calculate body mass. *Sarcosuchus imperator* is known from a significant portion of the vertebral column (Sereno et al., 2001), but it is unclear why other studies get such disparate values for estimates of length and mass.
2. The giant alligatoroid crocodylian *Deinosuchus* was originally estimated at "fifty feet" (15.2 m) by Colbert and Bird (1954) based on a skull (AMNH 3073) from the latest Cretaceous of Texas, which subsequently became the standard in the literature (Schwimmer, 2002: p. 44). However, this skull was heavily reconstructed with plaster and subsequent authors have noted that several aspects of its morphology do not resemble well-preserved *Deinosuchus* fossils (Schwimmer, 2002: p. 44–45), making its reliability in size estimates of this taxon unclear. Most recent studies seem to treat the maximal length of *Deinosuchus* at ~10 m (Erickson & Brochu, 1999; Farlow et al., 2005; Schwimmer, 2002). Farlow et al. (2005) estimate the total length of one specimen of *Deinosuchus* (TMM 43632-1) at 10.6 m based on a mandible length of 147.5 cm, this seems to be one of the greatest lengths treated as reliable in the literature. Schwimmer (2002) estimated a possible length of 12 m for two individuals of *D. riograndensis* (including AMNH 3073) based on vertebral central length and simple scaling from other crocodilians; however, Iijima and Kubo (2020) estimated the length of AMNH 3073 using the same methods and a regression equation based on a larger dataset and estimated the size of this specimen at 7.73 (7.10–8.34 m). Claims of larger (13+ m) sizes for *Deinosuchus* spp. have continued to circulate on the Internet, but are generally not considered reliable.
3. During the Miocene epoch, northern South America was inhabited by several species of gigantic crocodylians, including the caimanines *Purussaurus* and *Mourasuchus*. Size estimates for these taxa frequently exceed 9–10 m. Estimates are available for a number of specimens (Table S5): Aureliano et al. (2015) estimate the size of a very large skull of *P. brasiliensis* (UFAC 1403) based on skull length in *Caiman latirostris* (Verdade, 2000). Moreno-Bernal (2007) estimate the length of the same specimen as well as a large specimen of *P. neivensis* (UCMP 39704) using dorsal skull length and the regression models of Woodward et al. (1995) for *Alligator mississippiensis* and Webb and Messel (1978) for *Crocodylus porosus*. Scheyer et al. (2019) estimate of *P. mirandai* (CIAAP-1369) using the PGLS regression of O’Brien et al. (2019). Cidade et al. (2019) estimated the length of specimens of *M. amazonensis* (DGM 526-R), *M. arendsi* (CIIAP 1297), *M. atopus* (UCMP 38012), and *M. pattersoni* (MCNC-PAL-110-72V) using one regression equation from *C. latirostris* (Verdade, 2000) and two from *A. mississippiensis* (Grigg & Kirshner, 2015; Hurlburt et al., 2003).

Paiva et al. (2022) estimated the lengths of the same specimens using a broad sample of crocodilians and both phylogenetically informed and simple OLS regression methods, and generally obtained much smaller lengths (Table S5), with the largest specimen (UFAC 1403, *Purussaurus brasiliensis*) estimated as being ~9-10 m under OLS and only 8 m under PGLS. It is difficult to explain why this discrepancy occurred, as the original estimates were conducted by a number of different research groups and in many cases both they and Paiva et al. (2022) based their estimates on the same specimens. It is possible this result is due to most of these studies using simple linear regressions (OLS) instead of phylogenetically informed ones (PGLS). The smallest discrepancy is in the estimated length of *P. mirandai* (CIAAP 1369) with only an 11% difference in length; this makes sense given Scheyer et al. (2019) and Paiva et al. (2022) both used PGLS methods rather than OLS. Paiva et al. (2022) attribute this discrepancy to the prior studies including juvenile specimens in their regression equations, potentially biasing results. The data of Paiva et al. (2022: fig. 5a, c) also seem to imply dorsal cranial length scales with non-linear allometry in crocodilians (pers. comm. between A. Paiva and R. Engelman, August 2022), which might potentially imply skull length has a tendency to systematically overestimate body size in the largest crocodilians.

One caveat is that the PGLS estimates of Paiva et al. (2022) (which use head width) result in *Mourasuchus* spp. having a head approximately 25% total length. This is an extremely large head relative to body size for a crocodilian, but these results should not be dismissed out of hand. *Mourasuchus* has an extremely long snout that is over 80% total skull length (Cidade et al., 2019: fig. 1), and the smaller estimates in Paiva et al. (2022) might better agree with the very small basicranium. Reconstructing *Mourasuchus* with greater body lengths results in the basicranium and skull table being unusually small.

1. Late Cretaceous azdarchid pterosaurs like *Quetzalcoatlus northropi* were some of the largest animals to have ever flown. However, estimates of size (wingspan and body mass) for these taxa have been variable. This is in large part due to the fragile nature of pterosaur skeletons, meaning that for many of these taxa only isolated and fragmentary bones are preserved, necessitating scaling from smaller, more completely preserved relatives (Andres & Langston, 2021; Buffetaut et al., 2003; Frey & Martill, 1996). For example, the giant *Quetzalcoatlus northropi*, probably the best known of these species, is more or less only known from the forelimb and two partial femurs and a cervical vertebrae VI in referred specimens (Andres & Langston, 2021), leaving its anatomy to be restored after the much smaller (~4.5 m wingspan) *Q. lawsoni*.

This has led wingspan estimates for these taxa to be variable. In one of the earliest studies on azdarchid wingspan, Lawson (1975) suggested the wingspan of *Q. northropi* could have been anywhere from 11 m to as high as 21 m in width, though they preferred a value of 15.5 m. Subsequent studies tended to revise this downward to the point that estimates of 11–13 m were more common (Andres & Langston, 2021: p. 47). Frey and Martill (1996) estimated the wingspan of *Arambourgiana philadelphiae* at 11.5 m by scaling the estimated original length of the holotype cervical vertebra against *Q. northropi*. However, this was criticized by Witton and Habib (2010: p. 3), who noted this was calculated assuming isometric scaling of the cervical vertebrae in pterosaurs when previous studies had noted this region generally shows positive allometry in Pterosauria (Wellnhofer, 1970). The most recent studies have concluded the largest azdarchids likely had wingspans of 10–10.5 m with wingspans of 11+ m being overestimates (Andres & Langston, 2021; Witton & Habib, 2010), and larger sizes may not have been possible on biomechanical grounds (Witton, 2013: p. 251)

A similar controversy has arisen over the weight of these pterosaurs. Weight estimates for *Q. northropi* have ranged anywhere from 30 kg to 544 kg, depending on the methods used. Henderson (2010) estimated the body mass of *Quetzalcoatlus northropi* at 544 kg using volumetric modeling. However, Witton and Habib (2010) took issue with these results, noting the model used by Henderson (2010) did not reflect the actual proportions of *Q. northropi*; it had an overly large torso that would have massively inflated volumetric-based size estimates. Based on this, they considered more traditional mass estimates of *Q. northropi* at 180-250 kg to be more reasonable and based on this (and other evidence such as larger flight muscles than considered by Henderson, 2010) considered *Q. northropi* and other giant pterosaurs capable of powered flight.

Other issues have arisen regarding pterosaur body mass in the opposite direction, with most studies prior to ~2008 proposing extremely light weights for these animals. *Pteranodon* was proposed to weigh ~20 kg (9.1–30 kg), whereas *Quetzalcoatlus* was often suggested to weigh 70–80 kg (Witton, 2008). These low masses appear to be due to speculative guesstimates, extrapolation error by scaling from much smaller birds and bats, or in some cases assuming that pterosaurs followed a bipedal launch model and thus could be no larger than ~70 kg (Chatterjee & Templin, 2004; Sato et al., 2009; Witton, 2008; Witton & Habib, 2010) However, these proposals would require large pterosaurs to be 80–90% air by volume, leading some researchers to critically refer to this hypothesis as pterosaurs being “hyperlight airbeings” (Paul, 1991). More recent mass estimates have suggested this is not the case, with estimated weights for *Pteranodon* of ~36 kg (Witton, 2008) and 180–250 kg (Witton & Habib, 2010). In reviewing prior mass estimates, Witton (2008: p. 152) writes:

“[t]he idea of pterosaurs being extremely lightweight appears to have grown from observations of their extensively pneumatised skeletons and anecdotal mass figures cited by early workers with no methodological details. This, combined with a consensus that pterosaurs were little more than delicate, weather-dependant gliders apparently entrenched the idea that pterosaurs were atypically lightweight. The evidence that pneumatised bird skeletons occupy just as much mass as those of un-pneumatised mammalian skeletons implies that pneumaticity does not necessarily reduce overall mass and that these early observations regarding pterosaur mass were in error.”

Finally, in 2005 at the 2005 British Association Festival of Science there was a preliminary announcement of the discovery of pterosaurs with an 18–20 m wingspan, based on a wing bone from Jordan and large footprints in Mexico (Radford, 2005). Further examination found the footprints were produced by theropod dinosaurs and the proposed Jordanian wing bone was actually a piece of petrified wood (Witton, 2010: p. 321)

1. Hébert (1855) originally estimated the body mass of *Gastornis* at 500 kg, based on scaling from an albatross. However, in the same year Prévost (1855) suggested a mass of 200 kg by scaling *Gastornis* from a swan. Later, more intensive studies have generally favored the latter estimate, including those based on tibiotarsal or femoral circumferences (108–229 kg; Andors, 1992), egg-to-parent regression models (135–156 kg; Angst et al., 2014, using the dataset of Dyke and Kaiser, 2010), and volumetric estimates (199 kg; Murray & Vickers-Rich, 2004). Note that these measurements involve two species of *Gastornis*, the volumetric and some long bone estimates come from the North American *G. giganteus* rather than the European *G. parisiensis*, the former of which is slightly larger (Angst & Buffetaut, 2017). Additionally, it should also be noted the mass of 135–156 kg from Angst et al. (2014) was calculated assuming *Gastornis* was a precocial bird. If *Gastornis* were altricial, this model produces weights of 66–79 kg (Angst et al., 2014). However, Angst et al. (2014), considered this unlikely as it would require *Gastornis* to have exhibited significant sexual dimorphism and all known *Gastornis* fossils to have come from male individuals, with smaller female individuals being unsampled. Deeming (2022) considered Dyke and Kaiser (2010)’s regression models to be unreliable for prediction, noting they showed significant phylogenetic signal and predicted an egg mass for *Gastornis* less than half the value estimated from fossil egg shells. However, he did not attempt to control for precociality/altriciality in his tests as Dyke and Kaiser (2010) and Angst et al. (2014) did. These results, as well as the scaling differences between Hébert (1855) and Prévost (1855), demonstrate how model choice can dramatically impact the size of extinct animals.

The first recovered tarsometatarsi of *Gastornis* were missing their middle portion, leading early studies to reconstruct *Gastornis* with a very long tarsometatarsus based on analogy with other large, flightless birds such as ratites and phorusrhacids (Buffetaut & Angst, 2013). However, complete *Gastornis* tarsometatarsi show these animals had a very short, stocky foot with blunt, hoof-like claws (Buffetaut & Angst, 2013). This had a relatively minor effect on size estimates (decrease in height of ~13 cm; Buffetaut & Angst, 2013: fig. 4), though it did affect the reconstructed form of this taxa from a relatively long-legged bird to a more stocky one.

Ultimately, the controversies in the body size/form of *Gastornis* are minor compared to those seen in other groups. That said, other spurious features in reconstructions such as a hooked beak, clawed feet, and long tarsometatarsi (all of which are actually absent in *Gastornis*) have led to this taxon being misidentified as a fleet-footed carnivore rather than a stocky herbivore for the better part of a century (Buffetaut & Angst, 2013).

1. Early attempts at mounting moa (Dinornithiformes) skeletons during the late 18^th^ and 19^th^ centuries often exaggerated the height of these animals by mounting their torso in what is now recognized to be an overly erect stance as well as adding additional vertebrae to the mount, especially to the lower cervical series (Worthy & Holdaway, 2002). This appears to be due to a combination of early researchers being unfamiliarity with palaeognath anatomy as well as “a thirst for the tallest mount — but perhaps scant regard for reality” (Worthy & Holdaway, 2002: p. 158).

1. Murray and Vickers-Rich (2004) estimated the body mass of several species of large birds like the dromornid *Dromornis* using volumetric methods as well as the femoral circumference equations of Anderson et al. (1985) and Campbell and Marcus (1992). However, Nguyen et al. (2010) noted that Murray and Vickers-Rich (2004) incorrectly converted the regression equation of Campbell and Marcus (1992) when changing it into a non-logarithmic form, changing it from…

$$\log_{10} \left( \text{body mass} \right)=2.411*\log_{10} \left( \text{femur circumference} \right)-0.065$$

to

$$\text{body mass}=-0.65*\text{femur circumference}^{2.411}$$

instead of

$$\text{body mass}={10}^{-0.065}*\text{femur circumference}^{2.411}$$

This results in a non-functional equation (all resulting body mass estimates are negative). Nguyen et al. (2010: p. 54) note “[i]t is uncertain how Murray and Vickers-Rich (2004) obtained their estimates from this equation, but their [reported] values overestimate the body mass of dromornithids, especially for the heavier birds”. This can be seen by comparing Murray and Vickers-Rich (2004: tab. 11)’s estimates for a sample of 17 *Dromornis stirtoni* using their incorrectly formatted equation against those produced by later authors using the correct equation. Murray and Vickers-Rich (2004) produce an average body mass of 627 kg and a range of 397–829 kg, whereas later studies produce estimates of 519.5 (341.9–713.9) kg (Nguyen et al., 2010: tab. 4), 545 (342–714) kg (Angst & Buffetaut, 2017: tab. 4.1) and 503.7 (316.6-727.8) kg (Handley et al., 2016) using the same size proxy, representing an overestimate of almost 100 kg and around 15–17% of the original estimated body mass.

1. The Cenozoic “pseudo-toothed” birds in the family Pelagornithidae are probably the largest flying birds known (Ksepka, 2014; Mayr & Rubilar-Rogers, 2010). Howard (1957) estimated the wingspan of *Pelagornis orri* at 4.23-4.88 m (14–16 feet). Olson (1985: p. 200) wrote “if anything I would gauge this as an underestimate, with 18 to 20 feet (5.5 to 6 m) being quite likely for the material I have examined from the Miocene of Oregon”. This larger estimate subsequently became embedded in the grey literature (Naish, 2012: p. 402), despite Olson (1985)’s methods of producing this estimate never being detailed. In the 2010s two very large species of pelagornithids, much larger than *P. orri*, were described based on well-preserved material: *Pelagornis chilensis* (Mayr & Rubilar-Rogers, 2010) and *P.* *sandersi* (Ksepka, 2014). These taxa were estimated as having wingspans of 5.25–6.10 m and 6.06–7.38 m, respectively; the methodology for calculating these estimates are well-detailed and worth reading.

However, at the same time, while Olson (1985)’s suggestion of a pelagornithid with a 5.5–6 m wingspan was later validated, this was notably not due to later analyses supporting initial guesstimates but new, much larger pelagornithid material being discovered (Mayr & Rubilar-Rogers, 2010). Both *Pelagornis chilensis* and *P. sandersi* are much larger than *P. orri*, and Mayr and Rubilar-Rogers (2010: p. 1327) suggest Olsen (1985)’s estimate of a 5.5–6 m wingspan for *P. orri* is “exaggerated”. Indeed, if isometrically scaling from overlapping elements and assuming Olson (1985) estimate for *P. orri* was correct, *P. chiliensis* would have a wingspan of 6.1–8.3 m and *P. sandersi* 7.8–9.5 m, far larger than their describing studies find reasonable.

1. The late Cenozoic teratornithids (teratorns) are some of the largest birds to have ever flown, along with the pelagornithids. Wingspans of 6–8 m have been commonly cited for *Argentavis magnificens*, the largest known teratorn species. However, these values appear to be overestimates. Part of the problem is that the holotype and most complete specimen of *A. magnificens* (MLP 65-VIII-29-49) preserves a number of associated elements but the only preserved remains of the wing are a mostly complete humerus and a few bone fragments of more distal elements (Campbell & Tonni, 1983; Chatterjee et al., 2007; Vizcaíno & Fariña, 1999). Other specimens do not preserve forelimb elements aside from a scapulocoracoid fragment (Vizcaíno & Fariña, 1999).

Campbell and Tonni (1980: p. 66) originally estimated *Argentavis magnificens* to have a wingspan of 7.0–7.6 m and a weight of 120 kg by isometrically scaling up from previously reported estimates of *Teratornis merriami*. Later, these authors attempted to more rigorously estimate the size of *A. magnificens* (Campbell & Tonni, 1983). To estimate wingspan, they attempted to circumvent the limited forearm material by estimating the body mass of *Argentavis magnificens* using tibiotarsal circumference, then back-calculate by determining what wing surface area would be necessary to get the resulting animal airborne. At an estimated weight of 78.8 kg, this produced wingspan estimates of 5.7–6.4 m, with one outlier value of 8.3 m (Campbell & Tonni, 1983: tab. 1). Campbell and Tonni (1983) did not attempt to estimate wingspan from humeral length in *A. magificens* despite doing so for the teratorns *T. merriami* despite noting the humerus seems to scale isometrically across teratorns and New World vultures, citing the fragmentary nature of the only known humerus. In the interest of fairness, attempting to estimate the wingspan of *A. magnificens* using the methods discussed and the figured length of the humerus (estimated length ~ 59 cm; Campbell and Tonni, 1983: fig. 2a) would produce wingspans of 5.9–6.1 m. Campbell and Marcus (1992) revise this down slightly to 71.9 kg (wingspan was not recalculated), though they warn about the possibility of extrapolation error in any attempt to estimate the size of these and other large extinct volant birds due to the limited number of similar-sized living taxa.

These estimates of size and wingspan have provoked a great deal of controversy, as they are apparently just on the edge of biomechanical limits for soaring flight and it remains unclear how *Argentavis magnificens* functioned as an organism (Campbell & Tonni, 1983; Chatterjee et al., 2007; Vizcaíno & Fariña, 1999). That is, according to some of these models *A. magnificens* was unable to take off or fly without assistance from the wind (Campbell & Tonni, 1983; Chatterjee et al., 2007; Vizcaíno & Fariña, 1999). However, it is quite likely that *A. magnificens* was not as large as Campbell and Tonni (1983) predicted. Mayr and Rubilar-Rogers (2010) estimated *A. magnificens* had a wing skeleton span of roughly 183 cm. In order for prior wingspan estimates of *A. magnificens* to be correct, this would require primary feathers nearly 1.5 m long (Chatterjee et al., 2007), which would be exceptionally large in proportion among birds and contradict the observation that primary feathers usually show strong negative allometry with increasing size (Ksepka, 2014). Ksepka (2014) predict total wingspans of 5.09–6.09 m for *A. magnificens* using a number of different starting assumptions.

Witton (2019) noted previously cited weight estimates of 70–80 kg would be exceptionally heavy for a bird with a wingspan of < 6 m. He suggested weights of 25–40 kg for *A. magnificens* might be more likely based on his own wingspan-body mass calculations in a sample of 90 birds, but did not provide the data or calculations necessary to independently verify these results. Witton (2019) further suggested that the much higher mass estimates calculated via tibiotarsal circumference might be due to the more stork-like, terrestrial habits proposed for teratorns (Campbell & Tonni, 1983) rather than being exclusively soaring birds like vultures. These wingspan and weight values would clearly take *A. magnificens* out of the running for largest bird (Witton, 2018). Nevertheless, wingspans as large as 8 m for *A. magnificens* continue to be cited in the gray literature (Chatterjee, 2015: p. 179).

1. Early literature on large extinct penguins like *Anthropornis* and *Pachydyptes* often suggested standing heights of 1.5–2 m (see review in Ksepka et al., 2012). One of the most extreme estimates comes from Hector (1872), who estimated the height of *Pachydyptes ponderosus* at “six to seven feet” (1.8–2.1 m); this estimate was subsequently repeated in the gray literature for nearly 100 years (Simpson, 1976) despite being considered a “gross overestimate” by Ksepka et al. (2012: p. 252). Other appear to be overestimates due to an under-appreciation of non-isometric scaling in skeletal elements across penguins, as well as stem-penguins having proportionally longer forelimbs than crown penguins. Ksepka et al. (2012) are skeptical of any estimates of standing height in extinct penguins over 1.5 m.
2. Controversies in the estimated size/form of extinct animals are not new. Newman (1970) attempted to reconstruct the posture and locomotor behavior of the famous tyrannosaurid *Tyrannosaurus rex*. However, he had trouble getting his model to work based on the famous reconstruction at the American Museum of Natural History (AMNH 5027). Newman (1970) noticed the tail of *Tyrannosaurus* was unusually long compared to the complete caudal series known for *Gorgosaurus*. and subsequently discovered that only the anterior 20 caudal vertebrae of the mount were real, the rest were plaster reconstructions. Reconstructing the caudal vertebrae after removed nearly 12 feet (3.6 m) of length from the resulting reconstruction, reducing the length of *T. rex* from ~15 m to ~12 m (Newman, 1970). This resulted in the skeleton of *Tyrannosaurus* being able to balance in a horizontal posture with the tail off of the groun, and was one of several discoveries that led to more active depictions of dinosaurs during the “Dinosaur Renaissance” of the 1970s–1980s. The body form of *Tyrannosaurus* has become much better known since the 1970s due to the discovery of several spectacularly preserved skeletons (e.g., Brochu, 2003; Persons et al., 2020).
3. The size and body form of *Spinosaurus* has been controversial. Dal Sasso et al. (2005), based on a large, isolated premaxilla of *Spinosaurus* (MSNM V4047), suggested a length of 16–18 m based on isometric scaling from *Baryonyx* and *Suchomimus* and 7–9 metric tons using the methods of Seebacher (2001), which would make it significantly larger than other “mega-theropods” such as *Tyrannosaurus* and *Giganotosaurus*. However, Therrien and Henderson (2007) criticized these estimates, noting that skull size scales with positive allometry in theropods, resulting in a length of 14 m or less (potentially as short as 12.57 m). They also note that Dal Sasso et al. (2005)’s weight estimate is unusually low, noting that scaling up Seebacher (2001)’s estimate for *Suchomimus* to a 16–18 m animal produces weights of 11.7-16.7 metric tons, and their own methods produce a weight of 20.9 metric tons for a 14 m animal.

Ibrahim et al. (2014) presented a revised reconstruction of *Spinosaurus*, largely based on a single, associated specimen (FSAC-KK 11888) but with a significant amount of scaling of fragmentary material to fill in the gaps. This reconstruction is noted to have very short legs, which has led some to call it a chimera (Hartman, 2014); however, this has mostly died down with additional evidence being presented that FSAC-KK 11888 represents a single individual (Ibrahim et al., 2020). The proportions of *Spinosaurus* are of particular concern because they have led to a debate over the location of the center of gravity of the animal and therefore whether it was quadrupedal or bipedal. This, however, has not exempt the Ibrahim et al. (2014) model from criticism; Sereno et al. (2022) suggested Ibrahim et al. (2014) overestimated presacral length by 10%, ribcage depth by 25%, and forelimb length by 30%, which drew the center of mass forward and resulted in an anomalous reconstruction of the center of mass.

1. Grillo and Delcourt (2017) estimated the size (total length) of a number of abelisauroid taxa using a large number of regression equations, including cranial dimensions (skull length, skull height, skull roof dimensions, maxilla length and height), vertebral centra length, width, and height, scapulocoracoid length, pelvic dimensions (iliac length, ilium height, and public boot length), and the lengths and widths of appendicular elements. These equations were constructed based on the few abelisauroid taxa for which relatively complete remains were known (*Carnotaurus*, *Aucasaurus*, *Masiakasaurus*, *Majungasaurus*, *Eoabelisaurus*, etc.). After producing these models, Grillo and Delcourt (2017) examined the strength of their correlation as well as if any models produced outlier values due to autapomorphic or incorrectly reconstructed features of many taxa, eventually producing a mean value of the resulting non-spurious estimates.

For most abelisauroid taxa total length was within +/- 10% of previous length estimates. However, for a number of taxa (mostly larger-bodied abelisaurids) predicted total lengths were much smaller than previously estimated values by 20% or more (Table S6). In one case, *Pycnonemosaurus nevesi*, the estimated total length was actually larger than previously proposed (8.93 m versus 7–8 m) estimated leading these authors to regard *Pycnonemosaurus* as the largest abelisaur. This conclusion seemed to be supported by overall size of the elements of the taxa in question (Grillo & Delcourt, 2017: fig. 1). Once these cases of potentially anomalous body size were resolved, the authors noticed a pattern of increasing body size in Abelisauridae across the Cretaceous. In reviewing the results of their study, Grillo and Delcourt (2017: p. 83) write “the fact that most published BL [= total lengths] are overestimates reinforces a statement made by Therrien and Henderson (2007) that the lack of complete skeletal remains in large theropods gives free course to imagination, that allow researchers to present new specimens as ‘the largest’, ‘the heaviest’, or other kind of similar adjectives”.

At least some of these differences in length estimates appear to be driven by differences in proportion and thus body form across abelisauroid lineages. For example, *Ekrixinatosaurus novasi* was estimated at 7–8 m based on hind limb elements (Calvo et al., 2004) but 10–11 m based on cranial elements (Juárez Valieri et al., 2010), both calculated by simple scaling from the carnotaurins *Carnotaurus* and *Aucasaurus*. However, Novas et al. (2013: p. 176) noted the limb bones of *Ekrixinatosaurus* were about 15% longer than those of *Carnotaurus* and Juárez Valieri et al. (2010: p. 162) noted the skull to femur ratio of *Carnotaurus* is unusually low. Juárez Valieri et al. (2010) justified their choice of proxy by suggesting *Ekrixinatosaurus* showed a *Carnotaurus*-like skull but a short femur, but Grillo and Delcourt (2017) noted that cranial dimensions were very poor predictors of body size in abelisauroids and the postcranial morphology of *Ekrixinatosaurus* is better explained by allometry. Overall, this shows how differences in observed or interpreted body form can feed back into estimates of body size. Grillo and Delcourt (2017) also suggest some of these issues are due to most of these studies using simple scaling ratios (“rule of three”) rather than allometric regression equations, making them prone to allometric bias.

One caveat of Grillo and Delcourt (2017)’s is that the distal caudal vertebrae are unknown for most abelisaurs, so the authors standardized the measurements by assuming total length was 2.08 times precaudal length based on the caudal skeleton of *Majungasaurus crenatissimus* (O'Connor, 2007). Interspecific differences in tail length are expected to influence in total length (Hone, 2012), but likely not by enough to account for the differences in lengths between studies. Indeed, by standardizing tail length Grillo and Delcourt (2017) are effectively comparing abelisauroids by snout-vent length, which is considered a more reliable correlate of size in most reptiles (Hone, 2012).

1. *Dreadnoughtus schrani*, a (relatively) complete giant late Cretaceous titanosaurian sauropod, was originally estimated to weigh 59300 kg (Lacovara et al., 2014) based on the combined humeral and femoral circumference to body mass regression of Campione and Evans (2012). However, Bates et al. (2015) found this mass to be unreasonably high when trying to replicate it with volumetric methods, finding a maximum possible body mass of 38000 kg. This value was significantly below the lower bound of the percent prediction error using the equation of Campione and Evans (2012) (44095 kg), though not outside the lower bound of the 95% prediction interval. A weight of 59300 kg would require hyper-dense tissues beyond what is seen in living vertebrates (Bates et al., 2015). Bates et al. (2015) notes similar overestimates of body mass when these methods were applied to the sauropods *Apatosaurus louisae* and *Giraffatitan brancai*.

Based on these suggestions Campione (2017) re-evaluated the humerus + femoral circumference body mass equation of Campione and Evans (2012) with regards to *Dreadnoughtus*, specifically testing for the possibility of non-linear allometry (log-quadratic versus log-linear scaling). Campione (2017) found that support statistics (%PE, %SEE, AICc) for the log-quadratic and log-linear model were about the same, but the log-quadratic model produced masses for sauropods that were much closer to those estimated by Bates et al. (2015). However, they also noted that the curvature in a quadratic relationship is highly subject to the shape of the underlying predictor set (especially at extreme values), leading to unpredictable outcomes and potentially higher error. This potentially raises the question of whether it is better to produce a stable (precise) but wrong (inaccurate) estimate using a log-linear model versus a potentially accurate but unstable (imprecise) result via a log-quadratic one (compare with the broader statistical discussion of accuracy versus precision; National Academies of Sciences, 2019: 47-51)

1. When initially described, “*Seismosaurus” hallorum* (now *Diplodocus hallorum*) was originally estimated to have measured 39–52 m in length and weigh more than 100 tons based on simple isometric scaling from specimens of *Diplodocus* (Gillette, 1991, 1994), with the author “the best approximation would fall towards the higher end of this range (i.e., approaching 50 meters)” (Gillette, 1991: p. 431). This was largely based on three criteria: 1) assuming the great height of the neural spines reflects an allometric increase in the lengths of the neck and tail, 2) the articulated vertebrae Gillette (1991) identified as caudals 20-27 appeared to have disproportionately tall neural spines and be disproportionately robust, which they again took as indications of a dramatically elongate tail, and 3) the relatively high anteroposterior lengths of the pubis and chevrons were taken as indicating a dramatically elongated tail (Gillette, 1991; Herne & Lucas, 2006). As noted by Herne and Lucas (2006), criteria 1 and 3 were not reliable anatomical proxies for scaling total length. As for criterion 2, the articulated caudals Gillette (1991, 1994) identified as caudals 20–27 were actually probably 12–19 (Curtice, 1996; Herne & Lucas, 2006), which better agrees with the development of their neural spines and massively reduces lengths for the animal. Later studies have proposed length estimates of 30.5–35 m, with the holotype specimen as mounted (with the caveat that some elements are reconstructed) having a total length of 33 m (Curtice, 1996; Herne & Lucas, 2006; Paul, 1997). Paul (1997: p. 136) also notes “Gillette’s 100 ton estimate exceeds the volume of his own skeletal restoration three to four fold”.
2. *Futalognkosaurus dukei* is the most complete of the Patagonian "mega-sauropods", with the holotype specimen being over 70% complete and preserving a near-complete pre-sacral axial skeleton. *Futalognkosaurus* was originally reported to be comparable in size to *Argentinosaurus* and *Puertasaurus*, with an estimated length of 32–34 meters (Calvo, 2006; Calvo et al., 2007). However, as the extent of the holotype became better understood, this length was revised downward to 26 m (Calvo et al., 2008). Calvo et al. (2008) suggest similar adjustments may need to be applied to other Patagonian mega-sauropods like *Argentinosaurus* and *Puertasaurus*.
3. *Bruhathkayosaurus* is an extremely complicated situation. This taxon was originally described based on material from the late Cretaceous of India, originally described as a theropod (Yadagiri & Ayyasami, 1987) but later referred to a titanosaur based on size. Depending on how the material is scaled, this either results in an animal around the size of the otherwise largest sauropods from the late Cretaceous of Argentina (e.g., *Argentinosaurus*) (Molina-Pérez & Larramendi, 2020) or an utterly gargantuan creature (~45 m) significantly larger than any sauropod previously described (Benton, 2023; Mortimer, 2004; Paul & Larramendi, 2023). Unfortunately, all known remains of *Bruhathkayosaurus* are now lost. Bones from the Kallamedu Formation are waterlogged and extremely friable, and monsoonal variations in humidity between day and night meant that the bones actually crumbled to dust between being excavated in the field and reaching a museum (Galton & Ayyasami, 2017; Pal & Ayyasami, 2022). Thus, the only remaining evidence of *Bruhathkayosaurus* are photographs of the tibia(?) and pubis taken in situ in the field and the measurements reported in Yadagiri and Ayyasami (1987).

This, naturally, has caused a great deal of controversy regarding the identity of *Bruhathkayosaurus*, especially given the reported extreme size of the material cannot be independently verified. The photos of the *Bruhathkayosaurus* holotype lack any true scale, the only indication of size being a rock hammer included in the photos reported to be about 40 cm long (Pal & Ayyasami, 2022). It also did not help that the photos as originally published in Yadagiri and Ayyasami (1987) were low-quality and obscured much of the detail of these specimens. This led some authors to even suggest the remains of *Bruhathkayosaurus* may be misidentified pieces of petrified wood (Brusatte, 2001; Holtz, 1995; Pal & Ayyasami, 2022; comments in Wedel, 2008). These criticisms mostly died down after Pal and Ayyasami (2022) republished the photos in higher quality, confirming the titanosaurian identification. There have also been debates over what bones the limb elements of *Bruhathkayosaurus* represent; that is, are they a femur, tibia or fibula. Which elements the limb bones are referred to drastically affects the resulting size estimates (Molina-Pérez & Larramendi, 2020; Mortimer, 2004; Paul, 2019). Mass estimates for *Bruhathkayosaurus* range anywhere from 30 to 220 tons (Molina-Pérez & Larramendi, 2020; Mortimer, 2004; Paul & Larramendi, 2023; Wedel, 2008). It is unlikely the question of this taxon’s size will be resolved until more material is discovered.

1. *Maarapunisaurus fragillimus* was originally described as *Amphicoelias fragillimus* by Cope (1878) based on a gigantic partial vertebra missing the centrum from the Late Jurassic Morrison Formation of Colorado. The neural spine of this specimen was reported as being 1.8 m in length. Unfortunately, this specimen is now lost, and was possibly destroyed during transport due to its very fragile nature (Carpenter, 2018). "*A*." *fragillimus* was initially identified as a diplodocid (Osborn & Mook, 1921), an opinion followed by most studies thereafter, which if true would result in an absolutely massive animal potentially up to 60 m long (though possibly as low as 40 m long) based on scaling the height of the neural spine from other diplodocids (Carpenter, 2006, 2018; Paul, 1994). Carpenter (2018) reviewed the available based on Cope (1878)'s drawings, and concluded it was likely a rebbachisaurid, referring it to the new genus *Maarapunisaurus*. Because rebacchisaurids have proportionally much taller neural spines than diplodocids, this would result in an animal 30.3–32 m long rather than 60 m.

1. Brassey et al. (2015) attempted to estimate the body mass of a near-complete skeleton of *Stegosaurus stenops* (NHMUK R36730) using both the humerus/femur circumference equations of Campione and Evans (2012) as well as volumetric mass estimation. Three distinct convex hull models assuming different amounts of soft tissue were used; the preferred model returned a value of 1560 kg (95% P.I. = 1082–2256 kg) whereas the stockiest model produced an estimate of 1894 kg (95% P.I. = 1303–2760 kg). By contrast, humerus/femur circumferences produced an estimated mass of 3752 kg (95% P.I. = 2790–4713 kg) using a bivariate model and 3329 kg (95% P.I. = 2499–4159 kg) using a multivariate one. There is almost no overlap between the two models, even if considering the highest bound of the heaviest volumetric model and the lowest bound of the humerus/femur models. Brassey et al. (2015) suggested this may be due to the “immature” state of the specimen (the neurocentral sutures are closed but still partially visible and histologically the specimen does not have an external fundamental system, suggesting a young adult), though other specimens have noted similar discrepancies (Bates et al., 2015; Romano & Manucci, 2021).

1. Sulej and Niedźwiedzki (2019) described the very large dicynodont *Lisowicia* from the latest Triassic of Poland. They estimated its mass at ~9000 kg (9330 kg) based on combined humeral and femoral circumference (from Campione & Evans, 2012) and described this taxon as being “elephant-sized”. However, Romano and Manucci (2021) found this mass estimate to be much too high, based on their volumetric methods, which produced an average body mass of 5880 kg (range 4870–7020 kg). This would make *Lisowicia* only around the size of a rhinoceros. Romano and Manucci (2021) suggesting the high mass estimates reported by Sulej and Niedźwiedzki (2019) for *Lisowicia* were due to not taking into account the very robust limb bones of this taxon into account.
2. *Thylacinus* is an odd case as this animal is only recently extinct and therefore a few live weights of this taxon were collected while it was still extant. Nevertheless, we consider it here due to it no longer being possible to directly verify estimated weights through measurements of live individuals. *Thylacinus cynocephalus* has often been cited as weighing approximately 29.5 kg (Paddle, 2000), which would imply it was anomalously large relative to the size of prey this animal is thought to have eaten (i.e., exceeding the threshhold between carnivorous mammals eating large and small prey identified by Carbone et al. 1999; see Rovinsky et al., 2020; Wroe et al., 2007).

The commonly-cited 29.5 kg estimate for *T. cynocephalus* appears to be derived from an “uncritical reading of anecdotal source material” (Rovinsky et al., 2020), i.e., a back-of-the-envelope field estimate. Only four reliable in vivo mass estimates are available for *T. cynocephalus*; three ranging from 13.2–15 kg, and one anomalously large estimate of 26.1 kg, mostly from captive individuals. Estimating the mass of a number of individuals using volumetric and other methods produces a mean estimated body mass of 13.7 kg for female thylacines and 19.7 kg for males, though with some exceptional individuals potentially reaching 28 kg, much smaller than prior estimates that treated 29.5 kg as an “average” size. (Rovinsky et al., 2020)

The use of the aforementioned 29.5 kg estimate as “typical” for *Thylacinus cynocephalus* is actually a significant issue, as previous studies used this value for *T. cynocephalus* when creating regression equations for carnivorous marsupials (Myers, 2001). Given *T. cynocephalus* is by far the largest recent dasyuromorphian and one of only a few larger than 1 kg, this results in *T. cynocephalus* having an outsized influence on the slope of allometric regression equations for the group (Rovinsky et al., 2020). Combined with the 29.5 kg overestimate, this potentially means body mass estimates created from these equations for extinct thylacinids like *Thylacinus potens* (as well as other large carnivorous metatherians to which they have been applied like sparassodonts) may be biased and slight overestimates (Rovinsky et al., 2020). As an example, the body mass of *T. potens* may have been overestimated by nearly twice its actual value due to this (Rovinsky et al., 2020).

1. Early size estimates for *Thylacoleo* *carnifex*, the marsupial lion of Pleistocene Australia, were very shaky. Early authors described this animal as “wolf-sized” or “leopard-sized” and offered weights ranging from 40–100 kg, with most towards the lower end of that spectrum (Wroe et al., 1999). Webb (1998) went so far as to suggest *T. carnifex* was only 20 kg in weight! Wroe et al. (1999) points out that none of these estimates were based on quantitative methodology, merely subjective assessments or gross comparisons with living taxa.

Wroe et al. (1999) estimated the weight of *Thylacoleo carnifex* using humeral circumference, femoral circumference, and combined humeral and femoral circumference, producing body mass estimates ranging from 73–164 kg (aside from one anomalously small individual) and an average estimated body mass of 101–130 kg. This would make *Thylacoleo* roughly the size of a female lion (Haas et al., 2005; Wroe et al., 1999). As an alternate test, Wroe et al. (2003) estimated the body mass of *T. carnifex* using endocranial volume-body mass regression equations within its own clade (Diprotodontia), and produced rather similar mass estimates to those created through postcranial dimensions. It is rather noteworthy that in this survey of controversial size estimates, *Thylacoleo* is one of the only cases where revision have resulted in robust size estimates suggesting a larger animal.

1. As is the case with many Australian megafaunal marsupials (see point 61 above) early estimates for *Diprotodon* were rather vague, making a priori comparisons with bulls (Long et al., 2003) and rhinoceroses (Archer et al., 1994) (see discussion in Wroe et al., 2004). Wroe et al. (2004) estimated the body mass of a near complete ~3.7 m long (estimated 4 m with soft tissue) individual of *Diprotodon optatum* mounted at The Australian Museum (specimen number not provided) at 2786 kg based on combined humeral and femoral circumference. This is significantly larger than the average individual of a hippopotamus (*Hippopotamus amphibius*) or white rhino (*Ceratotherium simum*), though not as high as the maximum known weights for these species. As with *Thylacoleo*, *Diprotodon* turned out to be somewhat larger than earlier estimates, though the disparity in size was not as great.
2. *Procoptodon* was originally reported as reaching heights of nearly 3 m (10 feet) tall (Flannery, 2008). However, this appears to be an overestimate due to several criteria including overestimation of the original skeletal material, conflating browsing envelope (i.e., with arms extended overhead) with actual height, and not recognizing that sthenurine kangaroos were incapable of the erect posture seen in extant macropodines (Musser, 2018), with *Procoptodon* probably being around 2 m tall, about the same size as a large male red kangaroo (but more massive at ~250 kg; Helgen et al., 2006). Even in the recent literature, competing height estimates of either 2 m (Archer et al., 2023; MacPhee, 2018) or 3 m (McNamara & Murray, 2010: p. 85; Prothero, 2016: p. 45) are commonly cited.

1. Larramendi (2015) discussed a gigantic distal femur of the proboscidean *Palaeoloxodon namadicus* reported by Prinsep (1834) from Sagauni in India, said to be approximately 20% larger than a 160 cm long femur from another individual. If true, this would result in a femur nearly 190 cm in length, resulting in an elephant with a shoulder height of 520 cm and an estimated weight of 22 metric tons. Based on this, Larramendi (2015) suggested that *P. namadicus* was the largest known land mammal, larger than even the biggest paraceratheriid rhinoceroses. However, Larramendi (2015: p. 559) notes that they did not examine the specimen firsthand and current location of the specimen is uncertain, though they suggest it may be somewhere in the Indian Museum of Kolkatta. Because of this, they suggest such a size estimate should be treated as speculative.
2. *Paraceratherium* (also known as *Indricotherium* and *Baluchitherium*) is a giant rhinoceros (paraceratheriid rhinocerotoid) best known from the early Oligocene of Asia (Pakistan, Mongolia, Kazakhstan). This taxon is generally considered to be the largest land mammal that has ever existed (but see Larramendi, 2015). Complete skeletons are unknown, requiring reconstructing the size and proportions of this animal after composites of fragmentary individuals. Estimates of 20–34 tons were once frequently cited for this species, which appear to be largest based on the famous reconstruction by Helen Zizka in Gregory and Granger and Gregory (1935, 1936). However, as Fortelius and Kappelman (1993: p. 86) note, this reconstruction “was not simply a composite of the fossils at hand, but involved considerable extrapolation based on rather questionable assumptions”. Granger and Gregory (1935, 1936) created their reconstruction by dividing their material into four size classes assuming similar proportions to a modern rhinoceros and scaling smaller material up to the size of the very largest size class, which was only represented by two cervical vertebrae and a partial metacarpal. As Fortelius and Kappelman (1993: p. 86) note: “the famous reconstruction was thus created by simple isometric scaling up of all elements, save two cervical vertebrae and a metacarpal, by more or less arbitrarily assigned constants.” This is especially concerning given *Paraceratherium* is known to have a comparatively elongated neck relative to a modern rhinoceros (Fortelius & Kappelman, 1993).

Granger and Gregory (1935, 1936)’s reconstruction also appears to overstate the head-body length of the animal. This reconstruction results in a head-body length of 870 cm, whereas the reconstruction in Gromova (1959), created based on some of the most complete *Paraceratherium* material known, produces a head-body length of 740 cm (Fortelius & Kappelman, 1993). Gromova (1959)’s reconstruction produces a considerably more gracile animal, which might be expected given that *Paraceratherium* is known to have more elongate limbs and neck than a modern rhinoceros, whereas Granger and Gregory (1935, 1936) scaled up their material assuming rhinocerotid-like proportions. Fortelius and Kappelman (1993), using a wide variety of scaling proxies including skull lengths, head-body length, tooth and dental row lengths, and limb bones dimensions, estimate an average body mass of 9.5–11.1 tons, with the very largest individuals potentially reaching weights of 15–20 tons. The various size proxies largely agree in their estimated masses. Paul (1997) suggests Gromova (1959)’s reconstruction might have been a little too gracile, but his estimates of 7.8 (for a medium-sized individual) and 16.4 tons (for the largest individuals) largely agree with Fortelius and Kappelman (1993) With this, the largest paraceratheriid rhinos seem to have been similar in size to the largest proboscideans (Fortelius & Kappelman, 1993; Larramendi, 2015).

1. To our knowledge, the body size of *Elasmotherium* has not been the subject to controversy, though we would not be surprised if this historically was the case given it is one of the largest known rhinocerotoids outside of *Paraceratherium* (Kosintsev et al., 2019), with Siberian specimens having an approximate body length of 4.5 m and a shoulder height of 2 m and Caucasian specimens potentially reaching 5.0–5.2 m in length and a weight of 4–5 tons (Zhegallo et al., 2005), and thus is in a similar position to many of the other taxa detailed here. However, its body form has undergone a quite striking revision in recent years. *Elasmotherium* has typically been reconstructed with a massive, single horn nearly 2 m in length (Prothero & Schoch, 2002). However, while many reconstructions of *Elasmotherium* have shown a large horn, we have been unable to pinpoint where this 2 m estimate was first proposed in the literature; we suspect it may be a “fox terrier problem” (sensu Gould, 1987). The presence of such a huge horn was inferred based on the large frontal boss on the animal’s skull “which implies a horn base much larger than in any other rhinoceros, living or extinct” (Kosintsev et al., 2019: p. 31; see also Zhegallo et al., 2005). However, re-examination of this cranial boss suggested such a horn was not present; the dome lacked annular rugosities that would indicate a long, projecting horn was present and had rather thin walls suggesting it probably could not support the large horn of traditional depictions (Titov et al., 2021). A similar interpretation had been proposed previously but was not considered popular among researchers (Teryaev, 1948). This situation is very similar to many of the body size examples discussed here, where initial research proposed a relatively sensationalist explanation for unusual fossil morphologies that was subsequently revised to something less extreme.

1. Lambert et al. (2010) estimated the total length of the giant macroraptorial sperm whale (stem physeteroid) *Livyatan melvillei* by scaling from two anatomical proxies: the extant sperm whale *Physeter macrocephalus* and the stem physeteroid *Zygophyseter varolai* (estimated TL 6.5–7 m; Bianucci & Landini, 2006). The former produced an estimated total length of 13.5 m and the latter an estimated total length of 16.2–17.5 m. Many popular articles tended to either report the mean of these two estimates (~15.5 m), or the larger *Zygophyseter*-based ones (~17.5 m). However, paleoartist Tosha Hollman noted that trying to restore the postcrania of *Livyatan* assuming proportions similar to the near-complete holotype of the stem physeteroid *Brygmophyseter shinensis* (SFM 00001; Kimura et al., 2006) produced an estimated total length of 12.9 m (Hollman, 2022). He also noted that attempting to restore the skeleton of *Zygophyseter varolai* from published material produced a shorter animal than initially reported (~ 5 m versus 6.5–7 m), which might suggest the 16–17 m *Zygophyseter*-based estimate for *L. melvillei* may be too high. However, several vertebrae from different sections of the vertebral column are missing from the *Z. varolai* holotype (Bianucci & Landini, 2006). Published photos of the mounted holotype of *B. shinensis* seem to support Hollman’s claim that this taxon resembles *Physeter* in having a large head relative to body size (Kumiko, 2015), although in the former the short cumulative length of the caudals (accounting for distinctly less than 50% of the post-thoracic segment of the vertebral column) is surprising. While Hollman’s proposal received some positive feedback from fossil cetacean experts (B. Boessenecker), a formal paper has not been published and so this should be treated with some reservations. Nevertheless, this suggests that the size and scaling relationships of stem physeteroids may need to be revisited, and that the estimated total length of *L. melvillei* is dependent on which taxon is identified as an anatomical proxy.
2. Savage (1973) produced a body mass of 880 kg for *Megistotherium ostothlastes* based on two methods. First, he estimated the body mass using the endocast assuming a brain-body mass relationship similar to *Hyaenodon*, producing a body mass of 1000 kg. Then, he estimated the mass using distal humeral width compared to a bear, producing a mass of 760 kg. He took the mean of these two estimates, producing a mass of 880 kg.

Both of these methodologies are potentially spurious for different reasons. In the case of brain size, relative brain size is generally not considered a very reliable estimator of body size due to its interspecific variability (Wroe et al., 2003 being an exception). Scaling based on relative brain/body proportions assumes that brain size has remained constant across hyaenodont evolution, something which is not clear especially as hyaenodonts seem to show an expansion of the neocortex over geological time similar to other Cenozoic mammals (Dubied et al., 2019; Flink et al., 2021). Scaling based on relative brain size also assumes a particular body-brain scaling relationship required to calculate encephalization quotient, but exactly how mammalian brains scale with body size has been contentious with several different scaling relationships proposed (Burger et al., 2019; Jerison, 1973; Venditti et al., 2024).

For humeral width, it is important to clarify that Savage (1973) did not produce this estimate via simple isometric scaling from a specimen of known size. Instead, he assumed it might “be reasonable to suggest that a bear with humeral width of 12.3 could weigh around 760 kg” (Savage, 1973: p. 503–504), and then extrapolated from that. This means this estimate is essentially an a priori assumption of an a priori assumption, and thus the estimate based on this method is dubious. The humerus used in this calculation may not even belong to *Megistotherium* and may instead pertain to *Amphicyon* (Ginsburg, 1980).

To the credit of Savage (1973), he did critically evaluate his estimates, considering the endocranial mass estimate to be unrealistically high and the humeral-based mass estimate to be unrealistically low; this is one reason he took the average of the two measurements to produce his 880 kg estimate. Work in progress on these taxa suggests body masses of 300–400 kg are more likely (Engelman, 2020, in prep). Radinsky (1977) offered a similar estimate of 313–423 kg by “model[ing] after other creodonts”, but did not detail his methods. Based on Table 1 in Radinsky (1977), it appears likely he estimated head-body length assuming the head was 4.0–4.5 times skull length, as in other hyaenodonts, and then estimated mass using the head-body length/body mass allometric regression equation provided in the caption.

Because Savage (1973)’s size estimates have often been used as the basis for giant hyaenodonts significantly larger than any living carnivoran, it calls into question size estimates for other giant hyaenodonts such as *Hyainailouros*, a close relative of *Megistotherium* that has sometimes been regarded as congeneric (Morlo et al., 2007; Turner & Antón, 2007). Other methods for estimating body size in hyaenodonts exist (Morlo, 1999), but these are mostly based on dental metrics in smaller species and whether they can be extrapolated to animals as large as *Megistotherium* or *Hyainailouros* remains to be empirically confirmed.

It is also worth mentioning that hyaenodonts in general tend to have disproportionately large heads compared to carnivorans, which results in cranio-dental variables typically overestimating the body size of these taxa in general (Egi, 2001; Savage, 1973; Van Valkenburgh, 1990). This is potentially relevant given size estimates for hyaenodonts are sometimes based on craniodental regression equations in carnivorans in the absence of postcrania (Borths & Stevens, 2019; Flink et al., 2021). Borths and Stevens (2019) even note that dental-based mass estimates were not ideal for the taxon they studied, but was the best size proxies available at the time, with the hope that future studies could refine these estimates.

1. The early Miocene *Megalictis ferox* is one of the largest mustelids to have ever existed. When this species was first described Matthew (1907: p. 196–197) its skull was compared in size to a jaguar (*Panthera onca*) or black bear (*Ursus americanus*) and this comparison has continued in later works (Valenciano et al., 2016). However, although its skull may have been similar in size to a jaguar/black bear, whether the entire animal was similar in size to a jaguar/black bear remains unclear. Nevertheless, comparisons to the whole-body size of a jaguar or black bear remain common in the grey and popular literature (Kingdon, 1988: p. 64; Woodruff & Johnson-Random, 2024: p. 439).

Hunt and Skolnick (1996) estimate the head-body length of *Megalictis* at 90–120 cm and its body mass as 25–65 kg. However, they do not detail how they obtained this mass estimate. It is possible they calculated this via head-body length; inputting a head-body length of 120 cm into the equation of Nelson et al. (2023) produces a weight of 52.7 kg, which is close to what Hunt and Skolnick (1996) obtain. Valenciano Vaquero (2017: tab. 13) estimated the weight of several individuals of *M. ferox* as potentially spanning 30.02–53.99 kg based on postcranial measurements, with some very large but fragmentary individuals possibly reaching ~67 kg Valenciano Vaquero (2017: p. 262). These estimates are significantly smaller than a jaguar or black bear (~70–120 kg; Larivière, 2001; Seymour, 1989), closer in size to a gray wolf (*Canis lupus*) or mountain lion (*Puma concolor*) (Valenciano Vaquero 2018: p. 262). This smaller size makes a bit more sense given remains of *Megalictis* have been found in possible burrows (Hunt, 2011), and extremely large burrowing mammals are rare.

1. A variety of giant chinchilloid rodents (Dinomyidae and Neoepiblemidae) have been known from South America, dating all the way back to *Telicomys gigantissimus* (sometimes considered a junior synonym of *T. giganteus*). This species was often described being “as large as a small rhinoceros” (Patterson & Pascual, 1968; Simpson, 1980), though as noted by (Defler, 2018: p. 153) “these comparisons never specify which species of rhinoceros, which vary in weight from about 500 to 3200 kg among the five species”.

One of the first quantitative investigations of body size in these taxa came with the description of well-preserved postcranial material of *Phoberomys pattersoni* by Sánchez-Villagra et al. (2003). These authors estimated the body mass of *P. pattersoni* using the circumferences of the humerus and femur, which produced body mass estimates of 436 and 731 kg, respectively. Sánchez-Villagra et al. (2003) considered the latter body mass, based on the assumption that *P. pattersoni* engaged in hindlimb-dominated locomotion similar to some living caviomorphs, and this correlated with a more robust femur compared to the humerus. Based on this, popular coverage for the discovery frequently referred to *P. pattersoni* being “a rodent as big as a buffalo” (Alexander, 2003).

Several years later, Rinderknecht and Blanco (2008) reported a near-complete skull of the dinomyid *Josephoartigasia monesi*, which these authors regarded as the largest species of rodent known to have ever existed. They provided a mean estimated body mass of 1211 kg based on scaling from several (much smaller) extant caviomorph rodents, greater than the maximal body mass of male American bison (*Bison bison*; Banfield & Novakowski, 1960; Halloran, 1961; Meagher, 1986) and within the range of variation observed for black rhinoceroses (*Diceros bicornis*; Hillman-Smith & Groves, 1994). However, this value was calculated based on a very small sample size — 13 individuals representing 9 species — and was calculated by uncritically taking the mean of seven mass estimates spanning a very wide range of 468–2584 kg (Millien, 2008; Rinderknecht & Blanco, 2008).

Many criticisms were made of the mass estimates produced in these studies. Almost immediately after the publication of Rinderknecht and Blanco (2008), Millien (2008) criticized several aspects of this study including their extremely small sample size, the morphological size proxies chosen, their failure to log-transform the data before estimating body size, and their failure to account for potential extrapolation error.

Both Hopkins (2008), for *Phoberomys*, and Millien (2008), for *Josephoartigasia*, noted that the cheek tooth series of these taxa (P4–m3 or p4–m3) relative to published mass estimates were unusually small when placed in a broader allometric analysis of rodent tooth proportions, suggesting that one or more of the anatomical proxies used to estimate the body mass of these taxa “violates the fundamental requirement of a body mass proxy that ‘the fossil taxon for which a value is predicted must be a member of the population from which the taxa used to generate the prediction equation are a sample’ (Smith 2002: 276)” (Hopkins, 2008: p. 239). Or, to use the terminology of the present paper, one or more of the anatomical proxies used to estimate the size of *Phoberomys* and *Josephoartigasia* did not scale in a way to make it appropriate to apply to these species. Hopkins (2008: p. 239) also criticized the a priori decision to treat the largest estimate of *P. pattersoni* as valid, rather than testing which of the anatomical proxies used were most appropriate.

Millien and Bovy (2010) re-examined the mass of *Phoberomys* in more detail and concluded the estimated mass of this taxon was highly dependent on the anatomical proxy variable chosen. However, they noted the diameter of the humerus and especially the femur relative to other anatomical variables was unusually high in *P. pattersoni*, whereas other variables like the ratio between tooth row length and humerus or femur length were more consistent with other rodents. This suggests the limb bones of *Phoberomys* were proportionally thicker than that of other rodents, and hence mass estimates based on them were probably overestimates. Instead, they suggest weights of 220–340 kg are more reasonable, about half of initial estimates. However, as Hopkins (2008), Millien (2008), and Millien and Bovy (2010) note, because of the issue of extrapolation error it was difficult to determine whether this was due to these animals having unusually small cheek teeth or unusually thick limb bones.

Engelman (2022) examined the body size of *Phoberomys*, *Josephoartigasia*, and several other giant caviomorphs using skull length, head-body length, and occipital condyle width drawn from a large sample of extant mammals, rather than just rodents. Their three methods produced relatively similar estimated body masses suggesting *Josephoartigasia* was around ~500 kg and *Phoberomys* was around ~150 kg. This suggests that Hopkins (2008), Millien (2008), and Millien and Bovy (2010) were correct in that the original masses for *Phoberomys* and *Josephoartigasia* were overestimates.

And what of *Telicomys*, the “original” South American giant rodent? Analyses suggest this taxon was probably only slightly larger than the modern capybara (90–100 kg for *Telicomys giganteus*, with *T. gigantissumus* being ~20% larger in linear dimensions; Engelman, 2022; Rinderknecht et al., 2019).

1. The giant early-middle Pleistocene *Gigantopithecus blacki* is probably the largest primate to ever exist. However, this species is only known from isolated teeth and a few partial mandibular rami (Zhang & Harrison, 2017), making it difficult to provide an exact estimate of size. Weidenreich (1946) speculated that *Gigantopithecus* was twice the size of a male gorilla. Most studies often cite heights of 2.74–3.66 m (Pei, 1957; Simons & Ettel, 1970; see also references in Zhang & Harrison, 2017) and 225–300 kg (Fleagle, 2013; Simons & Ettel, 1970; see also references in Zhang & Harrison, 2017), with most estimates tending towards ~300 kg. This is an unusually low mass given the estimated height. Many of these studies do not detail how they calculated these estimates, though it seems likely based on wording many were based on simple scaling from gorillas. Zhang and Harrison (2017) calculated body masses for *G. blacki* using m1 area and obtained values of 204 kg (using the equation of Gingerich et al., 1982) and 280 kg (using the equation of Conroy, 1987).

However, even these values may be too large. Johnson (1979) attempted to estimate the limb bone dimensions of *Gigantopithecus* using the depth and breadth of the mandibular ramus and assuming similar proportions to a gorilla. Although a very unusual method of trying to estimate size, Johnson (1979) find their method suggests *Gigantopithecus* may have only been 20-25% larger than gorillas in linear dimensions (which is surprising given mandibular height is expected to overestimate the size of these animals), and furthermore suggest “these fossil primates were probably not the 12 ft ‘giants’ they are often suggested to have been” (Johnson, 1979: p. 587).

Hawley (2019), in trying to reconstruct *G. blacki*, had trouble scaling the known fossils to fit previously reported heights. He created his reconstruction by taking the cranial reconstruction of *Gigantopithecus* created by Grover Krantz (which in turn was based on Mandible III, the largest mandibular specimen of *Gigantopithecus* and inferred to belong to an old male; Simons & Ettel, 1970), and then scaled up an orangutan skeleton (*Gigantopithecus*’ closest living relative) to match. The Krantz skull is somewhat controversial because its morphology is speculative and Krantz’s use of it in cryptozoology, but it provides an approximate value for overall head size in *Gigantopithecus*. This ended up producing a reconstruction with a standing height of approximately 185 cm. Hawley (2019) tried reconstructing the skeleton a number of alternate ways, but could not replicate traditional 2.75–3 m heights, writing “It is possible that my approach was too conservative. Maybe the Krantz reconstruction is too small. Maybe the skull of *Gigantopithecus* was proportionally smaller than that of an orangutan. Maybe *Gigantopithecus* had longer legs. But no matter how you tweak the figures, I don’t think it’s reasonable to reconstruct *Gigantopithecus* with a bipedal standing height of more than about 230 centimetres” (Hawley, 2019: p. 7). This suggests the smaller height of Hawley’s reconstruction is unlikely to be due to the morphology of the Krantz skull. Willoughby (1978: p. 30–31) came to similar conclusions, suggesting *Gigantopithecus* would have had a height of 185 cm and 250 kg if reconstructing this taxon using a gorilla. The only way to produce standing heights of ~3 m for *Gigantopithecus* is if this taxon is reconstructed with proportions similar to a hominin (Willoughby, 1978). Indeed, that may be how these larger height estimates were produced because it was heavily debated in early studies whether *Gigantopithecus* was a hominin or a pongine (Weidenreich, 1946 seems to imply he estimated standing height assuming human-like proportions), later evidence has firmly shown this taxon to be a pongine (Welker et al., 2019).

Another concern is that because *Gigantopithecus* has been suggested to engage in high-fiber herbivory (Simons & Ettel, 1970; but see Zhang & Harrison, 2017) this taxon might be expected to show disproportionally large molars (megadontia) and deep mandibles relative to its overall body size, features that have already been noticed in these fossils. This would suggest that estimates based on molar size and mandible depth, i.e., the only readily measurable features on known remains of *Gigantopithecus*, would be overestimates (Zhang & Harrison, 2017). In the most extensive recent review of this genus, Zhang and Harrison (2017: p. 166) note that without the future discovery of additional skeletal material like postcrania that can be more reliably used to estimate the size of *Gigantopithecus*, it is simply not possible to obtain a reliable size estimate of this species.

# Literature Cited

Accarie, H., Beaudoin, B., Dejax, J., Fries, G., Michard, J. G., & Taquet, P. (1995). Découverte d'un dinosaure théropode nouveau (*Genusaurus sisteronis* n. g., n. sp.) dans l'Albien marin de Sisteron (Alpes de Haute-Provence, France) et extension au Crétacé inférieur de la lignée cératosaurienne. *Comptes rendus de l'Académie des sciences. Série 2. Sciences de la terre et des planètes, 320*(4), 327-334.

Alexander, R. M. (2003). A Rodent as Big as a Buffalo. *Science, 301*(5640), 1678. <https://doi.org/10.1126/science.1090964>

Alvarenga, H. M. F., & Höfling, E. (2003). Systematic revision of the Phorusrhacidae (Aves: Ralliformes). *Papéis Avulsos de Zoologia, 43*(4), 55–91.

Anderson, J. F., Hall-Martin, A., & Russell, D. A. (1985). Long-bone circumference and weight in mammals, birds and dinosaurs. *Journal of Zoology, 207*(1), 53–61. <https://doi.org/10.1111/j.1469-7998.1985.tb04915.x>

Andors, A. V. (1992). Reappraisal of the Eocene groundbird *Diatryma* (Aves: Anserimorphae). In K. E. Campbell, Jr. (Ed.), *Papers in Avian Paleontology Honoring Pierce Brodkorb* (Vol. 36, pp. 109–125). Los Angeles: Natural History Museum of Los Angeles County (Science Series No. 36).

Andres, B., & Langston, W. (2021). Morphology and taxonomy of *Quetzalcoatlus* Lawson 1975 (Pterodactyloidea: Azhdarchoidea). *Journal of Vertebrate Paleontology, 41*(sup1), 46–202. <https://doi.org/10.1080/02724634.2021.1907587>

Andrews, S. M. (1985). Rhizodont crossopterygian fish from the Dinantian of Foulden, Berwickshire, Scotland, with a re-evaluation of this group. *Transactions of the Royal Society of Edinburgh: Earth Sciences, 76*(1), 67–95. <https://doi.org/10.1017/S0263593300010324>

Angst, D., & Buffetaut, E. (2017). *Paleobiology of Giant Flightless Birds*. Oxford: Elsevier.

Angst, D., Buffetaut, E., LÉCuyer, C., Amiot, R., Smektala, F., Giner, S., . . . Martinez, A. (2014). Fossil avian eggs from the Palaeogene of southern France: new size estimates and a possible taxonomic identification of the egg-layer. *Geological Magazine, 152*(1), 70-79. <https://doi.org/10.1017/s0016756814000077>

Anonymous. (2020). Yesterday's Giants? (Life and death of giant insects, part 1/2). Accessed June 11, 2024. <https://lamareauxtetrapodes.wordpress.com/2020/10/12/yesterdays-giants-life-and-death-of-giant-insects-part-1-2/>

Archer, M., Hand, S. J., & Godthelp, H. (1994). Patterns in the history of Australia's mammals and inferences about palaeohabitats. In R. S. Hill (Ed.), *History of the Australian vegetation* (pp. 80–103): Cambridge University Press.

Archer, M., Hand, S. J., Long, J., Schouten, P., & Worthy, T. H. (2023). *Prehistoric Australasia: Visions of Evolution and Extinction*. Clayton: CSIRO Publishing.

Auffenberg, W. (1981). *The behavioral ecology of the Komodo monitor*. Gainesville: University of Florida Press.

Aureliano, T., Ghilardi, A. M., Guilherme, E., Souza-Filho, J. P., Cavalcanti, M., & Riff, D. (2015). Morphometry, bite-force, and paleobiology of the late Miocene caiman *Purussaurus brasiliensis*. *PLOS ONE, 10*(2), e0117944. <https://doi.org/10.1371/journal.pone.0117944>

Banfield, A. W. F., & Novakowski, N. S. (1960). The survival of the wood bison (*Bison bison athabascae* Rhoads) in the Northwest Territories. *Natural History Papers of the National Museum of Canada, 8*, 1–6.

Barker, D. G., Barten, S. L., Ehrsam, J. P., & Daddono, L. (2012). The Corrected Lengths of Two Well-known Giant Pythons and the Establishment of a New Maximum Length Record for Burmese Pythons, *Python bivittatus*. *Bulletin of the Chicago Herpetological Society, 47*(1), 1–6.

Bates, K. T., Falkingham, P. L., Macaulay, S., Brassey, C., & Maidment, S. C. R. (2015). Downsizing a giant: re-evaluating *Dreadnoughtus* body mass. *Biology Letters, 11*(6), 20150215. <https://doi.org/10.1098/rsbl.2015.0215>

Benson, R. B. J., Evans, M., Smith, A. S., Sassoon, J., Moore-Faye, S., Ketchum, H. F., & Forrest, R. (2013). A Giant Pliosaurid Skull from the Late Jurassic of England. *PLOS ONE, 8*(5), e65989. <https://doi.org/10.1371/journal.pone.0065989>

Benton, M. J. (2023). *Dinosaur Behavior: An Illustrated Guide*. Princeton: Princeton University Press.

Benton, M. J., & Harper, D. A. T. (2009). *Introduction to Paleobiology and the Fossil Record* (First ed.). New York: Wiley-Blackwell.

Bianucci, G., Lambert, O., Urbina, M., Merella, M., Collareta, A., Bennion, R., . . . Amson, E. (2023). A heavyweight early whale pushes the boundaries of vertebrate morphology. *Nature, 620*(7975), 824–829. <https://doi.org/10.1038/s41586-023-06381-1>

Bianucci, G., & Landini, W. (2006). Killer sperm whale: a new basal physeteroid (Mammalia, Cetacea) from the Late Miocene of Italy. *Zoological Journal of the Linnean Society, 148*(1), 103-131. <https://doi.org/10.1111/j.1096-3642.2006.00228.x>

Black, R. (2013). Ancient fish downsized but still largest ever. *Washington D.C.* Accessed July 7, 2024. <https://www.nationalgeographic.com/science/article/130827-paleontology-leedsichthys-problematicus-fish-oceans-science>

Bloch, J. I., Rose, K. D., & Gingerich, P. D. (1998). New species of *Batodonoides* (Lipotyphla, Geolabididae) from the early Eocene of Wyoming: Smallest known mammal? *Journal of Mammalogy, 79*(3), 804–827.

Bochaton, C., & Kemp, M. E. (2017). Reconstructing the body sizes of Quaternary lizards using *Pholidoscelis* Fitzinger, 1843, and *Anolis* Daudin, 1802, as case studies. *Journal of Vertebrate Paleontology, 37*(1), e1239626. <https://doi.org/10.1080/02724634.2017.1239626>

Bolaños, W. H., Dias, I. R., & Solé, M. (2024). Zooming in on amphibians: Which is the smallest vertebrate in the world? *Zoologica Scripta, n/a*(n/a). <https://doi.org/10.1111/zsc.12654>

Bonaparte, J., Novas, F. E., & Coria, R. A. (1990). *Carnotaurus sastrei* Bonaparte, the horned, lightly built carnosaur from the Middle Cretaceous of Patagonia. *Contributions in Science, Museum of Natural History, Los Angeles County, 416*, 1-42.

Bonaparte, J. F. (1991). Los vertebrados fósiles de la Formación Río Colorado, de la ciudad de Neuquén y cercanías, Cretácico superior, Argentina. *Revista del Museo Argentino de Ciencias Naturales “Bernardino Rivadavia” e Instituto Nacional de Investigacion de las Ciencias Naturales. Paleontologia, 4*(3), 17-123.

Bonaparte, J. F. (1996). Cretaceous tetrapods of Argentina. *Münchner Geowissenschaftliche Abhandlungen, 30*, 73-130.

Bonaparte, J. F., & Novas, F. E. (1985). *Abelisaurus comahuensis*, n. g., n. sp., Carnosauria from the Late Cretaceous of Patagonia. *Ameghiniana, 21*(2-4), 259-265.

Borths, M. R., & Stevens, N. J. (2019). *Simbakubwa kutokaafrika*, gen. et sp. nov. (Hyainailourinae, Hyaenodonta, ‘Creodonta,’ Mammalia), a gigantic carnivore from the earliest Miocene of Kenya. *Journal of Vertebrate Paleontology, 39*(1), e1570222. <https://doi.org/10.1080/02724634.2019.1570222>

Bourke, J. (2016). T-U-R-T-L-E Power Part 4: The little-known paleobiology of the world’s largest tortoise. Accessed July 10, 2024. <https://reptilis.net/2016/05/08/t-u-r-t-l-e-power-part-4-the-little-known-paleobiology-of-the-worlds-largest-tortoise/>

Boylan, J. C., & Murphy, P. A. (1978). The ventral armor and feeding biomechanics of *Glyptaspis verrucosa* Newberry, a placoderm from the Fammenian Cleveland Shale. *American Museum Novitates, 2655*, 1–12.

Braddy, S. J. (2023). Pterygotid eurypterid palaeoecology: praedichnia and palaeocommunities. *Bulletin of Geosciences, 98*(4), 289–302. <https://doi.org/10.3140/bull.geosci.1891>

Braddy, S. J., Poschmann, M., & Tetlie, O. E. (2007). Giant claw reveals the largest ever arthropod. *Biology Letters, 4*(1), 106–109. <https://doi.org/10.1098/rsbl.2007.0491>

Brassey, C. A., Maidment, S. C. R., & Barrett, P. M. (2015). Body mass estimates of an exceptionally complete *Stegosaurus* (Ornithischia: Thyreophora): comparing volumetric and linear bivariate mass estimation methods. *Biology Letters, 11*(3), 20140984. <https://doi.org/10.1098/rsbl.2014.0984>

Briggs, D. E. G. (1972). *Anomalocaris*, the largest known Cambrian arthropod. *Palaeontology, 22*(3), 631–644.

Brochu, C. A. (2003). Osteology of *Tyrannosaurus rex*: Insights from a nearly complete Skeleton and High-Resolution Computed Tomographic Analysis of the Skull. *Journal of Vertebrate Paleontology, 22*(sup4), 1-138. <https://doi.org/10.1080/02724634.2003.10010947>

Brown, B. (1931). The Largest Known Land Tortoise. *Natural History, 31*, 184–187.

Brown, P., Sutikna, T., Morwood, M. J., Soejono, R. P., Jatmiko, Wayhu Saptomo, E., & Awe Due, R. (2004). A new small-bodied hominin from the Late Pleistocene of Flores, Indonesia. *Nature, 431*(7012), 1055–1061. <https://doi.org/10.1038/nature02999>

Brusatte, S. L. (2001). Re: Bruhathkayosaurus. *Dinosaur Mailing List*. Accessed July 27, 2024. <https://web.archive.org/web/20090107210722/http://dml.cmnh.org/2001Jun/msg00618.html>

Buchy, M.-C. l., Frey, E., Stinnesbeck, W., & López-Oliva, J. G. (2003). First occurrence of a gigantic pliosaurid plesiosaur in the late Jurassic (Kimmeridgian) of Mexico. *Bulletin de la Société Géologique de France, 174*(3), 271–278. <https://doi.org/10.2113/174.3.271>

Buffetaut, E., & Angst, D. (2013). “Terror cranes” or peaceful plant-eaters: changing interpretations of the palaeobiology of gastornithid birds. *Revue de Paléobiologie, Genève, 32*(2), 413–422.

Buffetaut, E., Grigorescu, D., & Csiki, Z. (2003). Giant azhdarchid pterosaurs from the terminal Cretaceous of Transylvania (western Romania). *Geological Society, London, Special Publications, 217*(1), 91–104. <https://doi.org/10.1144/gsl.Sp.2003.217.01.09>

Burger, J. R., George, M. A., Jr., Leadbetter, C., & Shaikh, F. (2019). The allometry of brain size in mammals. *Journal of Mammalogy, 100*(2), 276–283. <https://doi.org/10.1093/jmammal/gyz043>

Cadena, E. A., Scheyer, T. M., Carrillo-Briceño, J. D., Sánchez, R., Aguilera-Socorro, O. A., Vanegas, A., . . . Sánchez-Villagra, M. R. (2020). The anatomy, paleobiology, and evolutionary relationships of the largest extinct side-necked turtle. *Science Advances, 6*(7), eaay4593. <https://doi.org/10.1126/sciadv.aay4593>

Calvo, J. O. (2006). *Dinossauros e fauna associada de uma nova localidade no Lago Barreales (Formação Portezuelo, Cretáceo Superior), Neuquén, Argentina.* UFRJ/Museu Nacional/Programa de Pós-graduação em Ciências Biológicas, Rio de Janeiro.

Calvo, J. O., Juarez Valieri, R. D., & Porfiri, J. D. (2008). *Re-sizing giants: estimation of body length of Futalognkosaurus dukei and implications for giant titanosaurian sauropods*. Paper presented at the III Congreso Latinoamericano de Paleontología de Vertebrados, Neuquén.

Calvo, J. O., Porfiri, J. D., Gonzalez-Riga, B. J., & Kellner, A. W. (2007). A new Cretaceous terrestrial ecosystem from Gondwana with the description of a new sauropod dinosaur. *Anais da Academia Brasileira Ciências, 79*(3), 529–541. <https://doi.org/10.1590/s0001-37652007000300013>

Calvo, J. O., Rubilar-Rogers, D., & Moreno, K. (2004). A new Abelisauridae (Dinosauria: Theropoda) from northwest Patagonia. *Ameghiniana, 41*(4), 555-563. <https://doi.org/10.11646/zootaxa.2450.1.1>

Camp, C. L. (1976). Vorläufige Mitteilung über große Ichthyosaurier aus der oberen Trias von Nevada. *Sitzungsberichte der Österreichischen Akademie der Wissenschaften, Mathematisch-naturwissenschaftliche Klasse, Abteilung I., 185*, 125–134.

Camp, C. L. (1980). Large ichthyosaurs from the upper Triassic of Nevada. *Palaeontographica Abteilung A, 170*(4-6), 139–200.

Campbell, K. E., & Marcus, L. (1992). The relationship of hindlimb bone dimensions to body weight in birds. *Scientific Series of the Natural History Museum of Los Angeles County, 36*, 395–412.

Campbell, K. E., & Tonni, E. P. (1980). A new genus of teratorn from the Huayquerian of Argentina. *Contributions in Science, Museum of Natural History, Los Angeles County, 330*, 59–68.

Campbell, K. E., & Tonni, E. P. (1983). Size and Locomotion in Teratorns (Aves: Teratornithidae). *The Auk, 100*(2), 390–403.

Campione, N. E. (2017). Extrapolating body masses in large terrestrial vertebrates. *Paleobiology, 43*(4), 693–699. <https://doi.org/10.1017/pab.2017.9>

Campione, N. E., & Evans, D. C. (2012). A universal scaling relationship between body mass and proximal limb bone dimensions in quadrupedal terrestrial tetrapods. *BMC Biology, 10*(1), 60. <https://doi.org/10.1186/1741-7007-10-60>

Campione, N. E., & Evans, D. C. (2020). The accuracy and precision of body mass estimation in non-avian dinosaurs. *Biol Rev Camb Philos Soc, 95*(6), 1759–1797. <https://doi.org/10.1111/brv.12638>

Canale, J. I., Scanferla, C. A., Agnolin, F. L., & Novas, F. E. (2009). New carnivorous dinosaur from the Late Cretaceous of NW Patagonia and the evolution of abelisaurid theropods. *Naturwissenschaften, 96*(3), 409-414. <https://doi.org/10.1007/s00114-008-0487-4>

Cannell, A. E. R. (2018). The engineering of the giant dragonflies of the Permian: revised body mass, power, air supply, thermoregulation and the role of air density. *Journal of Experimental Biology, 221*(19), jeb185405. <https://doi.org/10.1242/jeb.185405>

Carbone, C., Mace, G. M., Roberts, S. C., & Macdonald, D. W. (1999). Energetic constraints on the diet of terrestrial carnivores. *Nature, 402*(6759), 286–288. <https://doi.org/10.1038/46266>

Carpenter, K. (2006). Biggest of the big—a critical re-evaluation of the megasauropod *Amphicoelias fragillimus*. *New Mexico Museum of Natural History and Science Bulletin, 36*, 131–137.

Carpenter, K. (2018). *Maraapunisaurus fragillimus*, N.G. (formerly *Amphicoelias fragillimus*), a basal Rebbachisaurid from the Morrison Formation (Upper Jurassic) of Colorado. *Geology of the Intermountain West, 5*, 227–244. <https://doi.org/10.31711/giw.v5.pp227-244>

Carrano, M. T., Loewen, M. A., & Sertich, J. J. W. (2001). New materials of *Masiakasaurus knopfleri* Sampson, Carrano, and Forster 2001, and implications for the morphology of the Noasauridae. *Smithsonian Contributions to Paleontology, 95*, 1-53.

Chambers, P., Cooper, L., Green, N., Haines, T., James, J., Landis, T. B., . . . Reed, S. (1999). Walking with Dinosaurs. In T. Haines, J. James, & M. C. Bacquet (Eds.), *Cruel Sea* (Vol. 1): BBC.

Chatterjee, S. (2015). *The Rise of Birds: 225 Milliona Years of Evolution* (Second ed.). Baltimore: John Hopkins University Press.

Chatterjee, S., & Templin, R. J. (2004). Posture, locomotion and palaeoecology of pterosaurs. *Geological Society of America Special Publication, 376*, 1-64.

Chatterjee, S., Templin, R. J., & Campbell, K. E. (2007). The aerodynamics of Argentavis, the world's largest flying bird from the Miocene of Argentina. *Proceedings of the National Academy of Sciences, 104*(30), 12398–12403. <https://doi.org/10.1073/pnas.0702040104>

Chen, J.-y., Ramsköld, L., & Zhou, G.-q. (1994). Evidence for Monophyly and Arthropod Affinity of Cambrian Giant Predators. *Science, 264*(5163), 1304–1308. <https://doi.org/10.1126/science.264.5163.1304>

Chiappe, L. M., & Bertelli, S. (2006). Skull morphology of giant terror birds. *Nature, 443*(7114), 929.

Chlupáĉ, I. (1994). Pterygotid eurypterids (Arthropoda, Chelicerata) in the Silurian and Devonian of Bohemia. *Journal of the Czech Geological Society, 39*, 147–162.

Christiansen, P. (1999). What size were *Arctodus simus* and *Ursus spelaeus* (Carnivora: Ursidae)? *Annales Zoologici Fennici, 36*(2), 93–102.

Christiansen, P., & Bonde, N. (2002). A New Species of Gigantic Mosasaur from the Late Cretaceous of Israel. *Journal of Vertebrate Paleontology, 22*(3), 629–644.

Christiansen, P., & Harris, J. M. (2005). Body size of *Smilodon* (Mammalia: Felidae). *Journal of Morphology, 266*(3), 369–384. <https://doi.org/10.1002/jmor.10384>

Cidade, G. M., Riff, D., & Hsiou, A. S. (2019). The feeding habits of the strange crocodylian *Mourasuchus* (Alligatoroidea, Caimaninae): a review, new hypotheses and perspectives. *Revista Brasileira De Paleontologia, 22*(2), 106–119. <https://doi.org/10.4072/rbp.2019.2.03>

Claeson, K. M., Sidlauskas, B. L., Troll, R., Prescott, Z. M., & Davis, E. B. (2024). From sabers to spikes: A newfangled reconstruction of the ancient, giant, sexually dimorphic Pacific salmon, †*Oncorhynchus rastrosus* (Salmoninae: Salmonini). *PLOS ONE, 19*(4), e0300252. <https://doi.org/10.1371/journal.pone.0300252>

Clapham, M. E., & Karr, J. A. (2012). Environmental and biotic controls on the evolutionary history of insect body size. *Proceedings of the National Academy of Sciences, 109*(27), 10927-10930. <https://doi.org/10.1073/pnas.1204026109>

Cleary, T. J., Benson, R. B. J., Evans, S. E., & Barrett, P. M. (2018). Lepidosaurian diversity in the Mesozoic-Palaeogene: the potential roles of sampling biases and environmental drivers. *R Soc Open Sci, 5*(3), 171830. <https://doi.org/10.1098/rsos.171830>

Colbert, E. H., & Bird, R. T. (1954). A gigantic crocodile from the Upper Cretaceous beds of Texas. *American Museum Novitates, 1688*, 1–22.

Conroy, G. C. (1987). Problems of body-weight estimation in fossil primates. *International Journal of Primatology, 8*(2), 115–137. <https://doi.org/10.1007/BF02735160>

Cooper, J. A., Hutchinson, J. R., Bernvi, D. C., Cliff, G., Wilson, R. P., Dicken, M. L., . . . Pimiento, C. (2022). The extinct shark *Otodus megalodon* was a transoceanic superpredator: Inferences from 3D modeling. *Science Advances, 8*(33), eabm9424. <https://doi.org/10.1126/sciadv.abm9424>

Cooper, J. A., Pimiento, C., Ferrón, H. G., & Benton, M. J. (2020). Body dimensions of the extinct giant shark *Otodus megalodon*: a 2D reconstruction. *Scientific Reports, 10*(1), 14596. <https://doi.org/10.1038/s41598-020-71387-y>

Cope, E. D. (1878). A new species of *Amphicoelias*. *American Naturalist, 12*, 563–565.

Coria, R. A., Chiappe, L. M., & Dingus, L. (2002). A new close relative of *Carnotaurus sastrei* Bonaparte 1985 (Theropoda: Abelisauridae) from the Late Cretaceous of Patagonia. *Journal of Vertebrate Paleontology, 22*(2), 460-465. [https://doi.org/10.1671/0272-4634(2002)022[0460:ANCROC]2.0.CO;2](https://doi.org/10.1671/0272-4634(2002)022%5b0460:ANCROC%5d2.0.CO;2)

Cox, C., & Hutchinson, P. (1991). Fishes and amphibians from the late Permian Pedra de Fogo Formation of northern Brazil. *Palaeontology, 34*(3), 561–573.

Curtice, B. (1996). *Codex of diplodocid caudal vertebrae from the Dry Mesa Dinosaur Quarry.* (M.S.), Brigham Young University, Provo.

Dal Sasso, C., Maganuco, S., Buffetaut, E., & Mendez, M. A. (2005). New information on the skull of the enigmatic theropod *Spinosaurus*, with remarks on its size and affinities. *Journal of Vertebrate Paleontology, 25*(4), 888–896. [https://doi.org/10.1671/0272-4634(2005)025[0888:NIOTSO]2.0.CO;2](https://doi.org/10.1671/0272-4634(2005)025%5b0888:NIOTSO%5d2.0.CO;2)

Damiani, R., & Steyer, J. S. b. (2005). A giant brachyopoid temnospondyl from the Upper Triassic or Lower Jurassic of Lesotho. *Bulletin de la Société Géologique de France, 176*(3), 243–248. <https://doi.org/10.2113/176.3.243>

Damuth, J., & MacFadden, B. J. (1990). *Body Size in Mammalian Paleobiology: Estimation and Biological Implications*. Cambridge, United Kingdom: Cambridge University Press.

Datta, D., & Bajpai, S. (2024). Largest known madtsoiid snake from warm Eocene period of India suggests intercontinental Gondwana dispersal. *Scientific Reports, 14*(1), 8054. <https://doi.org/10.1038/s41598-024-58377-0>

Davies, N. S., Garwood, R. J., McMahon, W. J., Schneider, J. W., & Shillito, A. P. (2022). The largest arthropod in Earth history: insights from newly discovered *Arthropleura* remains (Serpukhovian Stainmore Formation, Northumberland, England). *Journal of the Geological Society, 179*(3), jgs2021–2115. <https://doi.org/10.1144/jgs2021-115>

De Maddalena, A., Glaizot, O., & Olivier, G. (2003). On the Great White Shark, *Carcharodon carcharias* (Linnaeus, 1758), preserved in the Museum of Zoology in Lausanne. *Marine Life, 13*(1-2), 53–59.

Dean, B. (1909). Notes on a newly mounted *Titanichthys*. *Memoirs of the American Museum of Natural History, 9*(5), 270–271.

Deeming, D. C. (2022). Higher level taxonomy affects body mass and femur length as predictors for egg size in birds. *Ornis Hungarica, 30*(1), 21-29. <https://doi.org/10.2478/orhu-2022-0002>

Defler, T. (2018). *History of Terrestrial Mammals in South America: How South American Mammalian Fauna Changed from the Mesozoic to Recent Times*. New York: Springer.

Denison, R. H. (1978). Placodermi. In H.-P. Schultze (Ed.), *Handbook of Palaeoichthyology* (Vol. 2, pp. 1–128). Stuttgart: Gustav Fischer Verlag.

Dorrington, G. E. (2016). Heavily loaded flight and limits to the maximum size of dragonflies (Anisoptera) and griffenflies (Meganisoptera). *Lethaia, 49*, 261–274.

Driscoll, D. A., Dunhill, A. M., Stubbs, T. L., & Benton, M. J. (2019). The mosasaur fossil record through the lens of fossil completeness. *Palaeontology, 62*(1), 51–75. <https://doi.org/10.1111/pala.12381>

Dubied, M., Solé, F., & Mennecart, B. (2019). The cranium of *Proviverra typica* (Mammalia, Hyaenodonta) and its impact on hyaenodont phylogeny and endocranial evolution. *Palaeontology, 62*(6), 983–1001. <https://doi.org/10.1111/pala.12437>

Dyke, G. J., & Kaiser, G. W. (2010). Cracking a developmental constraint: Egg size and bird evolution. *Records of the Australian Museum, 62*, 207-216.

Egi, N. (2001). Body mass estimates in extinct mammals from limb bone dimensions: the case of North American hyaenodontids. *Palaeontology, 44*(3), 497–528. <https://doi.org/10.1111/1475-4983.00189>

Ellers, O., Gordon, C. M., Hukill, M. T., Kukaj, A., Cannell, A., & Nel, A. (2024). Induced Power Scaling Alone Cannot Explain Griffenfly Gigantism. *Integr Comp Biol*. <https://doi.org/10.1093/icb/icae046>

Elliott, D. K., Irmis, R. B., Hansen, M. C., & Olson, T. J. (2004). Chondrichthyans from the Pennsylvanian (Desmoinesian) Naco Formation of central Arizona. *Journal of Vertebrate Paleontology, 24*(2), 268–280. <https://doi.org/10.1671/1978>

Engelman, R. K. (2020). Estimating body mass in extinct therian mammals using width of the occipital condyles. *Journal of Vertebrate Paleontology, SVP Program and Abstracts Book 2020*, 133.

Engelman, R. K. (2022). Resizing the largest known extinct rodents (Caviomorpha: Dinomyidae, Neoepiblemidae) using occipital condyle width. *Royal Society Open Science, 9*(6), 220370. <https://doi.org/10.1098/rsos.220370>

Engelman, R. K. (2023a). A Devonian fish tale: A new method of body length estimation suggests much smaller sizes for *Dunkleosteus terrelli* (Placodermi: Arthrodira). *Diversity, 15*(3), 318. <https://doi.org/10.3390/d15030318>

Engelman, R. K. (2023b). Giant, swimming mouths: Oral dimensions of extant sharks do not accurately predict body size in *Dunkleosteus terrelli* (Placodermi: Arthrodira). *PeerJ, 11*, 1–34. <https://doi.org/10.7717/peerj.15131>

Engelman, R. K. (In Press). Reconstructing *Dunkleosteus terrelli* (Placodermi: Arthrodira): A New Look for an Iconic Devonian Predator. *Palaeontologia Electronica*.

Erickson, G. M., & Brochu, C. A. (1999). How the ‘terror crocodile’ grew so big. *Nature, 398*(6724), 205–206. <https://doi.org/10.1038/18343>

Evans, G., Haines, T., Kemp, A., Smith, G., & Chambers, P. (2005). Walking With Monsters. In C. Leland & T. Haines (Eds.), *Water Dwellers*: BBC.

Evans, S. E., Groenke, J. R., Jones, M. E., Turner, A. H., & Krause, D. W. (2014). New material of *Beelzebufo*, a hyperossified frog (Amphibia: Anura) from the late cretaceous of Madagascar. *PLOS ONE, 9*(1), e87236. <https://doi.org/10.1371/journal.pone.0087236>

Evans, S. E., Jones, M. E. H., & Krause, D. W. (2008). A giant frog with South American affinities from the Late Cretaceous of Madagascar. *Proceedings of the National Academy of Sciences, 105*(8), 2951–2956. <https://doi.org/10.1073/pnas.0707599105>

Everhart, M. J., Jagt, J. W. M., Mulder, E. W. A., & Schulp, A. S. (2016). *Mosasaurs - How large did they really get?* Paper presented at the 5th Triennial Mosasaur Meeting—A Global Perspective on Mesozoic Marine Amniotes, Uppsala.

Ezcurra, M. D., Agnolin, F. L., & Novas, F. E. (2010). An abelisauroid dinosaur with a non-atrophied manus from the Late Cretaceous Pari Aike Formation of southern Patagonia. *Zootaxa, 2450*(1), 1-25. <https://doi.org/10.11646/zootaxa.2450.1.1>

Falconer, H., & Cautley, P. T. (1837). On Additional Fossil Species of the Order Quadrumana from the Siwalik Hills. *Journal of the Asiatic Society of Bengal, 6*, 354–360.

Falconer, H., & Cautley, P. T. (1844). Communication on the *Colossochelys atlas*, A Fossil Tortoise of Enormous Size from the Tertiary Strata of the Siwalk Hills in the North of India. *Proceedings of the Zoological Society of London, 12*, 54–84.

Falvey, J., Fothergill, A., Frank, D., Scholey, K., Spielberg, S., Sverdrup, G., & Tapster, D. (2023). Life on Our Planet. In *The First Frontier*: Netflix.

Fanti, F., Cau, A., & Negri, A. (2014). A giant mosasaur (Reptilia, Squamata) with an unusually twisted dentition from the Argille Scagliose Complex (late Campanian) of Northern Italy. *Cretaceous Research, 49*, 91–104. <https://doi.org/10.1016/j.cretres.2014.01.003>

Fanti, F., Miyashita, T., Cantelli, L., Mnasri, F., Dridi, J., Contessi, M., & Cau, A. (2016). The largest thalattosuchian (Crocodylomorpha) supports teleosaurid survival across the Jurassic-Cretaceous boundary. *Cretaceous Research, 61*, 263–274. <https://doi.org/10.1016/j.cretres.2015.11.011>

Farke, A. A., & Sertich, J. J. W. (2013). An abelisauroid theropod dinosaur from the Turonian of Madagascar. *PLOS ONE, 8*(4), e62047. <https://doi.org/10.1371/journal.pone.0062047>

Farlow, J. O., Hurlburt, G. R., Elsey, R. M., Britton, A. R. C., & Langston Jr, W. (2005). Femoral dimensions and body size of Alligator mississippiensis: estimating the size of extinct mesoeucrocodylians. *Journal of Vertebrate Paleontology, 25*(2), 354–369. [https://doi.org/10.1671/0272-4634(2005)025[0354:FDABSO]2.0.CO;2](https://doi.org/10.1671/0272-4634(2005)025%5b0354:FDABSO%5d2.0.CO;2)

Ferreira, G. S., Nascimento, E. R., Cadena, E. A., Cozzuol, M. A., Farina, B. M., Pacheco, M. L. A. F., . . . Langer, M. C. (2024). The latest freshwater giants: a new *Peltocephalus* (Pleurodira: Podocnemididae) turtle from the Late Pleistocene of the Brazilian Amazon. *Biology Letters, 20*(3), 20240010. <https://doi.org/10.1098/rsbl.2024.0010>

Ferrón, H. G., Martinez-Perez, C., & Botella, H. (2017). Ecomorphological inferences in early vertebrates: reconstructing *Dunkleosteus terrelli* (Arthrodira, Placodermi) caudal fin from palaeoecological data. *PeerJ, 5*, e4081. <https://doi.org/10.7717/peerj.4081>

Figueirido, B., Pérez-Claros, J. A., Torregrosa, V., Martín-Serra, A., & Palmqvist, P. (2010). Demythologizing *Arctodus simus*, the ‘short-faced’ long-legged and predaceous bear that never was. *Journal of Vertebrate Paleontology, 30*(1), 262–275. <https://doi.org/10.1080/02724630903416027>

Filippi, L. S., Méndez, A. H., Juárez Valieri, R. D., & Garrido, A. C. (2016). A new brachyrostran with hypertrophied axial structures reveals an unexpected radiation of latest Cretaceous abelisaurids. *Cretaceous Research, 61*, 209-219. <https://doi.org/10.1016/j.cretres.2015.12.018>

Flannery, T. (2008). *Chasing Kangaroos: A Continent, a Scientist, and a Search for the World's Most Extraordinary Creature*. New York: Grove Press.

Fleagle, J. G. (2013). *Primate Adaptation and Evolution* (Third Edition ed.).

Flink, T., Cote, S., Rossie, J. B., Kibii, J. M., & Werdelin, L. (2021). The neurocranium of *Ekweeconfractus amorui* gen. et sp. nov. (Hyaenodonta, Mammalia) and the evolution of the brain in some hyaenodontan carnivores. *Journal of Vertebrate Paleontology, 41*(2), e1927748. <https://doi.org/10.1080/02724634.2021.1927748>

Flower, R. H. (1955). Status of Endoceroid Classification. *Journal of Paleontology, 29*(3), 329–371.

Forrest, R. (2009). Comment on "Sea Dragons of Avalon: A 2009 seminar". *Tetrapod Zoology*. Accessed July 14, 2024.

Fortelius, M., & Kappelman, J. (1993). The largest land mammal ever imagined. *Zoological Journal of the Linnean Society, 108*(1), 85–101. <https://doi.org/10.1111/j.1096-3642.1993.tb02560.x>

Frey, E., & Martill, D. M. (1996). A reappraisal of *Arambourgiania* (Pterosauria, Pterodactyloidea): One of the world's largest flying animals. *Neues Jahrbuch für Geologie und Paläontologie - Abhandlungen, 199*(2), 221–247. <https://doi.org/10.1127/njgpa/199/1996/221>

Fuchs, D., Iba, Y., Heyng, A., Iijima, M., Klug, C., Larson, N. L., & Schweigert, G. (2020). The Muensterelloidea: phylogeny and character evolution of Mesozoic stem octopods. *Papers in Palaeontology, 6*(1), 31–92. <https://doi.org/10.1002/spp2.1254>

Galton, P. M., & Ayyasami, K. (2017). Purported latest bone of a plated dinosaur (Ornithischia: Stegosauria), a "dermal plate" from the Maastrichtian (Upper Cretaceous) of southern India. *Neues Jahrbuch für Geologie und Paläontologie - Abhandlungen, 285*(1), 91-96. <https://doi.org/10.1127/njgpa/2017/0671>

Gess, R. W., & Ahlberg, P. E. (2023). A high latitude Gondwanan species of the Late Devonian tristichopterid *Hyneria* (Osteichthyes: Sarcopterygii). *PLOS ONE, 18*(2), e0281333. <https://doi.org/10.1371/journal.pone.0281333>

Gianechini, F. A., Méndez, A. H., Filippi, L. S., Paulina-Carabajal, A., Juárez-Valieri, R. D., & Garrido, A. C. (2020). A new furileusaurian abelisaurid from La Invernada (Upper Cretaceous, Santonian, Bajo de la Carpa Formation), northern Patagonia, Argentina. *Journal of Vertebrate Paleontology, 40*(6), e1877151. <https://doi.org/10.1080/02724634.2020.1877151>

Gillette, D. D. (1991). *Seismosaurus halli*, gen. et sp. nov., a new sauropod dinosaur from the Morrison Formation (Upper Jurassic/Lower Cretaceous) of New Mexico, USA. *Journal of Vertebrate Paleontology, 11*(4), 417–433. <https://doi.org/10.1080/02724634.1991.10011413>

Gillette, D. D. (1994). *Seismosaurus, the Earth Shaker*. New York: Columbia University Press.

Gingerich, P. D., Smith, B. H., & Rosenberg, K. (1982). Allometric scaling in the dentition of primates and prediction of body weight from tooth size in fossils. *American Journal of Physical Anthropology, 58*(1), 81–100. <https://doi.org/10.1002/ajpa.1330580110>

Ginsburg, L. (1980). *Hyainailouros sulzeri*, mammifère créodonte du Miocène d'Europe. *Annales de Paléontologie, 66*(1), 19–73.

Ginter, M. (2010). Teeth of late Famennian ctenacanth sharks from the Cleveland Shale. In D. K. Elliott, J. G. Maisey, X. Yu, & D. Miao (Eds.), *Morphology, Phylogeny and Paleobiogeography of Fossil Fishes* (pp. 145–158). München: Verlag Dr. Friedrich Pfeil.

Gould, S. J. (1987). The case of the creeping fox terrier clone. *Natural History, 97*, 16–24.

Granger, W., & Gregory, W. K. (1935). A revised restoration of the skeleton of *Baluchitherium*, gigantic fossil rhinoceros of Central Asia. *American Museum Novitates, 787*, 1–3.

Granger, W., & Gregory, W. K. (1936). Further notes on the giant extinct rhinoceros, *Baluchitherium*, from the Oligocene of Mongolia. *Bulletin of the American Museum of Natural History, 72*, 1–73.

Greenfield, T. (2020a). Fact-checking *Planet Dinosaur*'s *Onchopristis*. Accessed June 11, 2024. <https://incertaesedisblog.wordpress.com/2022/04/12/fact-checking-planet-dinosaurs-onchopristis/>

Greenfield, T. (2020b). Reconstructing fossil cephalopods: *Enchoteuthis* (“*Tusoteuthis*” ). Accessed July 11, 2024.

Greenfield, T. (2023). Chased by Sea Monsters’ “giant orthocone”: *Endoceras*, not *Cameroceras*. Accessed July 6, 2024. <https://incertaesedisblog.wordpress.com/2023/07/19/chased-by-sea-monsters-giant-orthocone-endoceras-not-cameroceras/>

Greer, A. E. (1974). On the Maximum Total Length of the Salt-Water Crocodile (*Crocodylus porosus*). *Journal of Herpetology, 8*(4), 381–384. <https://doi.org/10.2307/1562913>

Grigg, G., & Kirshner, D. (2015). *Biology and evolution of crocodilians*. Clayton: CSIRO Publishing.

Grigoriev, D. V. (2014). Giant *Mosasaurus hoffmanni* (Squamata, Mosasauridae) from the late Cretaceous (Maastrictian) of Penza, Russia. *Proceedings of the Zoological Institute RAS, 318*(2), 148–167.

Grillo, O. N., & Delcourt, R. (2017). Allometry and body length of abelisauroid theropods: *Pycnonemosaurus nevesi* is the new king. *Cretaceous Research, 69*, 71-89. <https://doi.org/10.1016/j.cretres.2016.09.001>

Grimaldi, D., & Engel, M. S. (2005). *Evolution of the Insects*. Cambridge: Cambridge University Press.

Gromova, V. I. (1959). Gigantskie nosorogi. *Trudy Paleontologicheskogo Instituta Akademya Nauk USSR, 71*, 1–164.

Haas, S. K., Hayssen, V., & Krausman, P. R. (2005). *Panthera leo*. *Mammalian Species, 762*, 1–11. [https://doi.org/10.1644/1545-1410(2005)762[0001:PL]2.0.CO;2](https://doi.org/10.1644/1545-1410(2005)762%5b0001:PL%5d2.0.CO;2)

Halloran, A. F. (1961). American bison weights and measurements from the Wichita Mountain Wildlife Refuge. *Proceedings of the Oklahoma Academy of Science, 41*, 212–218.

Handley, W. D., Chinsamy, A., Yates, A. M., & Worthy, T. H. (2016). Sexual dimorphism in the late Miocene mihirung *Dromornis stirtoni* (Aves: Dromornithidae) from the Alcoota Local Fauna of central Australia. *Journal of Vertebrate Paleontology, 36*(5). <https://doi.org/10.1080/02724634.2016.1180298>

Hansen, M. C. (1996). Phylum Chordata-vertebrate fossils. In R. M. Feldmann & M. Hackathorn (Eds.), *Fossils of Ohio* (Vol. Bulletin 70, pp. 288–369). Columbus: Ohio Division of Geological Survey.

Harris, M. P. (1964). Aspects of the breeding biology of the gulls *Larus argentatus*, *L. fuscus* and *L. marinus*. *Ibis, 106*(4), 432–456. <https://doi.org/10.1111/j.1474-919X.1964.tb03725.x>

Hart, L. J., Campione, N. E., & McCurry, M. R. (2022). On the estimation of body mass in temnospondyls: a case study using the large-bodied *Eryops* and *Paracyclotosaurus*. *Palaeontology, 65*(6), e12629. <https://doi.org/10.1111/pala.12629>

Hartman, S. (2013). Mass estimates: North versus south redux. *Dr. Scott Hartman's Skeletal Drawing.com*. Accessed March 24, 2023. <https://www.skeletaldrawing.com/home/mass-estimates-north-vs-south-redux772013>

Hartman, S. (2014). There's something fishy about the new *Spinosaurus*. *Dr. Scott Hartman's Skeletal Drawing.com*. Accessed July 14, 2024. <https://www.skeletaldrawing.com/home/theres-something-fishy-about-spinosaurus9112014>

Hawley, R. J. (2019). Questions and Answers - *Gigantopithecus*. *Tate Geological Museum 23*(3), 7.

Head, J. J. (2013). 'Lizard King' fossil shows giant reptiles coexisted with mammals during globally warm past. Accessed July 11, 2024. <https://www.eurekalert.org/news-releases/792176>

Head, J. J., Bloch, J. I., Hastings, A. K., Bourque, J. R., Cadena, E. A., Herrera, F. A., . . . Jaramillo, C. A. (2009). Giant boid snake from the Palaeocene neotropics reveals hotter past equatorial temperatures. *Nature, 457*(7230), 715–717. <https://doi.org/10.1038/nature07671>

Head, J. J., Gunnell, G. F., Holroyd, P. A., Hutchison, J. H., & Ciochon, R. L. (2013). Giant lizards occupied herbivorous mammalian ecospace during the Paleogene greenhouse in Southeast Asia. *Proceedings of the Royal Society B: Biological Sciences, 280*(1763), 20130665. <https://doi.org/10.1098/rspb.2013.0665>

Hébert, E. (1855). Note sur le fémur de *Gastornis parisiensis*. *Comptes rendus de l'Académie des Sciences, 40*, 1214-1217.

Hecht, M. K. (1975). The morphology and relationships of the largest known terrestrial lizard, *Megalania prisca* Owen, from the Pleistocene of Australia. *Proceedings of the Royal Society of Victoria, 87*(2), 239–250.

Hector, J. (1872). Further notice of bones of a fossil penguin (*Palaeeudyptes antarcticus*, Huxley). *Transactions and Proceedings of the Royal Society of New Zealand, 5*, 438–439.

Helgen, K. M., Wells, R. T., Kear, B. P., Gerdtz, W. R., & Flannery, T. F. (2006). Ecological and evolutionary significance of sizes of giant extinct kangaroos. *Australian Journal of Zoology, 54*(4), 293–303. <https://doi.org/10.1071/ZO05077>

Henderson, D. M. (2010). Pterosaur body mass estimates from three-dimensional mathematical slicing. *Journal of Vertebrate Paleontology, 30*(3), 768–785. <https://doi.org/10.1080/02724631003758334>

Herne, M. C., & Lucas, S. G. (2006). *Seismosaurus hallorum*: Osteological reconstruction from the holotype. *New Mexico Museum of Natural History and Science Bulletin, 36*, 139–148.

Hillman-Smith, A. K. K., & Groves, C. (1994). *Diceros bicornis*. *Mammalian Species, 455*, 1–8.

Hirayama, R., Sonoda, T., Takai, M., Htike, T., Thein, Z. M. M., & Takahashi, A. (2015). *Megalochelys*: gigantic tortoise from the Neogene of Myanmar. *PeerJ PrePrints*, e961v961.

Hodnett, J.-P. M., Grogan, E. M., Lund, R., Lucas, S. G., Suazo, T., Elliott, D. K., & Pruitt, J. (2021). Ctenacanthiform sharks from the late Pennsylvanian (Missourian) Tinajas Member of the Atrasado Formation, central New Mexico. *New Mexico Museum of Natural History and Science Bulletin, 84*, 391–424.

Hodnett, J.-P. M., Toomey, R., Egli, H. C., Ward, G., Wood, J. R., Olson, R., . . . Santucci, V. L. (2023). New ctenacanth sharks (Chondrichthyes; Elasmobranchii; Ctenacanthiformes) from the Middle to Late Mississippian of Kentucky and Alabama. *Journal of Vertebrate Paleontology, 43*(3), e2292599. <https://doi.org/10.1080/02724634.2023.2292599>

Hodnett, J.-P. M., Toomey, R., Olson, R., Tolleson, K., Boldon, R., Wood, J., . . . Santucci, V. L. (2024). Sharks in the dark: Paleontological resource inventory reveals multiple successive Mississippian Subperiod cartilaginous fish (Chondrichthyes) assemblages within Mammoth Cave National Park, Kentucky. *Parks Stewardship Forum, 40*(1). <https://doi.org/10.5070/p540162921>

Hodnett, J.-P. M., Tweet, J. S., & Santucci, V. L. (2022). The occurrence of fossil cartilaginous fishes (Chondrichthyes) within the parks and monuments of the National Parks Service. *New Mexico Museum of Natural History and Science Bulletin, 90*, 183–208.

Hoganson, J. W. (2010). Tusoteuthis longa: North Dakota's 80 million-year-old giant squid. *Geo News, 37*(1), 1–4.

Hoganson, J. W. (2014). Prehistoric "sea monsters" of North Dakota. *Geo News, 41*(1), 19–25.

Holliday, C. M., & Gardner, N. M. (2012). A new eusuchian crocodyliform with novel cranial integument and its significance for the origin and evolution of Crocodylia. *PLOS ONE, 7*(1), e30471. <https://doi.org/10.1371/journal.pone.0030471>

Hollman, T. (2022). Reconstruction of *Livyatan melvillei*. Accessed July 6, 2024. <https://twitter.com/fishboy86164577/status/1584928023694159872>

Holtz, T. R., Jr. (1995). Re: Re: giganotosaurus...article from boston globe. Accessed July 27, 2024. <https://web.archive.org/web/20111111134307/http://dml.cmnh.org/1995Sep/msg00701.html>

Hone, D. W. E. (2012). Variation in the tail length of non-avian dinosaurs. *Journal of Vertebrate Paleontology, 32*(5), 1082–1089. <https://doi.org/10.1080/02724634.2012.680998>

Hopkins, S. S. B. (2008). Reassessing the mass of exceptionally large rodents using toothrow length and area as proxies for body mass. *Journal of Mammalogy, 89*(1), 232–243.

Hou, X., Bergström, J., & Yang, J. (2006). Distinguishing anomalocaridids from arthropods and priapulids. *Geological Journal, 41*(3-4), 259–269. <https://doi.org/10.1002/gj.1050>

Howard, H. (1957). A gigantic ‘toothed’ marine bird from the Miocene of California. *Santa Barbara Museum of Natural History Department of Geology Bulletin, 1*, 1–23.

Hunt, R. M., Jr. (2011). Evolution of large carnivores during the mid-Cenozoic of North America: the temnocyonine radiation (Mammalia, Amphicyonidae). *Bulletin of the American Museum of Natural History, 360*, 1–153.

Hunt, R. M., Jr., & Skolnick, R. (1996). The giant mustelid *Megalictis* from the Early Miocene carnivore dens at Agate Fossil Beds National Monument, Nebraska: earliest evidence of dimorphism in New World Mustelidae (Carnivora, Mammalia). *Contributions to Geology, University of Wyoming, 31*(1), 35–48.

Hurlburt, G., Heckert, A. B., & Farlow, J. O. (2003). Body mass estimates of phytosaurs (Archosauria: Parasuchidae) from the Petrified Forest Formation (Chinle Group: Revueltian) based on skull and limb bone measurements. *New Mexico Museum of Natural History and Science Bulletin, 24*, 105–113.

Ibrahim, N., Maganuco, S., Dal Sasso, C., Fabbri, M., Auditore, M., Bindellini, G., . . . Pierce, S. E. (2020). Tail-propelled aquatic locomotion in a theropod dinosaur. *Nature, 581*(7806), 67–70. <https://doi.org/10.1038/s41586-020-2190-3>

Ibrahim, N., Sereno, P. C., Dal Sasso, C., Maganuco, S., Fabbri, M., Martill, D. M., . . . Iurino, D. A. (2014). Semiaquatic adaptations in a giant predatory dinosaur. *Science, 345*(6204), 1613–1616. <https://doi.org/10.1126/science.1258750>

Iijima, M., & Kubo, T. (2020). Vertebrae-Based Body Length Estimation in Crocodylians and Its Implication for Sexual Maturity and the Maximum Sizes. *Integrative Organismal Biology, 2*(1), obaa042. <https://doi.org/10.1093/iob/obaa042>

Jeffery, J. (1998). *The morphology and phylogeny of the European members of Order Rhizodontida (Pisces: Sarcopterygii).* (PhD), University of Cambridge, Cambridge.

Jerison, H. J. (1973). *Evolution of the Brain and Intelligence*. New York: Academic Press.

Johanson, Z., & Ahlberg, P. E. (2001). Devonian rhizodontids and tristichopterids (Sarcopterygii; Tetrapodomorpha) from East Gondwana. *Transactions of the Royal Society of Edinburgh: Earth Sciences, 92*(1), 43–74. <https://doi.org/10.1017/S0263593300000043>

Johnson, A. E. (1979). Skeletal estimates of *Gigantopithecus* based on a Gorilla analogy. *Journal of Human Evolution, 8*(6), 585–587. <https://doi.org/10.1016/0047-2484(79)90111-8>

Juárez Valieri, R. D., Porfiri, J. D., & Calvo, J. O. (2010). New Information on *Ekrixinatosaurus novasi* Calvo et al 2004, a giant and massively-constructed Abelisauroid from the “Middle Cretaceous” of Patagonia. In J. O. Calvo, J. D. Porfiri, B. González Riga, & D. Dos Santos (Eds.), *Paleontologia y Dinosaurios en America Latina* (pp. 161-169).

Kaiser, A., & Klok, J. (2008). Do giant claws mean giant bodies? An alternative view on exaggerated scaling relationships. *Biology Letters, 4*(3), 279–280. <https://doi.org/10.1098/rsbl.2008.0015>

Kellner, A. W., & Campos, D. D. A. (2002). On a theropod dinosaur (Abelisauria) from the continental Cretaceous of Brazil. *Arquivos do Museu Nacional, 60*(3), 163-170.

Kimura, T., Hasegawa, Y., & Barnes, L. G. (2006). Fossil sperm whales (Cetacea, Physeteridae) from Gunma and Ibaraki prefectures, Japan; with observations on the Miocene fossil sperm whale *Scaldicetus shigensis* Hirota and Barnes, 1995. *Bulletin of the Gunma Museum of Natural History, 10*, 1–23.

Kingdon, J. (1988). *East African Mammals: An Atlas of Evolution in Africa, Volume 3, Part A: Carnivores*. Chicago: University of Chicago Press.

Klug, C., De Baets, K., Kröger, B., Bell, M. A., Korn, D., & Payne, J. L. (2014). Normal giants? Temporal and latitudinal shifts of Palaeozoic marine invertebrate gigantism and global change. *Lethaia, 48*(2), 267–288. <https://doi.org/10.1111/let.12104>

Klug, C., Schweigert, G., Hoffmann, R., Fuchs, D., Pohle, A., Weis, R., & De Baets, K. (2024). Anatomy and size of *Megateuthis*, the largest belemnite. *Swiss Journal of Palaeontology, 143*(1), 23. <https://doi.org/10.1186/s13358-024-00320-x>

Knutsen, E. M., Druckenmiller, P. S., & Hurum, J. H. (2012). A new species of *Pliosaurus* (Sauropterygia: Plesiosauria) from the Middle Volgian of central Spitsbergen, Norway. *Norwegian Journal of Geology, 92*, 235–258.

Konishi, T., Brinkman, D., Massare, J. A., & Caldwell, M. W. (2011). New exceptional specimens of *Prognathodon overtoni* (Squamata, Mosasauridae) from the upper Campanian of Alberta, Canada, and the systematics and ecology of the genus. *Journal of Vertebrate Paleontology, 31*(5), 1026–1046. <https://doi.org/10.1080/02724634.2011.601714>

Kosch, B. F. (1990). A Revision of the Skeletal Reconstruction of *Shonisaurus popularis* (Reptilia: Ichthyosauria). *Journal of Vertebrate Paleontology, 10*(4), 512–514.

Kosintsev, P., Mitchell, K. J., Devièse, T., van der Plicht, J., Kuitems, M., Petrova, E., . . . Lister, A. M. (2019). Evolution and extinction of the giant rhinoceros *Elasmotherium sibiricum* sheds light on late Quaternary megafaunal extinctions. *Nature Ecology & Evolution, 3*(1), 31–38. <https://doi.org/10.1038/s41559-018-0722-0>

Krause, D. W., Sampson, S. D., Carrano, M. T., & O'Connor, P. M. (2007). Overview of the history of discovery, taxonomy, phylogeny, and biogeography of *Majungasaurus crenatissumus* (Theropoda: Abelisauridae) from the Late Cretaceous of Madagascar. *Journal of Vertebrate Paleontology, 27*(sup2), 1-20. [https://doi.org/10.1671/0272-4634(2007)27[1:OOTHOD]2.0.CO;2](https://doi.org/10.1671/0272-4634(2007)27%5b1:OOTHOD%5d2.0.CO;2)

Ksepka, D. T. (2014). Flight performance of the largest volant bird. *Proceedings of the National Academy of Sciences, 111*(29), 10624–10629. <https://doi.org/10.1073/pnas.1320297111>

Ksepka, D. T., Fordyce, R. E., Ando, T., & Jones, C. M. (2012). New fossil penguins (Aves, Sphenisciformes) from the Oligocene of New Zealand reveal the skeletal plan of stem penguins. *Journal of Vertebrate Paleontology, 32*(2), 235–254. <https://doi.org/10.1080/02724634.2012.652051>

Kumiko. (2015). Brygmophyseter shigensis.jpg. *Wikimedia Commons*. Accessed July 29, 2024. <https://commons.wikimedia.org/wiki/File:Brygmophyseter_shigensis.jpg>

Lacovara, K. J., Lamanna, M. C., Ibiricu, L. M., Poole, J. C., Schroeter, E. R., Ullmann, P. V., . . . Novas, F. E. (2014). A Gigantic, Exceptionally Complete Titanosaurian Sauropod Dinosaur from Southern Patagonia, Argentina. *Scientific Reports, 4*(1), 6196. <https://doi.org/10.1038/srep06196>

Lamanna, M. C., Martínez, R. D., & Smith, J. B. (2002). A definitive abelisaurid theropod dinosaur from the early Late Cretaceous of Patagonia. *Journal of Vertebrate Paleontology, 22*(1), 58-69. [https://doi.org/10.1671/0272-4634(2002)022[0058:ADATDF]2.0.CO;2](https://doi.org/10.1671/0272-4634(2002)022%5b0058:ADATDF%5d2.0.CO;2)

Lambert, O., Bianucci, G., Post, K., de Muizon, C., Salas-Gismondi, R., Urbina, M., & Reumer, J. (2010). The giant bite of a new raptorial sperm whale from the Miocene epoch of Peru. *Nature, 466*(7302), 105–108. <https://doi.org/10.1038/nature09067>

Larivière, S. (2001). *Ursus americanus*. *Mammalian Species, 647*, 1–11.

Larramendi, A. (2015). Shoulder Height, Body Mass, and Shape of Proboscideans. *Acta Palaeontologica Polonica, 61*(3), 537–574. <https://doi.org/10.4202/app.00136.2014>

Larson, N. L. (2010). *Enchoteuthidae: Giant cephalopods from the Upper Cretaceous of the Western Interior.* Paper presented at the 16th Annual Tate Conference, Casper.

Larsson, H. C. E., & Sues, H.-D. (2007). Cranial osteology and phylogenetic relationships of *Hamadasuchus rebouli* (Crocodyliformes: Mesoeucrocodylia) from the Cretaceous of Morocco. *Zoological Journal of the Linnean Society, 149*(4), 533-567. <https://doi.org/10.1111/j.1096-3642.2007.00271.x>

Lawson, D. A. (1975). Pterosaur from the Latest Cretaceous of West Texas: Discovery of the Largest Flying Creature. *Science, 187*(4180), 947–948. <https://doi.org/10.1126/science.187.4180.947>

Lebedev, O. A. (2009). A new specimen of *Helicoprion* Karpinsky, 1899 from Kazakhstanian Cisurals and a new reconstruction of its tooth whorl position and function. *Acta Zoologica, 90*(s1), 171–182. <https://doi.org/10.1111/j.1463-6395.2008.00353.x>

Levy, D. L., & Heald, R. (2015). Biological Scaling Problems and Solutions in Amphibians. *Cold Spring Harbor Perspectives in Biology, 8*(1), a019166. <https://doi.org/10.1101/cshperspect.a019166>

Lingham-Soliar, T. (1995). Anatomy and Functional Morphology of the Largest Marine Reptile Known, *Mosasaurus hoffmanni* (Mosasauridae, Reptilia) from the Upper Cretaceous, Upper Maastrichtian of the Netherlands. *Philosophical Transactions: Biological Sciences, 347*(1320), 155–180.

Liston, J. (2003). Big Dead Fish, or just Big Dead-in-the-Water Ideas? Accessed June 12, 2024. <https://big-dead-fish.com/aftermath.php>

Liston, J., Newberry, M. G., Challandis, T. J., & Adams, C. E. (2013). Growth, age and size of the Jurassic pachycormid *Leedsichthys problematicus* (Osteichthyes: Actinopterygii). In G. Arratia, H.-P. Schultze, & M. V. H. Wilson (Eds.), *Mesozoic Fishes 5 - Global Diversity and Evolution* (pp. 145–175). München: Verlag Dr. Friedrich Pfeil.

Lomax, D. R., De la Salle, P., Massare, J. A., & Gallois, R. (2018). A giant Late Triassic ichthyosaur from the UK and a reinterpretation of the Aust Cliff ‘dinosaurian’ bones. *PLOS ONE, 13*(4), e0194742. <https://doi.org/10.1371/journal.pone.0194742>

Lomax, D. R., de la Salle, P., Perillo, M., Reynolds, J., Reynolds, R., & Waldron, J. F. (2024). The last giants: New evidence for giant Late Triassic (Rhaetian) ichthyosaurs from the UK. *PLOS ONE, 19*(4), e0300289. <https://doi.org/10.1371/journal.pone.0300289>

Long, J., Archer, M., Flannery, T., & Hand, S. (2003). *Prehistoric mammals of Australia and New Guinea*. Randwick: UNSW Press.

Long, J. A. (2024). *The Secret History of Sharks: The Rise of the Ocean's Most Fearsome Predators*. New York: Ballantine Books.

Lydekker, R. (1888). *Catalogue of the Fossil Reptilia and Amphibia in the British Museum. Part I. The orders Ornithosauria, Crocodilia, Dinosauria, Squamata, Rhynochocephalia, and Proterosauria.* (Vol. 1). London: British Museum of Natural History.

Lydekker, R. (1889). *Catalogue of the Fossil Reptilia and Amphibia in the British Museum. Part III. Chelonia* (Vol. 3). London: British Museum of Natural History.

MacPhee, R. D. E. (2018). *End of the Megafauna: The Fate of the World's Hugest, Fiercest, and Strangest Animals*. New York: W. W. Norton & Company.

Maisey, J. G., Bronson, A. W., Williams, R. R., & McKinzie, M. (2017). A Pennsylvanian ‘supershark’ from Texas. *Journal of Vertebrate Paleontology, 37*(3), e1325369. <https://doi.org/10.1080/02724634.2017.1325369>

Mallon, J. C., & Hone, D. W. E. (2024). Estimation of maximum body size in fossil species: A case study using *Tyrannosaurus rex*. *Ecology and Evolution, 14*(7), e11658. <https://doi.org/10.1002/ece3.11658>

Manger, W. L., Meeks, L. K., & Stephen, D. A. (1999). Pathologic Gigantism in Middle Carboniferous Cephalopods, Southern Midcontinent, United States. In F. Olóriz & F. J. Rodríguez-Tovar (Eds.), *Advancing Research on Living and Fossil Cephalopods: Development and Evolution Form, Construction, and function Taphonomy, Palaeoecology, Palaeobiogeography, Biostratigraphy, and Basin Analysis* (pp. 77–89). Boston, MA: Springer US.

Manzuetti, A., Perea, D., Jones, W., Ubilla, M., & Rinderknecht, A. (2020). An extremely large saber-tooth cat skull from Uruguay (late Pleistocene–early Holocene, Dolores Formation): body size and paleobiological implications. *Alcheringa: An Australasian Journal of Palaeontology, 44*(2), 332–339. <https://doi.org/10.1080/03115518.2019.1701080>

Martill, D. (1986). The world's largest fish. *Geology Today, 2*(2), 61–63. <https://doi.org/10.1111/j.1365-2451.1986.tb01022.x>

Martill, D. M. (1988). *Leedsichthys problematicus*, a giant filter-feeding teleost from the Jurassic of England and France. *Neues Jahrbuch für Geologie und Paläontologie Monatshefte, 11*, 670–680.

Martill, D. M., Jacobs, M. L., & Smith, R. E. (2023). A truly gigantic pliosaur (Reptilia, Sauropterygia) from the Kimmeridge Clay Formation (Upper Jurassic, Kimmeridgian) of England. *Proceedings of the Geologists' Association, 134*(3), 361–373. <https://doi.org/10.1016/j.pgeola.2023.04.005>

Martill, D. M., & Naish, D. (2000). *Walking With Dinosaurs: The Evidence*. London: BBC.

Marvin, N., & James, J. (2004). *Chased By Sea Monsters: Prehistoric Predators of the Deep*. London: DK Publishing.

Matthew, W. D. (1907). A lower Miocene fauna from South Dakota. *Bulletin of the American Museum of Natural History, 23*(9), 169–219.

May, M. L. (1982). Heat Exchange and Endothermy in Protodonata. *Evolution, 36*(5), 1051–1058. <https://doi.org/10.2307/2408082>

Mayr, G., & Rubilar-Rogers, D. (2010). Osteology of a new giant bony-toothed bird from the Miocene of Chile, with a revision of the taxonomy of Neogene Pelagornithidae. *Journal of Vertebrate Paleontology, 30*(5), 1313–1330. <https://doi.org/10.1080/02724634.2010.501465>

McGhee, G. R., Jr. (2018). *Carboniferous Giants and Mass Extinction: The Late Paleozoic Ice Age World*. New York: Columbia University Press.

McGowan, C., & Motani, R. (1999). A Reinterpretation of the Upper Triassic Ichthyosaur *Shonisaurus*. *Journal of Vertebrate Paleontology, 19*(1), 42–49.

McHenry, C. R. (2004). Re: Monster of Aramberri. *Dinosaur Mailing List*. Accessed July 14, 2024. <https://reptilis.net/DML/2004Apr/msg00337.html>

McHenry, C. R. (2009). *Devourer of gods: the palaeoecology of the Cretaceous pliosaur Kronosaurus queenslandicus.* (PhD), University of Newcastle, Newcastle.

McHenry, C. R. (2020). Comment on "In pursuit of giant pliosaurids and whale-sized ichthyosaurs". *Mark Witton's Blog*. Accessed July 14, 2024. <https://markwitton-com.blogspot.com/2020/04/in-pursuit-of-giant-pliosaurids-and.html>

McNamara, K., & Murray, P. (2010). *Prehistoric Mammals*. Perth: Western Australian Museum.

Meagher, M. (1986). *Bison bison*. *Mammalian Species, 266*, 1–8.

Méndez, A. H. (2012). The cervical vertebrae of the Late Cretaceous abelisaurid dinosaur *Carnotaurus sastrei*. *Acta Palaeontologica Polonica, 59*(3), 569-579. <https://doi.org/10.4202/app.2011.0129>

Merino-Rodo, D., & Janvier, P. (1986). Chondrichthyan and actinopterygian remains from theLower Permian Copacabana Formation of Bolivia. *Geobios, 19*(4), 479–493. <https://doi.org/10.1016/S0016-6995(86)80005-5>

Millien, V. (2008). The largest among the smallest: the body mass of the giant rodent *Josephoartigasia monesi*. *Proceedings of the Royal Society B: Biological Sciences, 275*(1646), 1953–1955. <https://doi.org/10.1098/rspb.2008.0087>

Millien, V., & Bovy, H. (2010). When teeth and bones disagree: body mass estimation of a giant extinct rodent. *Journal of Mammalogy, 91*(1), 11–18. <https://doi.org/10.1644/08-mamm-a-347r1.1>

Molina-Pérez, R., & Larramendi, A. (2020). *Dinosaur Facts and Figures: The Sauropods and Other Sauropodomorphs*. Princeton: Princeton University Press.

Molnar, R. E. (2004). *Dragons in the Dust: The Paleobiology of the Giant Monitor Lizard Megalania*. Bloomington: Indiana University Press.

Molnar, R. E., & Vasconcellos, F. M., de. (2016). Cenozoic dinosaurs in South America – revisited. *Memoirs of Museum Victoria, 74*, 363–377.

Morales-Betancourt, M. A. (2014). Comment on "A New Eusuchian Crocodyliform with Novel Cranial Integument and Its Significance for the Origin and Evolution of Crocodylia". Accessed July 12, 2024. <https://journals.plos.org/plosone/article/comment?id=10.1371/annotation/737b0348-798d-45fd-b430-37e9ad1a38f9>

Moreno-Bernal, J. W. (2007). Size and palaeoecology of giant Miocene South American crocodiles ( Archosauria: Crocodylia). *Journal of Vertebrate Paleontology, 27*(Supplement to No. 3), 120A.

Morlo, M. (1999). Niche structure and evolution in creodont (Mammalia) faunas of the European and North American Eocene. *Geobios, 32*(2), 297–305. <https://doi.org/10.1016/s0016-6995(99)80043-6>

Morlo, M., Miller, E. R., & El-Barkooky, A. N. (2007). Creodonta and Carnivora from Wadi Moghra, Egypt. *Journal of Vertebrate Paleontology, 27*(1), 145–159. [https://doi.org/10.1671/0272-4634(2007)27[145:CACFWM]2.0.CO;2](https://doi.org/10.1671/0272-4634(2007)27%5b145:CACFWM%5d2.0.CO;2)

Mortimer, M. (2004). Re: Largest Dinosaurs. Accessed July 27, 2024. <https://web.archive.org/web/20190913171408/http://dml.cmnh.org/2004Sep/msg00086.html>

Motani, R., & Pyenson, N. D. (2024). Downsizing a heavyweight: factors and methods that revise weight estimates of the giant fossil whale *Perucetus colossus*. *PeerJ, 12*, e16978. <https://doi.org/10.7717/peerj.16978>

Murphy, J. C., & Henderson, R. W. (1997). *Tales of giant snakes : a historical natural history of anacondas and pythons*. Malabar, Fla.: Krieger Pub. Co.

Murray, P. F., & Vickers-Rich, P. (2004). *Magnificent Mihirungs. The Colossal Flightless Birds of the Australian Dreamtime*. Bloomington, Indiana: Indiana University Press.

Musser, A. (2018). *Procoptodon goliath*. *Sydney, Australia*. Accessed April 25, 2024. <https://australian.museum/learn/australia-over-time/extinct-animals/procoptodon-goliah/>

Myers, T. J. (2001). Prediction of marsupial body mass. *Australian Journal of Zoology, 49*(2), 99–118.

Naish, D. (2012). Birds. In M. K. Brett-Surman, T. R. Holtz, Jr., & J. O. Farlow (Eds.), *The Complete Dinosaur* (pp. 379–424). Bloomington: Indiana University Press.

Naish, D. (2021). Reminiscing About Walking With Dinosaurs, Part 2. Accessed 2024, July 14. <https://tetzoo.com/blog/2021/7/3/reminiscing-about-walking-with-dinosaurs-part-2>

Naish, D., Noè, L. F., & Martill. (2001). Giant pliosaurs and the mysterious 'Megapleurodon'. *Dino Press*, 98–103.

National Academies of Sciences, Engineering, and Medicine. (2019). *Reproducibility and Replicability in Science*. Washington D.C.: The National Academies Press.

Naugolnykh, S. V. (2018). Artinskian (Early Permian) Sea Basin and Its Biota (Krasnoufimsk, Cis-Urals). *Stratigraphy and Geological Correlation, 26*(7), 734–754. <https://doi.org/10.1134/S0869593818070080>

Nelson, A., Engelman, R. K., & Croft, D. A. (2023). How to weigh a fossil mammal? South American notoungulates as a case study for estimating body mass in extinct clades. *Journal of Mammalian Evolution, 30*(3), 773–809. <https://doi.org/10.1007/s10914-023-09669-1>

Newberry, J. S. (1873). Fossil Fishes. In *Report of the Geological Survey of Ohio. Volume II. Geology and Paleontology* (Vol. 1, Part II, pp. 245–355). Columbus: Nevins and Myers, State Printers.

Newberry, J. S. (1875). Descriptions of fossil fishes. In *Report of the Geological Survey of Ohio. Volume II. Geology and Paleontology* (Vol. 2, Part II, pp. 1–64). Columbus: Nevins and Myers, State Printers.

Newberry, J. S. (1889). Paleozoic Fishes of North America. *Monographs of the U.S. Geological Survey, 16*, 1–228. <https://doi.org/10.5962/bhl.title.14705>

Newman, B. H. (1970). Stance and gait in the flesh-eating dinosaur Tyrannosaurus. *Biological Journal of the Linnean Society, 2*(2), 119–123. <https://doi.org/10.1111/j.1095-8312.1970.tb01707.x>

Nguyen, J. M. T., Boles, W. E., & Hand, S. J. (2010). New material of *Barawertornis tedfordi*, a dromornithid bird from the Oligo-Miocene of Australia, and its phylogenetic implications. *Records of the Australian Museum, 62*(1), 45–60. <https://doi.org/10.3853/j.0067-1975.62.2010.1539>

Nicholls, E. L., & Manabe, M. (2004). Giant ichthyosaurs of the Triassic—a new species of *Shonisaurus* from the Pardonet Formation (Norian: Late Triassic) of British Columbia. *Journal of Vertebrate Paleontology, 24*(4), 838–849. [https://doi.org/10.1671/0272-4634(2004)024[0838:GIOTTN]2.0.CO;2](https://doi.org/10.1671/0272-4634(2004)024%5b0838:GIOTTN%5d2.0.CO;2)

Noè, L. F., & Gómez-Pérez, M. (2022). Giant pliosaurids (Sauropterygia; Plesiosauria) from the Lower Cretaceous peri-Gondwanan seas of Colombia and Australia. *Cretaceous Research, 132*, 105122. <https://doi.org/10.1016/j.cretres.2021.105122>

Novas, F. E., Agnolín, F. L., Ezcurra, M. D., Porfiri, J., & Canale, J. I. (2013). Evolution of the carnivorous dinosaurs during the Cretaceous: The evidence from Patagonia. *Cretaceous Research, 45*, 174-215. <https://doi.org/10.1016/j.cretres.2013.04.001>

Novas, F. E., Chatterjee, S., Rudra, D. K., & Datta, P. M. (2010). *Rahiolisaurus gujaratensis*, n. gen. n. sp., A New Abelisaurid Theropod from the Late Cretaceous of India. In S. Bandyopadhyay (Ed.), *New Aspects of Mesozoic Biodiversity* (pp. 45-62). Berlin, Heidelberg: Springer Berlin Heidelberg.

O'Connor, P. M. (2007). The postcranial axial skeleton of *Majungasaurus crenatissimus* (Theropoda: Abelisauridae) from the Late Cretaceous of Madagascar. *Journal of Vertebrate Paleontology, 27*(sup2), 127-163. [https://doi.org/10.1671/0272-4634(2007)27[127:TPASOM]2.0.CO;2](https://doi.org/10.1671/0272-4634(2007)27%5b127:TPASOM%5d2.0.CO;2)

O'Shea, S., & Bolstad, K. (2019). Giant squid and colossal squid fact sheet. Accessed April 25, 2024. <https://tonmo.com/articles/giant-squid-and-colossal-squid-fact-sheet.18/>

O’Brien, H. D., Lynch, L. M., Vliet, K. A., Brueggen, J., Erickson, G. M., & Gignac, P. M. (2019). Crocodylian Head Width Allometry and Phylogenetic Prediction of Body Size in Extinct Crocodyliforms. *Integrative Organismal Biology, 1*(1), obz006. <https://doi.org/10.1093/iob/obz006>

Olson, S. R. (1985). The fossil record of birds. In D. Farner, J. King, & K. Parkes (Eds.), *Avian Biology* (Vol. 8, pp. 79–252). London: Academic Press.

Osborn, H. F., & Mook, C. C. (1921). *Camarasaurus*, *Amphicoelias*, and other sauropods of Cope. *Memoirs of the American Museum of Natural History, 3*(3), 248–347.

Paddle, R. (2000). *The Last Tasmanian Tiger: The History and Extinction of the Thylacine*. Cambridge ; New York: Cambridge University Press.

Paiva, A. L. S., Godoy, P. L., Souza, R. B. B., Klein, W., & Hsiou, A. S. (2022). Body size estimation of Caimaninae specimens from the Miocene of South America. *Journal of South American Earth Sciences, 118*, 103970. <https://doi.org/10.1016/j.jsames.2022.103970>

Pal, S., & Ayyasami, K. (2022). The lost titan of Cauvery. *Geology Today, 38*(3), 112-116. <https://doi.org/10.1111/gto.12390>

Parker, T. J., & Haswell, W. A. (1967). *Textbook of Zoology* (7th ed.). London: Macmillan Press LtD.

Patterson, B., & Pascual, R. (1968). The fossil mammal fauna of South America. *Quarterly Review of Biology, 43*(4), 409–451.

Paul, G. S. (1991). The many myths, some old, some new, of dinosaurology. *Modern Geology, 16*, 69-99.

Paul, G. S. (1994). Big sauropods—really, really big sauropods. *The Dinosaur Report, Fall,* 12–13.

Paul, G. S. (1997). Dinosaur models: the good, the bad, and using them to estimate the mass of dinosaurs. In D. L. Wolberg, E. Stump, & G. D. Rosenberg (Eds.), *DinoFest International Proceedings* (pp. 129–142). Philadelphias: The Academy of Natural Sciences.

Paul, G. S. (2010). *The Princeton Field Guide to Dinosaurs*. Princeton: Princeton University Press.

Paul, G. S. (2019). Determining the Largest Known Land Animal: A Critical Comparison of Differing Methods for Restoring the Volume and Mass of Extinct Animals. *Annals of Carnegie Museum, 85*(4), 335-358. <https://doi.org/10.2992/007.085.0403>

Paul, G. S. (2022). *The Princeton Field Guide to Mesozoic Sea Reptiles*. Princeton: Princeton University Press.

Paul, G. S. (2024). *Ichthyotitan*, new giant ichthyosaur from Late Triassic of UK + large ichthyosaur vertebra from Kama River, Russia. *Dinosaur Mailing Group*. Accessed July 25, 2024. <https://groups.google.com/g/dinosaurmailinggroup/c/95H0zq_H2t4/m/xnuCUkAjAAAJ>

Paul, G. S., & Larramendi, A. (2023). Body mass estimate of *Bruhathkayosaurus* and other fragmentary sauropod remains suggest the largest land animals were about as big as the greatest whales. *Lethaia, 56*(2), 1-11. <https://doi.org/10.18261/let.56.2.5>

Pei, W.-C. (1957). Giant Ape's Jaw Bone Discovered in China. *American Anthropologist, 59*(5), 834–838.

Persons, W. S., IV, Currie, P. J., & Erickson, G. M. (2020). An Older and Exceptionally Large Adult Specimen of *Tyrannosaurus rex*. *The Anatomical Record, 303*(4), 656–672. <https://doi.org/10.1002/ar.24118>

Pimiento, C., Kocáková, K., Mathes, G. H., Argyriou, T., Cadena, E.-A., Cooper, J. A., . . . Zanatta, C. (2024). The extinct marine megafauna of the Phanerozoic. *Cambridge Prisms: Extinction, 2*, 1–35. <https://doi.org/10.1017/ext.2024.12>

Pohle, A., & Klug, C. (2017). Body size of orthoconic cephalopods from the late Silurian and Devonian of the Anti‐Atlas (Morocco). *Lethaia, 51*(1), 126–148. <https://doi.org/10.1111/let.12234>

Pol, D., Leardi, J. M., Lecuona, A., & Krause, M. (2012). Postcranial anatomy of *Sebecus icaeorhinus* (Crocodyliformes, Sebecidae) from the Eocene of Patagonia. *Journal of Vertebrate Paleontology, 32*(2), 328–354. <https://doi.org/10.1080/02724634.2012.646833>

Pol, D., & Rauhut, O. W. M. (2012). A Middle Jurassic abelisaurid from Patagonia and the early diversification of theropod dinosaurs. *Proceedings of the Royal Society B: Biological Sciences, 279*(1741), 3170-3175. <https://doi.org/10.1098/rspb.2012.0660>

Polcyn, M. J., Jacobs, L. L., Araújo, R., Schulp, A. S., & Mateus, O. (2014). Physical drivers of mosasaur evolution. *Palaeogeography, Palaeoclimatology, Palaeoecology, 400*, 17–27. <https://doi.org/10.1016/j.palaeo.2013.05.018>

Prévost, C. (1855). Annonce de la découverte d'un oiseau fossile de taille gigantesque, trouvé à la partie infériure de l'argile plastique du terrain parisien. *Comptes rendus de l'Académie des Sciences, 40*, 554-557.

Prinsep, L. (1834). Note on the fossil bones on the Nerbudda valley discovered by Dr. G.G. Spilsbury near Narsinhpur. *Journal of the Asiatic Society of Bengal, 3*, 396–403.

Prothero, D. R. (2013). *Rhinoceros Giants: The Paleobiology of Indricotheres*. Bloomington: Indiana University Press.

Prothero, D. R. (2016). *The Princeton Field Guide to Prehistoric Mammals*. Princeton: Princeton University Press.

Prothero, D. R., & Schoch, R. M. (2002). *Horns, Tusks, and Flippers: The Evolution of Hoofed Mammals*. Baltimore: London.

Radford, T. (2005, September 8, 2005). Evolution's aviation ace is trumped. *The Guardian*. Retrieved from <https://www.theguardian.com/science/2005/sep/09/dinosaurs.highereducation>

Radinsky, L. (1977). Brains of early carnivores. *Paleobiology, 3*(4), 333–349. <https://doi.org/10.1017/S0094837300005509>

Randall, J. E. (1973). Size of the Great White Shark (*Carcharodon*). *Science, 181*(4095), 169–170. <https://doi.org/10.1126/science.181.4095.169>

Reynolds, P. S. (2002). How Big is a Giant? The Importance of Method in Estimating Body Size of Extinct Mammals. *Journal of Mammalogy, 83*(2), 321–332. <https://doi.org/10.1644/1545-1542(2002)083><0321:HBIAGT>2.0.CO;2

Rinderknecht, A., & Blanco, R. E. (2008). The largest fossil rodent. *Proceedings of the Royal Society B: Biological Sciences, 275*(1637), 923–928. <https://doi.org/10.1098/rspb.2007.1645>

Rinderknecht, A., Jones, W. W., Araújo, N., Grinspan, G., & Blanco, R. E. (2019). Bite force and body mass of the fossil rodent *Telicomys giganteus* (Caviomorpha, Dinomyidae). *Historical Biology, 31*(5), 644–652. <https://doi.org/10.1080/08912963.2017.1384475>

Romano, M., & Manucci, F. (2021). Resizing *Lisowicia bojani*: volumetric body mass estimate and 3D reconstruction of the giant Late Triassic dicynodont. *Historical Biology, 33*(4), 474–479. <https://doi.org/10.1080/08912963.2019.1631819>

Romer, A. S., & Lewis, A. D. (1959). A mounted skeleton of the giant plesiosaur *Kronosaurus*. *Breviora, 112*, 1–15.

Rovinsky, D. S., Evans, A. R., Martin, D. G., & Adams, J. W. (2020). Did the thylacine violate the costs of carnivory? Body mass and sexual dimorphism of an iconic Australian marsupial. *Proceedings of the Royal Society B: Biological Sciences, 287*(1933), 20201537. <https://doi.org/10.1098/rspb.2020.1537>

Rüber, L., Kottelat, M., Tan, H. H., Ng, P. K. L., & Britz, R. (2007). Evolution of miniaturization and the phylogenetic position of *Paedocypris*, comprising the world's smallest vertebrate. *BMC Evolutionary Biology, 7*(1), 38. <https://doi.org/10.1186/1471-2148-7-38>

Russell, D. A. (1967). Systematics and morphology of American mosasaurs. *Bulletin of the Peabody Museum of Natural History, 23*, 1–241.

Sallan, L., & Galimberti, A. K. (2015). Body-size reduction in vertebrates following the end-Devonian mass extinction. *Science, 350*(6262), 812–815. <https://doi.org/10.1126/science.aac7373>

Sampson, S. D., & Witmer, L. M. (2007). Craniofacial anatomy of *Majungasaurus crenatissimus* (Theropoda: Abelisauridae) from the Late Cretaceous of Madagascar. *Memoir (Society of Vertebrate Paleontology), 8*, 32-102.

Sánchez-Villagra, M. R., Aguilera, O., & Horovitz, I. (2003). The anatomy of the world's largest extinct rodent. *Science, 301*, 1708–1710. <https://doi.org/10.1126/science.1089332>

Sander, P. M., Chen, X., Cheng, L., & Wang, X. (2011). Short-snouted toothless ichthyosaur from China suggests Late Triassic diversification of suction feeding ichthyosaurs. *PLOS ONE, 6*(5), e19480. <https://doi.org/10.1371/journal.pone.0019480>

Sander, P. M., Griebeler Eva, M., Klein, N., Juarbe Jorge, V., Wintrich, T., Revell Liam, J., & Schmitz, L. (2021). Early giant reveals faster evolution of large body size in ichthyosaurs than in cetaceans. *Science, 374*(6575), eabf5787. <https://doi.org/10.1126/science.abf5787>

Sato, K., Sakamoto, K. Q., Watanuki, Y., Takahashi, A., Katsumata, N., Bost, C.-A., & Weimerskirch, H. (2009). Scaling of soaring seabirds and implications for flight abilities of giant pterosaurs. *PLOS ONE, 4*(4), e5400. <https://doi.org/10.1371/journal.pone.0005400>

Savage, R. J. G. (1973). *Megistotherium*, gigantic hyaenodont from Miocene of Gebel Zelten, Libya. *Bulletin of the British Museum (Natural History), Geology Series, 22*, 485–511.

Scheyer, T. M., Hutchinson, J. R., Strauss, O., Delfino, M., Carrillo-Briceño, J. D., Sánchez, R., & Sánchez-Villagra, M. R. (2019). Giant extinct caiman breaks constraint on the axial skeleton of extant crocodylians. *eLife, 8*, e49972. <https://doi.org/10.7554/eLife.49972>

Schmidt-Nielsen, K. (1984). *Scaling: Why is animal size so important?* Cambridge: Cambridge University Press.

Schwimmer, D. R. (2002). *King of the Crocodylians: The Paleobiology of Deinosuchus*. Bloomington: Indiana University Press.

Seebacher, F. (2001). A New Method to Calculate Allometric Length-Mass Relationships of Dinosaurs. *Journal of Vertebrate Paleontology, 21*(1), 51–60.

Sereno, P., & Larsson, H. (2009). Cretaceous Crocodyliforms from the Sahara. *ZooKeys, 28*, 1–143. <https://doi.org/10.3897/zookeys.28.325>

Sereno, P. C., Larsson, H. C. E., Sidor, C. A., & Gado, B. (2001). The Giant Crocodyliform *Sarcosuchus* from the Cretaceous of Africa. *Science, 294*(5546), 1516–1519. <https://doi.org/10.1126/science.1066521>

Sereno, P. C., Myhrvold, N., Henderson, D. M., Fish, F. E., Vidal, D., Baumgart, S. L., . . . Conroy, L. L. (2022). *Spinosaurus* is not an aquatic dinosaur. *eLife, 11*. <https://doi.org/10.7554/eLife.80092>

Sereno, P. C., Wilson, J. A., & Conrad, J. L. (2004). New dinosaurs link southern landmasses in the Mid–Cretaceous. *Proceedings of the Royal Society of London. Series B: Biological Sciences, 271*(1546), 1325-1330. <https://doi.org/10.1098/rspb.2004.2692>

Seymour, K. L. (1989). *Panthera onca*. *Mammalian Species, 340*, 1–9.

Simons, E. L., & Ettel, P. C. (1970). *Gigantopithecus*. *Scientific American, 222*(1), 76–87.

Simpson, G. G. (1976). *Penguins past and present, here and there*. New Haven: Yale University Press.

Simpson, G. G. (1980). *Splendid Isolation: the Curious History of South American Mammals*. New Haven, Connecticut: Yale University Press.

Smith, R. J. (2002). Estimation of Body Mass in Paleontology. *Journal of Human Evolution, 43*(2), 271–287. <https://doi.org/http://dx.doi.org/10.1006/jhev.2002.0573>

Soibelzon, L. H., & Schubert, B. W. (2011). The largest known bear, *Arctotherium angustidens*, from the early Pleistocene Pampean region of Argentina: with a discussion of size and diet trends in bears. *Journal of Paleontology, 85*(1), 69–75. <https://doi.org/10.1666/10-037.1>

Stearley, R. F., & Smith, G. S. (2016). Salmonid fishes from Mio-Pliocene lake sediments in the Western Snake River Plain and the Great Basin. *Miscellaneous Publications, Museum of Zoology, University of Michigan, 204*, 1–45.

Sternes, P. C., Jambura, P. L., Türtscher, J., Kriwet, J., Siversson, M., Feichtinger, I., . . . Kenshu. (2024). White shark comparison reveals a slender body for the extinct megatooth shark, *Otodus megalodon* (Lamniformes: Otodontidae). *Palaeontologia Electronica, 27*(1), a2. <https://doi.org/10.26879/1345>

Sternes, P. C., Wood, J. J., & Shimada, K. (2023). Body forms of extant lamniform sharks (Elasmobranchii: Lamniformes), and comments on the morphology of the extinct megatooth shark, *Otodus megalodon*, and the evolution of lamniform thermophysiology. *Historical Biology, 35*(1), 139–151. <https://doi.org/10.1080/08912963.2021.2025228>

Sulej, T., & Niedźwiedzki, G. (2019). An elephant-sized Late Triassic synapsid with erect limbs. *Science, 363*(6422), 78–80. <https://doi.org/10.1126/science.aal4853>

Switek, B. (2012). Paleontologists identify the Predator X fossil. Accessed July 14, 2024. <https://www.wired.com/story/predator-x/>

Tapanila, L., & Pruitt, J. (2013). Unraveling species concepts for the *Helicoprion* tooth whorl. *Journal of Paleontology, 87*(6), 965–983. <https://doi.org/10.1666/12-156>

Tapanila, L., & Pruitt, J. (2019). Redefining species concepts for the Pennsylvanian scissor tooth shark, *Edestus*. *PLOS ONE, 14*(9), e0220958. <https://doi.org/10.1371/journal.pone.0220958>

Tapanila, L., Pruitt, J., Pradel, A., Wilga, C. D., Ramsay, J. B., Schlader, R., & Didier, D. A. (2013). Jaws for a spiral-tooth whorl: CT images reveal novel adaptation and phylogeny in fossil Helicoprion. *Biology Letters, 9*(2), 20130057. <https://doi.org/10.1098/rsbl.2013.0057>

Tarlo, L. B. (1959). *Stretosaurus* gen. nov., a giant pliosaur from the Kimeridge Clay. *Palaeontology, 2*, 39–55.

Teichert, C., & Kummel, B. (1960). Size of endoceroid cephalopods. *Breviora, 128*, 1–7.

Teryaev, V. A. (1948). Geologicheskoe polozhenie gorbolobogo nosoroga (elasmoteriya). *Sovetskaya Geologia, 34*, 81–89.

Therrien, F., & Henderson, D. M. (2007). My theropod is bigger than yours … or not: estimating body size from skull length in theropods. *Journal of Vertebrate Paleontology, 27*(1), 108–115. [https://doi.org/10.1671/0272-4634(2007)27[108:MTIBTY]2.0.CO;2](https://doi.org/10.1671/0272-4634(2007)27%5b108:MTIBTY%5d2.0.CO;2)

Titov, V. V., Baigusheva, V. S., & Uchytel, R. S. (2021). The experience in reconstructing of the head of *Elasmotherium* (Rhinocerotidae). *Russian Journal of Theriology, 20*(2), 173–182. <https://doi.org/10.15298/rusjtheriol.20.2.06>

Toriño, P., Dutel, H., Soto, M., Norbis, W., Ezquerra, V., & Perea, D. (2024). Reconstructing an ancient fish: Three-dimensional skeletal restoration of the head of *Mawsonia* (Sarcopterygii, Actinistia) using CT scan, and an adjusted model for body size estimation in fossil coelacanths. *Journal of Anatomy, n/a*(n/a). <https://doi.org/10.1111/joa.14054>

Tortosa, T., Buffetaut, E., Vialle, N., Dutour, Y., Turini, E., & Cheylan, G. (2014). A new abelisaurid dinosaur from the Late Cretaceous of southern France: Palaeobiogeographical implications. *Annales de Paléontologie, 100*(1), 63-86. <https://doi.org/10.1016/j.annpal.2013.10.003>

Turner, A., & Antón, M. (2007). *Evolving Eden: An Illustrated Guide to the Evolution of the African Large-Mammal Fauna*. New York City: Columbia University Press.

Valenciano, A., Baskin, J. A., Abella, J., Pérez-Ramos, A., Álvarez-Sierra, M., Morales, J., & Hartstone-Rose, A. (2016). *Megalictis*, the Bone-Crushing Giant Mustelid (Carnivora, Mustelidae, Oligobuninae) from the Early Miocene of North America. *PLOS ONE, 11*(4), e0152430. <https://doi.org/10.1371/journal.pone.0152430>

Valenciano Vaquero, A. (2017). *Taxonomy, systematics and paleobiology of the giant mustelids (Mammalia, Carnivora, Mustelidae) from the Neogene of Europe, North America and Africa.* (PhD), Universidad Complutense de Madrid, Madrid.

Van Valkenburgh, B. (1990). Skeletal and dental predictors of body mass in carnivores. In J. Damuth & B. J. MacFadden (Eds.), *Body Size in Mammalian Paleobiology: Estimation and Biological Implications* (pp. 181–205). Cambridge: Cambridge University Press.

Venditti, C., Baker, J., & Barton, R. A. (2024). Co-evolutionary dynamics of mammalian brain and body size. *Nature Ecology & Evolution*. <https://doi.org/10.1038/s41559-024-02451-3>

Verdade, L. M. (2000). Regression equations between body and head measurements in the broad-snouted *Caiman* (*Caiman latirostris*). *Rev Bras Biol, 60*(3), 469–482. <https://doi.org/10.1590/s0034-71082000000300012>

Villalobos-Segura, E., Kriwet, J., Vullo, R., Stumpf, S., Ward, D. J., & Underwood, C. J. (2021). The skeletal remains of the euryhaline sclerorhynchoid †*Onchopristis* (Elasmobranchii) from the ‘Mid’-Cretaceous and their palaeontological implications. *Zoological Journal of the Linnean Society, 193*(2), 746–771. <https://doi.org/10.1093/zoolinnean/zlaa166>

Vinther, J., Porras, L., Young, F. J., Budd, G. E., & Edgecombe, G. D. (2016). The mouth apparatus of the Cambrian gilled lobopodian *Pambdelurion whittingtoni*. *Palaeontology, 59*(6), 841–849. <https://doi.org/10.1111/pala.12256>

Vizcaíno, S., & Fariña, R. A. (1999). On the flight capabilities and distribution of the giant Miocene bird *Argentavis magnificens* (Teratornithidae). *Lethaia, 32*, 271–278.

Vlachos, E., & Rabi, M. (2018). Total evidence analysis and body size evolution of extant and extinct tortoises (Testudines: Cryptodira: Pan-Testudinidae). *Cladistics, 34*(6), 652–683. <https://doi.org/10.1111/cla.12227>

Ward, P. D. (2006). *Out of Thin Air: Dinosaurs, Birds, and Earth's Ancient Atmosphere*: Joseph Henry Press.

Warren, A. A., & Hutchinson, M. N. (1983). The last labyrinthodont? A new brachyopoid (Amphibia, Temnospondyli) from the early Jurassic Evergreen Formation of Queensland, Australia. *Philosophical Transactions of the Royal Society B: Biological Sciences, 303*, 1-62.

Webb, G. J. W., & Messel, H. (1978). Morphometric analysis of *Crocodylus porosus* from the north coast of Arnhem Land, Northern Australia. *Australian Journal of Zoology, 26*(1), 1–27.

Webb, R. E. (1998). Megamarsupial extinction: the carrying capacity argument. *Antiquity, 72*(275), 46–55. <https://doi.org/10.1017/S0003598X00086269>

Wedel, M. J. (2008). SV-POW! showdown: sauropods vs whales. *Sauropod Vertebra Picture of the Week*. Accessed July 27, 2024. <https://svpow.com/2008/05/20/sv-pow-showdown-sauropods-vs-whales/>

Weidenreich, F. (1946). *Apes, giants, and man*. Chicago: University of Chicago.

Welker, F., Ramos-Madrigal, J., Kuhlwilm, M., Liao, W., Gutenbrunner, P., de Manuel, M., . . . Cappellini, E. (2019). Enamel proteome shows that *Gigantopithecus* was an early diverging pongine. *Nature, 576*(7786), 262–265. <https://doi.org/10.1038/s41586-019-1728-8>

Wellnhofer, P. (1970). Die Pterodactyloidea (Pterosauria) der Oberjura-Plattenkalke Süddeutschlands. *Bayerische Akademie der Wissenschaften, Mathematisch- Wissenschaftlichen Klasse, Abhandlugen, 141*, 1–133.

Whittington, H. B., & Briggs, D. E. G. (1997). The largest Cambrian animal, *Anomalocaris*, Burgess Shale, British-Columbia. *Philosophical Transactions of the Royal Society of London. B, Biological Sciences, 309*(1141), 569–609. <https://doi.org/10.1098/rstb.1985.0096>

Willoughby, D. P. (1978). *All about gorillas*. South Brunswick: A. S. Barnes.

Wilson, J. A., Sereno, P. C., Srivastava, S., Bhatt, D. K., Khosla, A., & Sahni, A. (2003). A new abelisaurid (Dinosauria, Theropoda) from the Lameta Formation (Cretaceous, Maastrictian) of India. *31*(1), 1-42.

Witton, M. P. (2008). A new approach to determining pterosaur body mass and its implications for pterosaur flight. *Zitteliana, B28*, 143–159.

Witton, M. P. (2010). *Pteranodon* and beyond: the history of giant pterosaurs from 1870 onwards. *Geological Society, London, Special Publications, 343*(1), 313–323. <https://doi.org/10.1144/sp343.19>

Witton, M. P. (2013). *Pterosaurs: Natural History, Evolution, Anatomy*. Princeton: Princeton University Press.

Witton, M. P. (2018). Those terrific pelagornithids. *Mark P. Witton's Blog*. Accessed March 24, 2024. <https://markwitton-com.blogspot.com/2018/08/those-terrific-pelagornithids.html>

Witton, M. P. (2019). We need to talk about teratorns. *Mark P. Witton's Blog*. Accessed July 8, 2024. <https://markwitton-com.blogspot.com/2019/08/we-need-to-talk-about-teratorns.html>

Witton, M. P., & Habib, M. B. (2010). On the Size and Flight Diversity of Giant Pterosaurs, the Use of Birds as Pterosaur Analogues and Comments on Pterosaur Flightlessness. *PLOS ONE, 5*(11), e13982. <https://doi.org/10.1371/journal.pone.0013982>

Wood, R. C. (1976). *Stupendemys geographicus*, the world's largest turtle. *Breviora, 436*, 1–31.

Woodruff, A., & Johnson-Random, E. (2024). *Prehistoric World: 1,200 Incredible Mammals and Discoveries from the Mesozoic*. Nashville: Applesauce Press.

Woodward, A. R., White, J. H., & Linda, S. B. (1995). Maximum Size of the Alligator (*Alligator mississippiensis*). *Journal of Herpetology, 29*(4), 507–513. <https://doi.org/10.2307/1564733>

Woodward, S. A. (1905). *A Guide to the Fossil Reptiles, Amphibians, and Fishes in the Department of Geology and Palaeontology of the British Museum (Natural History)* (Eighth ed.). London: British Museum of Natural History.

Woodward, S. A. (1917). Alfred Nicholson Leeds, F.G.S. *Geological Magazine, 6*(4), 478–480.

Worthy, T. H., & Holdaway, R. N. (2002). *The Lost World of the Moa: Prehistoric Life of New Zealand*. Bloomington: Indiana University Press.

Wroe, S. (2002). A review of terrestrial mammalian and reptilian carnivore ecology in Australian fossil faunas, and factors influencing their diversity: the myth of reptilian domination and its broader ramifications. *Australian Journal of Zoology, 50*(1), 1–24.

Wroe, S., Clausen, P., McHenry, C., Moreno, K., & Cunningham, E. (2007). Computer simulation of feeding behaviour in the thylacine and dingo as a novel test for convergence and niche overlap. *Proceedings of the Royal Society B: Biological Sciences, 274*(1627), 2819–2828. <https://doi.org/10.1098/rspb.2007.0906>

Wroe, S., Crowther, M., Dortch, J., & Chong, J. (2004). The size of the largest marsupial and why it matters. *Proceedings of the Royal Society of London. Series B: Biological Sciences, 271*(suppl_3), S34–S36. <https://doi.org/10.1098/rsbl.2003.0095>

Wroe, S., Myers, T., Seebacher, F., Kear, B., Gillespie, A., Crowther, M., & Salisbury, S. (2003). An alternative method for predicting body mass: the case of the Pleistocene marsupial lion. *Paleobiology, 29*(3), 403–411. <https://doi.org/10.1666/0094-8373(2003)029><0403:aamfpb>2.0.co;2

Wroe, S., Myers, T. J., Wells, R. T., & Gillespie, A. K. (1999). Estimating the weight of the Pleistocene marsupial lion, *Thylacoleo carnifex* (Thylacoleonidae: Marsupialia): implications for the ecomorphology of a marsupial super-predator and hypotheses of impoverishment of Australian marsupial carnivore faunas. *Australian Journal of Zoology, 47*, 489–498.

Xing, L., O’Connor, J. K., Schmitz, L., Chiappe, L. M., McKellar, R. C., Yi, Q., & Li, G. (2020). Hummingbird-sized dinosaur from the Cretaceous period of Myanmar. *Nature, 579*(7798), 245–249. <https://doi.org/10.1038/s41586-020-2068-4>

Yadagiri, P., & Ayyasami, K. (1987). A carnosaurian dinosaur from the Kallamedu Formation (Maestrichtian horizon), Tamilnadu. *Geological Society of India Special Publication, 11*(1), 523-528.

Young, B., Dunstone, R. L., Senden, T. J., & Young, G. C. (2013). A gigantic sarcopterygian (tetrapodomorph lobe-finned fish) from the upper Devonian of Gondwana (Eden, New South Wales, Australia). *PLOS ONE, 8*(3), e53871. <https://doi.org/10.1371/journal.pone.0053871>

Young, M. T., Hua, S., Steel, L., Foffa, D., Brusatte, S. L., Thüring, S., . . . De Andrade, M. B. (2014). Revision of the Late Jurassic teleosaurid genus *Machimosaurus* (Crocodylomorpha, Thalattosuchia). *Royal Society Open Science, 1*(2), 140222. <https://doi.org/10.1098/rsos.140222>

Young, M. T., Rabi, M., Bell, M. A., Foffa, D., Steel, L., Sachs, S., & Peyer, K. (2016). Big-headed marine crocodyliforms and why we must be cautious when using extant species as body length proxies for long-extinct relatives. *Palaeontologia Electronica, 19.3.30A*, 1–14. <https://doi.org/10.26879/648>

Zangerl, R. (1981). *Chondrichthyes I. Paleozoic Elasmobranchii* (Vol. 3A). New York: Gustav Fisher.

Zhang, Y., & Harrison, T. (2017). *Gigantopithecus blacki*: a giant ape from the Pleistocene of Asia revisited. *American Journal of Physical Anthropology, 162*(S63), 153–177. <https://doi.org/10.1002/ajpa.23150>

Zhao, R. J. (2024). Body reconstruction and size estimation of plesiosaurs. *bioRxiv*, 2024.2002.2015.578844. <https://doi.org/10.1101/2024.02.15.578844>

Zhegallo, V., Kalandadze, N., Shapovalov, A., Bessudnova, Z., Noskova, N., & Tesakova, E. (2005). On the fossil rhinoceros *Elasmotherium* (including the collections of the Russian Academy of Sciences). *Cranium, 22*(1), 17–40.

Zhu, M., Ahlberg, P. E., Zhao, W.-J., & Jia, L.-T. (2017). A Devonian tetrapod-like fish reveals substantial parallelism in stem tetrapod evolution. *Nature Ecology & Evolution, 1*(10), 1470–1476. <https://doi.org/10.1038/s41559-017-0293-5>

Graphical abstract

Reconstructing the body size and form of extinct animals is of vital importance to our understanding of macroevolution and palaeontology. This is often done using anatomical proxies where extinct species are known only from fragmentary remains. However, there are many limitations influencing the selection of proxy taxa that are frequently overlooked. Here we discuss these limitations and mitigation measures that should be applied to overcome them.

1. Vertebral estimates produced a value of 8.02 ± 0.57 m, whereas cranial and appendicular estimates produced smaller values of 6.03 m and 6.67 ± 0.34 m, respectively. Disparities between cranial/vertebral/appendicular estimates were usually not this large for other taxa. [↑](#footnote-ref-1)
2. Grillo and Delcourt (2017) note that Krause et al. (2017) do not tie their estimate of 6-7 m for *Majungasaurus crenatissimus* to a single specimen, instead reporting it as an average length for the species as a whole. Thus, they compared their estimates individually against this composite value. Notably, Grillo and Delcourt (2017) estimated a length of 4.55 m for UA 8678 and 5.72 m for FMNH PR 2100 based on published reconstructions. This also does not consider isolated material (MNHN.MAJ 4) which Sampson and Witmer (2007) in the same edited volume describe as representing an individual about 25% larger than FMNH PR 2100. Given Sampson and Witmer (2007) estimate the size of this individual to be 8–11 m, this suggests an approximate length of ~6.4–8.8 m for FMNH PR 2100, largely agreeing with the estimate of Krause et al. (2017) and allowing more direct comparison with the values in Grillo and Delcourt (2017). [↑](#footnote-ref-2)
3. Farke and Sertich (2013) produced two total length estimates for *Dahalokely*, one of 3.5 ± 0.9 m assuming proportions similar to abelisaurids, and one of 4.9 ± 0.7 m assuming proportions similar to noasaurids. They considered the shorter estimate to be more reasonable given the short, abelisaurid-like nature of the vertebrae despite recovering *Dahalokely* as a noasaurid, a decision which was probably prescient given later studies have recovered this taxon as an abelisaurid (Fillipi et al. 2016; Gianechini et al. 2020). Given this, we evaluate the difference in estimates relative to the 3.5 m estimate favored by Farke and Sertich (2013). [↑](#footnote-ref-3)
